# Novel Route to Cationic Palladium(II)–Cyclopentadienyl Complexes Containing Phosphine Ligands and Their Catalytic Activities

**DOI:** 10.3390/molecules28104141

**Published:** 2023-05-17

**Authors:** Dmitry S. Suslov, Mikhail V. Bykov, Marina V. Pakhomova, Timur S. Orlov, Zorikto D. Abramov, Anastasia V. Suchkova, Igor A. Ushakov, Pavel A. Abramov, Alexander S. Novikov

**Affiliations:** 1 Research Institute of Oil and Coal Chemical Synthesis, Irkutsk State University, ul. K. Marksa, 1, Irkutsk 664003, Russia; bykov@chem.isu.ru (M.V.B.); m-belova@bk.ru (M.V.P.); tim53145314@gmail.com (T.S.O.); murasakibara_atsushi@bk.ru (Z.D.A.); nasyaya27@gmail.com (A.V.S.); 2 School of High Technologies, National Research Irkutsk State Technical University, Lermontov St., 83, Irkutsk 664074, Russia; 3 A.E. Favorsky Irkutsk Institute of Chemistry SB RAS, Favorsky St., 1, Irkutsk 664033, Russia; igor-papa71@mail.ru; 4 Nikolaev Institute of Inorganic Chemistry SB RAS, pr-kt Akad. Lavrentieva, 3, Novosibirsk 630090, Russia; abramov@niic.nsc.ru; 5 Research School of Chemistry and Applied Biomedical Sciences, Tomsk Polytechnic University, Tomsk 634034, Russia; 6 Institute of Chemistry, Saint Petersburg State University, Universitetskaya Nab., 7/9, Saint Petersburg 199034, Russia; 7 Research Institute of Chemistry, Peoples’ Friendship University of Russia (RUDN University), Miklukho-Maklaya St., 6, Moscow 117198, Russia

**Keywords:** palladium, cyclopentadienyl, acetylacetonate, 1,3-butadiene, telomerization, phenylacetylene

## Abstract

The Pd(II) complexes [Pd(Cp)(L)_n_]_m_[BF_4_]_m_ were synthesized via the reaction of cationic acetylacetonate complexes with cyclopentadiene in the presence of BF_3_∙OEt_2_ (*n* = 2, *m* = 1: L = PPh_3_ (**1**), P(*p*-Tol)_3_, *tris*(*ortho*-methoxyphenyl)phosphine (TOMPP), *tri*-2-furylphosphine, *tri*-2-thienylphosphine; *n* = 1, *m* = 1: L = dppf, dppp (**2**), dppb (**3**), 1,5-bis(diphenylphosphino)pentane; *n* = 1, *m* = 2 or 3: 1,6-bis(diphenylphosphino)hexane). Complexes **1**–**3** were characterized using X-ray diffractometry. The inspection of the crystal structures of the complexes enabled the recognition of (Cp^–^)⋯(Ph-group) and (Cp^–^)⋯(CH_2_-group) interactions, which are of C–H…π nature. The presence of these interactions was confirmed theoretically via DFT calculations using QTAIM analysis. The intermolecular interactions in the X-ray structures are non-covalent in origin with an estimated energy of 0.3–1.6 kcal/mol. The cationic palladium catalyst precursors with monophosphines were found to be active catalysts for the telomerization of 1,3-butadiene with methanol (*TON* up to 2.4∙10^4^ mol 1,3-butadiene per mol Pd with chemoselectivity of 82%). Complex [Pd(Cp)(TOMPP)_2_]BF_4_ was found to be an efficient catalyst for the polymerization of phenylacetylene (PA) (catalyst activities up to 8.9 × 10^3^ g_PA_·(mol_Pd_·h)^−1^ were observed)

## 1. Introduction

The elucidation of ferrocene’s structure in 1952 by Wilkinson, Woodward, and Fischer [1,2] inspired chemists to extensively study transition metal complexes with a cyclopentadienyl (Cp) ligand. This ligand exhibits unique electronic properties and often behaves as a stable three-coordinate spectator ligand [3]. Furthermore, Cp proved to be a good supporting ligand for catalyst design [4]. Although in most cases, industrial application requires the use of heterogeneous, supported catalysts, homogeneous and metal nanoparticle catalysts [5,6,7], which may present an intermediate option between homogeneous and heterogeneous catalysts, continue to evolve [8]. A famous example of this is metallocene catalysts (with titanium-family metals) that have been applied in the industrial production of α-olefin polymers owing to their single reaction active center and high catalytic activity [9,10,11,12].

**Scheme 1:**
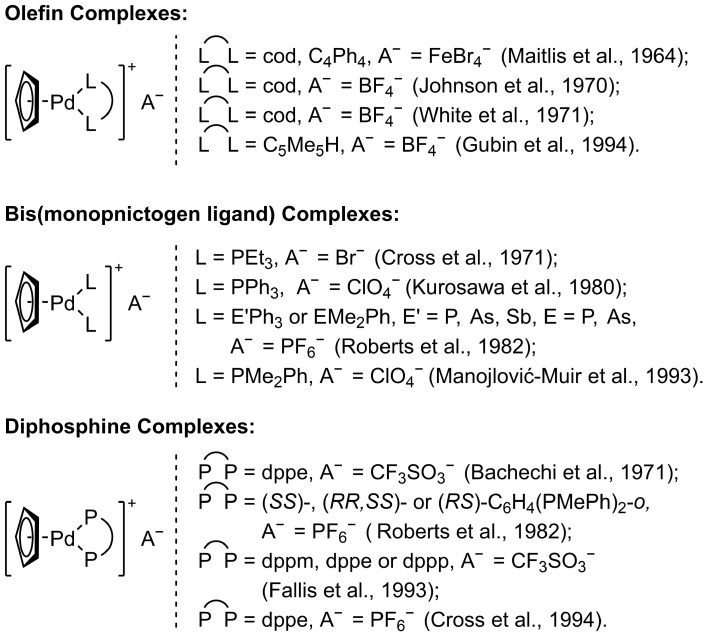
Examples of the reported cationic η^5^-cyclopentadienyl Pd-complexes [17,26,27,28,29,30,31,32,33,34,35,36].

Although a number of η^5^-cyclopentadienyl palladium complexes have been reported, they are relatively few compared to those of other platinum-group metals [4,13]. Most of these are neutral palladium(II) species (e.g., see [13,14,15,16,17,18,19,20,21,22,23,24]), including the most well-known [25] (η^3^-allyl)(η^5^-cyclopentadienyl)palladium(II)complex [21,22]. Cationic palladium complexes containing the η^5^-cyclopentadienyl ligand are rare [17,26,27,28,29,30,31,32,33,34,35,36] (Scheme 1), and only six of them have been characterized using X-ray diffraction methods [26,27,28,32,34,35]. Consequently, the chemistry of η^5^-Cp Pd compounds is not well developed. There is an opinion [19] that this is due to a lack of suitable methods to introduce the Cp ligand to palladium. The main method of synthesis involves the interaction of CpTl with palladium halides. Cyclopentadienylthallium is a highly toxic chemical [37] which is sometimes used in large excess [19]. Another synthetic route, reported by Roberts et al. [35], includes the reaction of cyclopentadiene with the dicationic solvent complexes of palladium to produce monocationic η^5^-cyclopentadienyl complexes.

**Scheme 2:**
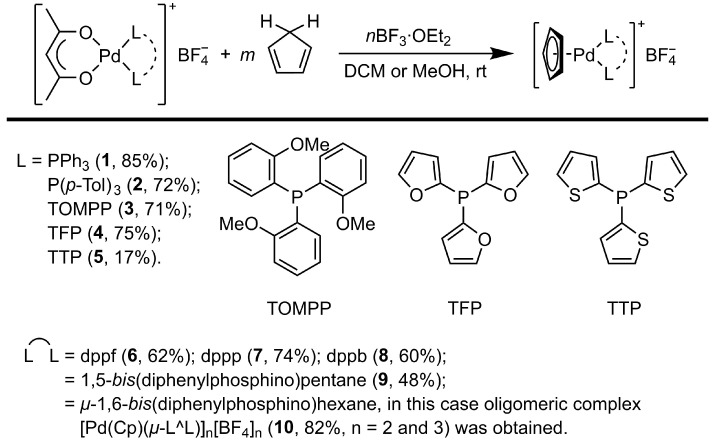
Synthesis of new cationic η^5^-cyclopentadienyl Pd-complexes (**1**–**10**, isolated yields are given in parentheses) with phosphine (L) or diphosphine ligands (L^L).

In this paper, we present a novel route for the syntheses of cationic η^5^-cyclopentadienyl palladium(II) complexes, featuring mono- and bidentate tertiary phosphines ligands. This chemistry is summarized in Scheme 2. Ten new compounds have been fully characterized, and the crystal structures of [Pd(Cp)(PPh_3_)_2_]BF_4_, [Pd(Cp)(dppp)]BF_4_, and [Pd(Cp)(dppb)]BF_4_ have been determined via X-ray diffraction. NMR spectroscopy features of the prepared cationic complexes are also discussed. Additionally, we disclose our findings on the catalytic activity of the novel complexes in the polymerization of phenylacetylene (PA) and telomerization of butadiene (BD) with methanol.

## 2. Results and Discussion

### 2.1. Synthesis of Palladium(II) Complexes

We have found that complexes **1**–**10** (Scheme 2) can be prepared through reacting cationic acetylacetonate complexes with cyclopentadiene in the presence of BF_3_∙OEt_2_. The starting palladium complexes are easily prepared in high yields from readily available [Pd(acac)(MeCN)_2_]BF_4_ [38,39]. Use of methanol as a solvent was found to be more convenient than non-coordinating dichloromethane or 1,2-dichloroethane. For example, the reaction of [Pd(acac)(PPh_3_)_2_]BF_4_ with 1 eq. of BF_3_∙OEt_2_ and 1 eq. of CpH in CH_2_Cl_2_ led to the formation of desired complex **1** within 1 h. However, in this case, the reaction was complicated by a side reaction. In the IR and ^1^H NMR spectra of the precipitate containing complex **1**, signals that can be attributed to cyclopentadiene/dicyclopentadiene (**DCPD**) oligomers were observed. As a consequence, the preparation of complex **1** required additional recrystallization of the reaction product from a MeCN/Et_2_O mixture, which reduced the yield of the complex. The review [40] indicated that DCPD can be oligomerized using initiators of carbocationic polymerization. We carried out the reaction between cyclopentadiene (CpH) and BF_3_∙OEt_2_ in the absence of a palladium compound and obtained a white powder. According to IR and ^1^H NMR data, this can be attributed to a mixture of *exo*-2,3- and *exo*-2,7-polyDCPD [41,42]. The side process of oligomerization does not occur in methanol, but a longer reaction time and the use of an excess of CpH and BF_3_∙OEt_2_ are required to increase the yield of complex **1**. On the other hand, the formation of complex **1** was not observed in more coordinating solvents such as MeCN or DMSO.

The new compounds (**1**–**10**) were characterized using multinuclear and two-dimensional (COSY, NOESY, HMBC, HSQC) NMR, IR, and UV spectroscopy; ESI-MS; and elemental analysis (see Supporting Information File (SI) for details). The obtained complexes appear to be stable solids even in air at ambient temperature, although they decompose slowly in solution. For example, solutions of complexes **1**–**10** in acetonitrile or MeCN/toluene mixtures were stable for days at room temperature, while those in MeOH, CH_2_Cl_2_, or CHCl_3_ decomposed within 10 h, forming an unidentified black precipitate.

In all cases, coordination of the phosphines caused a downfield shift Δ(δ_complex_ − δ_ligand_) of the ^31^P{^1^H} NMR signals in the range of 34.4 to 55.4 ppm (Table 1). It should be noted that the ^31^P{^1^H} and ^1^H NMR spectra of complex **3** are severely broadened at room temperature (e.g., Δν_P_(FWHM) = 365 Hz at δ_P_ = 8.2 ppm), indicating that the complex shows dynamic exchange, most likely between “TOMPP-Pd”-rotamers in solution. The NMR signal broadening observed for complex **3** is comparable to that observed for *cis*-[Pd((1−3η)-but-2-en-1-yl)(TOMPP)_2_]BF_4_ [43].

The δ values obtained from ^1^H and ^13^C NMR spectra of compounds **1**–**10** are consistent with NMR data for known palladium complexes with the same phosphine ligands [38,50,51,52,53,54]. In each case, the ^1^H NMR signal for the cyclopentadienyl group appears as a triplet (*J*_HP_ = 2.1–2.3 Hz), due to coupling to the two phosphorus atoms (Table 2). This was additionally confirmed in the phosphorus-decoupled ^1^H{^31^P} NMR spectra, where these signals appeared as singlets. The ^1^H NMR chemical shifts of the C_5_H_5_ group were dependent on the nature of the phosphine ligand. The C_5_H_5_ resonance in complex **3**, for example, appears at 5.12 ppm (shielded by an aromatic ring current) when using two bulky *tris*(*ortho*-methoxyphenyl)phosphine ligands (cone angle θ =176° [55]), while the same group signal for complex **4** with non-bulky *tris*(2-furyl)phosphine was observed at 6.04 ppm. In addition, the ^13^C{^1^H} NMR spectra of compounds **1**–**9** revealed diagnostic carbon peaks of Cp ligands as triplets at 100–105 ppm (for complex **10,** a broadened asymmetric singlet from C_Cp_ was observed at 101.5 ppm). In the aromatic region of the ^13^C{^1^H} NMR spectra, the signals corresponding to the *ipso*-C, *ortho*-C, and *meta*-C of the PAr-moieties were observed as the expected virtual triplets [56], whereas signals for *para*-C were still observed as singlets.

The NMR spectra of compounds **9** and **10** suggest that they exist in different isomeric forms in solution. In particular, the ^1^H and ^13^C NMR spectra of compound **10** display three distinct sets of resonances in C_5_H_5_ group region. The major isomer gave resonances at δ 5.78 (t, *J* = 2.0 Hz, C_5_*H*_5_) and 101.51 (s, br., *C*_5_H_5_), while the minor isomers were assigned to resonances at δ 5.76–5.69 (m), 5.61 (t, *J* = 2.0 Hz, C_5_*H*_5_), and 102.18 (s, br., *C*_5_H_5_). The ^31^P{^1^H} NMR spectrum of complex **10** displayed a singlet at δ 28.61 for the major isomer, while singlets at δ 28.39–28.22, 27.11 were observed for the minor isomers in a 6:4 intensity ratio. We assume that the isomers observed in solution are due to presence of μ-P-type coordination dimers and oligomers. The ESI-MS isotope distribution data (Figure S76, SI) for compound **10** do not contradict this assumption. The distribution can be interpreted in favor of the presence of bi- and trinuclear palladium complexes in solution. A quantitative calculation of the corresponding intensoids in the enviPat program [57] gave the ratio of dimers and trimers as 4 to 6. From ESI-MS and NMR data for complex **9**, mononuclear compounds were mainly found in solution, and the suitable isotope distribution ratio was modeled as [M_1_]^+^:[M_2_]^2+^:[M_3_]^3+^ = 9.5:0.1:0.4. In the case of compounds **1**–**8**, ESI-MS data confirmed the formation of the expected mononuclear complex cations.

**Scheme 3:**
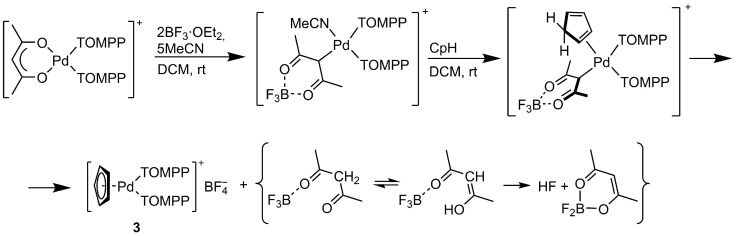
Plausible route for the synthesis of complex **3**. Anion [BF_4_]^−^ is omitted for clarity.

Although the mechanism of synthesis of cationic cyclopentadienyl palladium complexes **1**–**10** from acetylacetonate precursors is not known, it is assumed that the process occurs stepwise, with an early step being the reaction of boron trifluoride molecules with the acetylacetonate ligand to produce a *κ*^1^-C-acac-Pd species, which then reacts with cyclopentadiene. Evidence supporting this hypothesis has been obtained via examining the stoichiometric reaction of [Pd(*κ*^2^-*O*,*O*′-acac)(TOMPP)_2_]BF_4_ with BF_3_∙OEt_2_ (Scheme 3). When [Pd(*κ*^2^-*O*,*O*′-acac)(TOMPP)_2_]BF_4_ is reacted with 2 equivalents of BF_3_∙OEt_2_ in the presence of 5 equivalents of MeCN as a stabilizing agent in CH_2_Cl_2_, a yellow-orange precipitate is formed. Although this intermediate could not be obtained pure, ^1^H, ^13^C, ^31^P, ^19^F, and ^11^B NMR and ESI-MS data (see SI) suggest it is mostly [Pd(*κ*^1^-C-acac∙BF_3_)(MeCN)(TOMPP)_2_]BF_4_. The presence of carbon-ligated acac is indicated by the doublet peaks (*J* = 3.9 Hz) of the methine carbon of the acac ligand at 101.96 ppm in the ^13^C{^1^H} NMR spectrum. The ^1^H NMR spectrum of the residue in CDCl_3_ shows signals at 2.30 and 6.00 ppm from the methyl and methine protons of the *κ*^1^-C-acac ligand and characteristic resonance at 3.81 ppm from the 2-methoxy-group of the TOMPP ligand. The ^31^P{^1^H} NMR spectrum shows a singlet from the major product at 32.7 ppm shifted downfield when compared to [Pd(acac)(TOMPP)_2_]BF_4_ (17.5 ppm [50]). The obtained precipitate also displays characteristic resonances in the ^13^C{^1^H} NMR spectrum for CO and CH_3_ groups of the *κ*^1^-C-acac ligand (at 192.49 and 24.22 ppm). The ^19^F NMR spectrum shows two major sets of signals with an intensity ratio of approximately 3:4, corresponding to the BF_3_ and [BF_4_]^−^ structural fragments in the intermediate. Furthermore, upon addition of cyclopentadiene to the intermediate in CH_2_Cl_2_, green complex **3** is formed along with BF_2_(acac), which was detected in the GC-MS spectra of the solution.

### 2.2. X-ray Crystal Structures of ***1***, ***7***, and ***8***

**Scheme 4:**
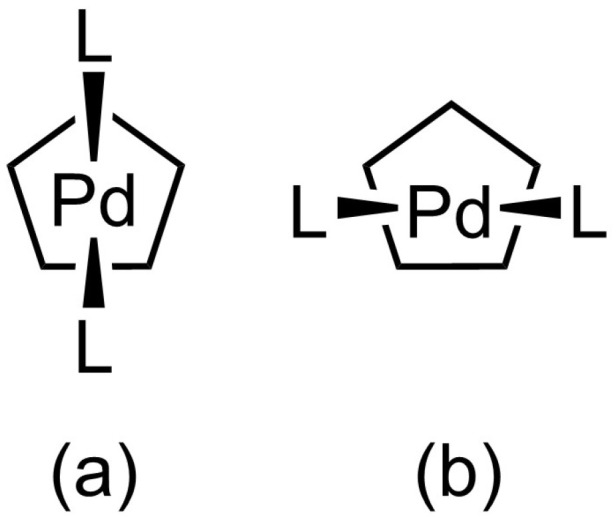
Conformations of ML_2_ planes with respect to cyclopentadienyl rings in CpML_2_ complexes: (**a**) the eclipsed conformation; (**b**) the staggered conformation.

**Figure 1:**
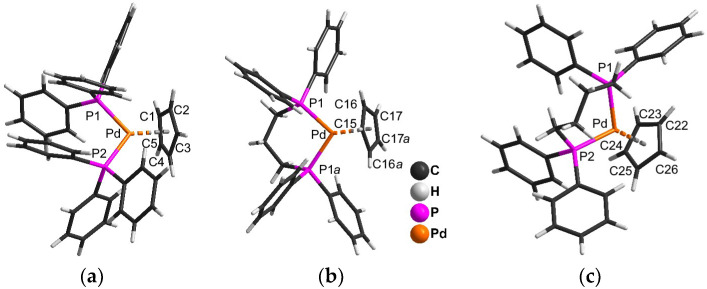
Molecular structure of **1**∙C_6_H_5_CH_3_ (**a**), **7**∙MeCN (**b**), and **8**∙C_6_H_5_CH_3_ (**c**) with atoms represented by displacement ellipsoids of 50% probability. Anion [BF_4_]^−^, solvent molecules, and hydrogen atoms are omitted for clarity. Details of the structure refinement are given in Table S1. Selected bond distances (Å) and angles (°): **1**: Pd1–P1 = 2.2880(6), Pd1–P2 = 2.2685(6), Pd1–C2 = 2.268(2), Pd1–C1 = 2.372(2), Pd1–C5 = 2.330(2), Pd1–C4 = 2.268(2), Pd1–C3 = 2.329(2), ∠P1–Pd–P2 = 97.87(2); **7**: Pd1–P1(a) = 2.244, Pd1–C17(a) = 2.317, Pd1–C16 = 2.254, Pd1–C15 = 2.312, ∠P1–Pd–P1*a* = 94.99; **8**: Pd1–P1 = 2.263(1), Pd1–P2 = 2.258(1), Pd1–C22 = 2.271(4), Pd1–C26 = 2.335(3), Pd1–C25 = 2.270(4), Pd1–C24 = 2.349(4), Pd1–C23 = 2.346(4), ∠P1–Pd–P2 = 96.12(4).

Single crystals of compounds **1**, **7**, and **8** suitable for X-ray crystallography were obtained via slow diffusion of toluene into acetonitrile solutions of the complexes. Figure 1 shows the molecular structures of **1**, **7**, and **8**. Selected interatomic distances and angles are given in the figure captions. In all cases, the asymmetric unit of the crystals contains the complex cation, the [BF_4_]^−^ anion, and a solvent molecule. The molecular structures of these complexes reveal that the planes containing the metal and the two-ligand phosphorus atoms are almost perpendicular (88.05°, 89.33°, 88.05° for **1**, **7**, and **8**, respectively) to the Cp ring planes. The alignments of the ML_2_ planes relative to the Cp rings are typically described as “eclipsed” or “staggered” conformations [27,34], which differ from each other due to a rotation of the cyclopentadienyl ring around the metal-ring centroid vector (Scheme 4). Our study of the [PdCp(PPh_3_)_2_]^+^ cation **1** (Figure 1) reveals that it adopts eclipsed conformation. In the molecular structure of complex **1**, the cyclopentadienyl C–C bond lengths radiating from C2 (1.447(3) and 1.417(3) Å) are long, and the bond lengths of the C3–C4–C5–C1 sequence show characteristic [27,34] butadiene-like bond length alternation (1.399(3), 1.454(3), and 1.382(4) Å). The variations in the Pd–C distances are also typical of the pattern expected for an eclipse conformation; thus, the Pd–C2 distance of 2.268(2) Å is much shorter than the Pd1–C1 and Pd1–C3 distances (2.372(2) and 2.329(2) Å, respectively). Structural results for compounds **7** and **8** reveal that cations [PdCp(dppp)]^+^ and [PdCp(dppb)]^+^ adopt a geometry that is closer to staggered than eclipsed. For instance, in complex **8,** the structural features include three shorter-than-average bond distances (C26–C25 = 1.407(8), C24–C23 = 1.370(8), and C22–C26 = 1.407(7) Å) and two longer bond lengths (C25–C24 = 1.436(7) and C23–C22 = 1.428(7) Å). In addition, Pd1–C22 and Pd1–C25 are shorter than the other metal–carbon bonds (Figure 1). The Pd–P distances and P–Pd–P bond angles in complex **7** (Pd1–P1(a) = 2.244 Å, ∠P1–Pd–P1a = 94.99°) are in good agreement with the corresponding values in complex **8** (Pd1–P1 = 2.263(1) Å, Pd1–P2 = 2.258(1) Å, ∠P1–Pd–P2 = 96.12(4)°), as well as with those previously reported for cationic cyclopentadienyl palladium-diphosphine complexes [26,27,34].

**Figure 2:**
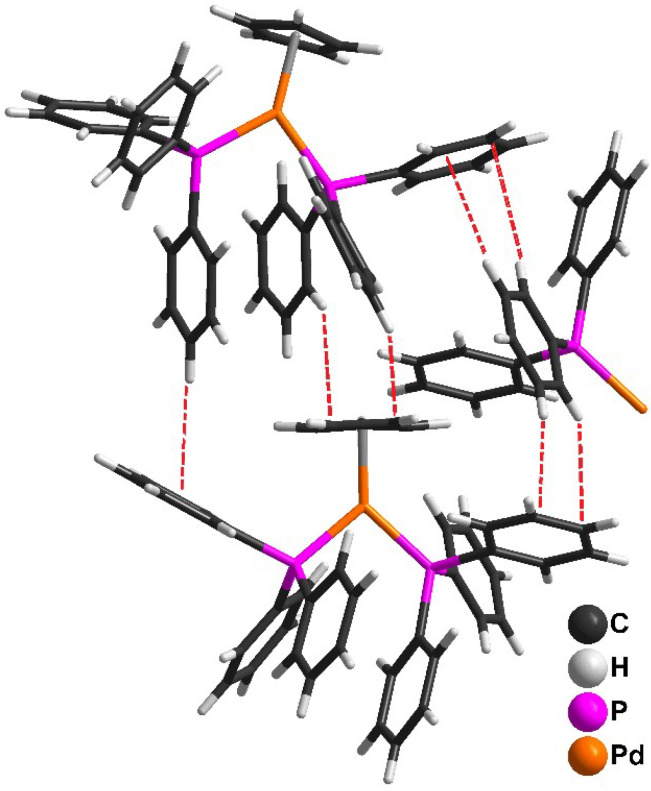
C–H…π interactions in the crystal packing of **1**.

**Figure 3:**
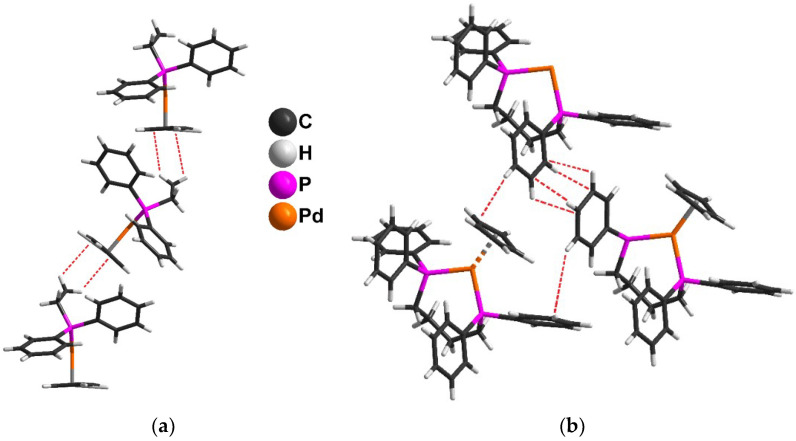
C–H…π interactions in the crystal packing of complexes **7** (**a**) and **8** (**b**).

In the crystal packing of all presented complexes C–H…π and π-π contacts play one of the main structure-directed roles. Cp^–^ and Ph-groups give a lot of possibilities to realize a whole potential of this kind interactions. While all presented structures are different due to the difference in the contact types provided by the geometry of coordinated phosphines. This aspect plays the most important role in the crystal packing formation for the presented complexes. In the case of PPh_3_ (**1**) intramolecular π-π interactions bound Ph-groups of the adjacent phosphine ligands. Intermolecular contacts are of C–H…π nature and involved Ph-groups and Cp^–^ ligands of adjacent molecules (Figure 2). In the crystal structure of complex **7**, the situation changes dramatically due to the presence of the (CH_2_)_3_ linker in the diphosphine ligand. We found strong CH_2_…π intermolecular contact-directed formation of 1D supramolecular structures (Figure 3a) with suppression of π–π interactions. On the other hand, complex **8** has practically the same (CH_2_)_4_ linker (one CH_2_-group longer) which is practically inactive in the formation of intermolecular interactions. Otherwise π–π and C–H…π contacts strongly direct orientation of Ph-groups of the diphosphine ligands (Figure 3b). This fact can be of electronic nature caused by the linker elongation.

Moreover, H…F contacts between cationic Pd complexes and BF_4_^–^ anions also present in all presented structures. Some crystal packing projections are summarized in the Supporting Information (Figures S1–S3, SI).

**Figure 4:**
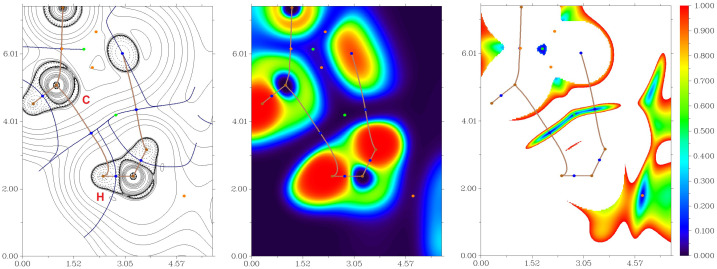
Contour line diagram of the Laplacian of electron density distribution ∇^2^ρ(**r**), bond paths, and selected zero-flux surfaces (**left panel**), visualization of electron localization function (ELF, **center panel**), and reduced density gradient (RDG, **right panel**) analyses for C–H⋯π interactions in the X-ray structure **7**. Bond critical points (3, −1) are shown in blue, nuclear critical points (3, −3)—in pale brown, ring critical points (3, +1)—in orange, and cage critical points (3, +3)—in light green. Bond paths are shown as pale brown lines, length units are in Å, and the color scales for the ELF and RDG maps are presented in a.u.

In order to confirm or disprove the hypothesis on the existence of intermolecular interactions C–H⋯π in the X-ray structures **1**, **7**, and **8** and approximately quantify the strength of these supramolecular contacts from a theoretical viewpoint, DFT calculations followed by a topological analysis of the electron density distribution using the QTAIM approach [58] were carried out (see Computational Details and Table S2 in Supporting Information). Results of QTAIM analysis are summarized in Table 3. The contour line diagram of the Laplacian of electron density distribution ∇^2^ρ(**r**), bond paths, and selected zero-flux surfaces as well as a visualization of electron localization function (ELF) and reduced density gradient (RDG) analyses for intermolecular interactions C–H⋯π in the X-ray structure **7** are shown in Figure 4.

QTAIM analysis of model structures demonstrates the presence of bond critical points (3, −1) for intermolecular interactions C–H⋯π in the X-ray structures **1**, **7**, and **8** (Table 3). The low magnitude of the electron density (0.002–0.009 a.u.), positive values of the Laplacian of electron density (0.008–0.031 a.u.), and zero or very close to zero positive energy density (0.000–0.002 a.u.) in these bond critical points (3, −1) and estimated strength of appropriate short contacts (0.3–1.6 kcal/mol) are typical for very weak noncovalent interactions involving π-systems [59,60,61,62,63,64,65,66,67]. The balance between the Lagrangian kinetic energy *G*(**r**) and potential energy density *V*(**r**) at the bond critical points (3, −1) (the ratio –*G*(**r**)/*V*(**r**) ≥1) reveals that a covalent contribution in all intermolecular interactions C–H⋯π in the X-ray structures **1**, **7**, and **8** is absent [68] (Table 3). The Laplacian of electron density is typically decomposed into the sum of contributions along the three principal axes of maximal variation, giving the three eigenvalues of the Hessian matrix (λ_1_, λ_2_ and λ_3_), and the sign of λ_2_ can be utilized to distinguish bonding (attractive, λ_2_ < 0) weak interactions from non-bonding ones (repulsive, λ_2_ > 0) [69,70]. Thus, intermolecular interactions C–H⋯π in the X-ray structures **1**, **7**, and **8** are attractive (Table 3).

**Table 3:** Values of the density of all electrons—ρ(**r**), Laplacian of electron density—∇^2^ρ(**r**) and appropriate λ_2_ eigenvalues, energy density—*H*_b_, potential energy density—*V*(**r**), and Lagrangian kinetic energy—G(**r**) (a.u.) at the bond critical points (3, −1), corresponding to intermolecular interactions C–H⋯π in the X-ray structures **1**, **7**, and **8**, and estimated strength for these contacts *E*_int_ (kcal/mol).

Length of Intermolecular Contact in Å *	ρ(r)	∇^2^ρ(r)	λ_2_	*H*_b_	*V*(r)	*G*(r)	*E*_int_ **
**1**							
2.686	0.008	0.030	−0.008	0.002	−0.004	0.006	1.3
2.881	0.006	0.020	−0.006	0.001	−0.003	0.004	0.9
2.799	0.006	0.018	−0.006	0.001	−0.002	0.003	0.6
2.961	0.004	0.015	−0.004	0.001	−0.002	0.003	0.6
3.322	0.002	0.008	−0.002	0.000	−0.001	0.001	0.3
2.986	0.004	0.016	−0.004	0.001	−0.002	0.003	0.6
3.053	0.004	0.015	−0.004	0.001	−0.002	0.003	0.6
**7**							
3.034	0.004	0.013	−0.004	0.001	−0.001	0.002	0.3
2.966	0.004	0.015	−0.004	0.001	−0.002	0.003	0.6
3.077	0.004	0.013	−0.004	0.001	−0.001	0.002	0.3
3.034	0.004	0.013	−0.004	0.001	−0.001	0.002	0.3
3.077	0.004	0.013	−0.004	0.001	−0.001	0.002	0.3
3.028	0.005	0.017	−0.005	0.001	−0.002	0.003	0.6
**8**							
2.778	0.006	0.023	−0.006	0.001	−0.003	0.004	0.9
2.877	0.006	0.019	−0.006	0.001	−0.002	0.003	0.6
2.662	0.009	0.031	−0.009	0.001	−0.005	0.006	1.6
2.952	0.005	0.019	−0.005	0.002	−0.002	0.004	0.6
3.043	0.003	0.011	−0.003	0.001	−0.001	0.002	0.3

* The “classic” Bondi’s [71] van der Waals radii for H and C atoms are 1.20 and 1.70 Å, respectively, and “modern” values of van der Waals radii suggested by Alvarez [72] H and C atoms are 1.20 and 1.77 Å, respectively. ** *E*_int_ ≈ −*V*(**r**)/2 [73].

### 2.3. Catalytic Studies

**Scheme 5:**
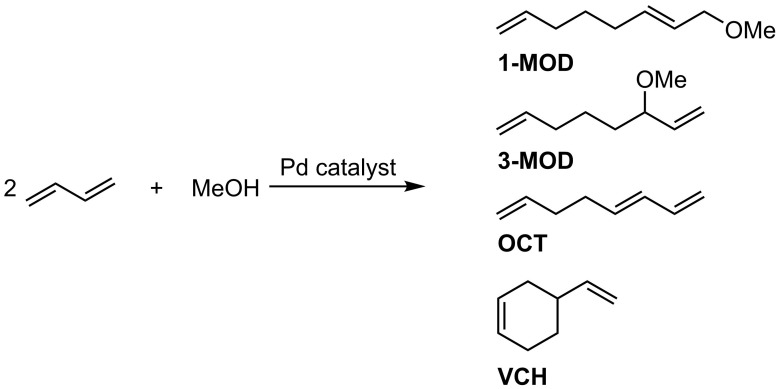
Telomerization of 1,3-butadiene with methanol.

The cationic palladium complexes **1**–**10** (Scheme 2) were tested in the telomerization of 1,3-butadiene with MeOH (Scheme 5). Palladium-catalyzed telomerization, which involves the linear dimerization of 1,3-dienes with the simultaneous addition of a nucleophile, is an efficient organic transformation that complies with most green chemistry principles [74,75]. The telomerization of 1,3-butadiene (BD) with methanol to produce 1-methoxy-2,7-octadiene as the main product is a commercial route (developed by Dow Chemical) to synthesize 1-octene [76]. The selectivity of the reaction depends on the nature of the nucleophiles and the ligands coordinated to the reactive palladium center (Scheme 5) [74,75,77,78,79]. Previous studies have shown that several palladium complexes with monodentate phosphines[51,80,81,82,83,84,85], diphosphines [86], and NHC (NHC—N-heterocyclic carbene) ligands [87,88,89] are effective catalysts for the telomerization of BD with methanol.

As shown in Table 4, the selectivity and BD conversion largely depended on the nature of the catalyst. The reaction was performed in the presence of an excess of nucleophiles relative to the diene ([MeOH]_0_:[BD]_0_ = 1), so turnover number (*TON*) is based on butadiene conversion. As one can see from Table 4, for phosphine-ligated complexes, *TON* decreased in the following order: **1**, **2** > **3** > **5** > **4** ≫ **6**–**10**. For triarylphosphines as ligands, this correlates with the decreasing basicity of the phosphine ligand (cf. [90]), except TOMPP, which is more basic than PPh_3_ [91]. However, the ease of oxidation of the phosphine ligand also increases with its basicity, and therefore, the loss of productivity of the catalyst could be explained by a larger loss of the phosphine ligand through oxidation during the catalysis as proposed by van Leeuwen et al. [76]. Complexes **6**–**10** with diphosphines were not suitable for this telomerization reaction. It is known that diphosphines often perform worse than monophosphine catalysts as ligands for the telomerization of dienes with alcohols [76,86]. Next, the most interesting pre-catalyst **1** was studied with lower palladium concentration. When only 0.0021 mol% of Pd loading is used, higher turnovers are obtained (23,700) with practically the same selectivity as in entry 1. It should be noted that at 90 °C, the conversion of butadiene and the selectivity decrease (62% vs. 82% 1-MOD selectivity at 90 °C and 70 °C, respectively), showing that the catalyst formed from compound **1** is unstable at high temperatures. Finally, with a [BD]_0_/[Pd]_0_ ratio of 120,000:1 the conversion of BD as well as selectivity substantially dropped, and some amounts of vinylcyclohexene as a result of the Diels–Alder reaction were detected. For compound **1,** the catalyst selectivities obtained are similar to those reported by van Leeuwen et al. [84], who used a somewhat similar catalyst system Pd^0^(dvd)_2_/2PPh_3_/5NaOMe (dvd—tetramethyldivinyldisiloxane), or by Beller et al. [80], who utilized conventional Pd(OAc)_2_/3PPh_3_/100NEt_3_, albeit with TON being 30% lower in our case (23,500 vs. 30,000–34,000 reported in [80,84]). Notably, these conversions and selectivities were achieved under solventless conditions and without the use of any added base.

For compound **1,** the obtained catalyst *TON*s and selectivity are as might be expected from results by Beller et al. using catalyst system Pd(OAc)_2_/3PPh_3_.

**Scheme 6:**
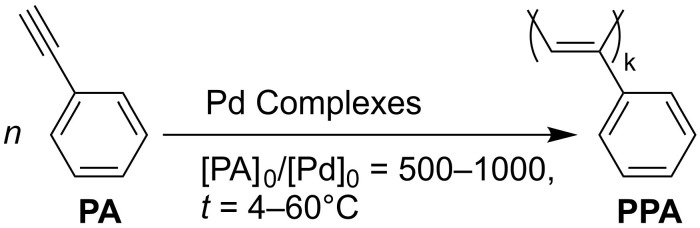
Polymerization of phenylacetylene with Pd catalysts **1**–**10**.

At this point, we decided to test the catalytic activity of compounds **1**–**10** towards another type of substrate, and the addition polymerization of phenylacetylene (**PA**) was examined (Scheme 6, Table 5). Polyacetylenes are used in applications such as photonics, light-emitting diodes, conductors or semi-conductors, sensors, gas separation membranes, and chiral materials [92,93]. Poly(phenylacetylene) (**PPA**) is mainly produced from catalyzed reactions of phenylacetylene with early- and late-transition metal complexes, driven either via metathesis or insertion polymerization mechanisms [92]. Rhodium-based late-transition metal catalysts are commonly used, but they are associated with a high-cost disadvantage [94,95,96]. Therefore, there is a need to develop other catalytic systems, with Pd-based catalysts attracting most attention [92,93,97,98,99,100]. It has been established [92,93,97] that efficient palladium catalysts for the polymerization of phenylacetylene also fall under the category of cationic organometallic compounds bearing bulky phosphine ligands. The most notable examples are [(dppf)Pd(MeCN)(CH_3_)]OTf [101], developed by Darkwa, Pollack et al., and [(tBuXPhos)Pd(Me)](BAr^f^) bearing a Buchwald-type dialkylbiarylphosphine ligand, proposed by Chen, Daugulis, and Brookhart [93]. Therefore, we expected that compounds **1**–**10** would exhibit interesting catalytic properties during screening.

As shown in Table 5, the polymer yields and molecular weights largely depended on the nature of the catalyst. Typically, low-molecular-weight powdery products were obtained. However, cationic pre-catalyst **3** with a bulky TOMPP ligand produced high-molecular-weight polyphenylacetylene with significant product yield. This is consistent with the known hypothesis that sterically bulky phosphine [93,97,99,103,104] (including TOMPP) or NHC-carbene [105,106] ligands are crucial for catalyst activity in the polymerization of substituted acetylenes. Compared with known Pd(II) catalysts [93,97,99,103,104], catalyst **3,** described here, showed moderate to high activity.

In order to achieve more stable reaction conditions using catalyst **3**, we investigated the polymerization of PA while varying temperature, ratio of [PA]_0_:[Pd]_0_, reaction time, and solvent. Polymerization temperature had a significant effect on catalytic activity and the polydispersity index (PDI) of the resulting polymers. As temperature (entries 11–14, Table 5) increased, yield and activity were enhanced; however, molecular weight and selectivity decreased significantly. Increasing the temperature above 80 °C led to significantly decreased productivity resulting from catalyst deactivation. The thermal stability of compound **3** in the polymerization of phenylacetylene, therefore, was modest. Gel permeation chromatography curves (Figures S95–S100, SI) of the obtained PPAs at 25–50 °C displayed bimodal distribution, which is characteristic of Pd initiators [93,99,101,107,108]. The effect of reaction time on conversion, activity, and molecular weight is summarized in the entries in Table 5. The polymerization was quenched at the respective time using acidified methanol. As shown, conversion increased with time, and approximately the same molecular weight was observed independent of conversion. The PDI and *M*_n_ were larger than expected for living polymerization based on monomer weight, which may indicate that catalyst **3** exhibits slow initiation. Polymerization proceeded in all solvents tested (see entries 22–27, Table 5). However, polymerization was substantially slower in acetonitrile, presumably due to its strong coordination to the palladium center, which inhibits coordination with the monomer. The reaction in THF afforded optimal results in terms of molecular weight (*M*_w_ = 23.2 kDa), isolated yield (87%), and high *TON* (435). The PPA samples obtained with catalyst **3** showed a sharp absorption peak in the infrared spectra at 740 cm^−1^ and a broad peak at 890 cm^−1^ (Figure S101, SI) characteristic of *cis*-PPA [102]. The ^1^H NMR spectra of the polymers also show a sharp singlet at δ 5.86 (s, 1H) due to the vinylic protons in the polymer, and a set of broad peaks at δ 6.72 (m, 2H) and δ 6.98 (m, 3H) ppm, which are associated with a head-to-tail structure of a *cis*-transoidal PPA (Figure S102, SI) [94,101]. Regarding the formation of the active species with such catalysts, we suggest that the reaction involves the transformation of the η^5^-cyclopentadienyl ligand to the η^1^-Cp-isomer. In the next step, insertion of the coordinated PA into the Pd–C bond occurs. Therefore, the ^1^H NMR spectra of PPA show the presence of a weak peak at 3.6 ppm, indicative of the Cp fragment from the cyclopentadienyl end group in the obtained polyphenylacetylene samples (Figure S102, SI).

## 3. Materials and Methods

### 3.1. General Procedures and Materials

All air- and/or moisture-sensitive compounds were manipulated using standard high-vacuum line, Schlenk, or cannula techniques under an argon atmosphere. Argon (Arnika-Prom-Service, Angarsk, Russia) was purified before feeding to the reactor via passing through columns packed with oxygen scavenger and molecular sieve 4A (Aldrich, Steinheim, Germany), respectively. Diethyl ether (99%), petroleum ether, THF (99.9%), and toluene (99.5%) (ZAO Vekton, Saint Petersburg, Russia) were distilled from sodium−benzophenone. CH_2_Cl_2_ (99.5%) (ZAO Vekton), CH_3_CN (99.9%) (ZAO Vekton, Saint Petersburg, Russia), phenylacetylene (98%) (ZAO Vekton, Saint Petersburg, Russia), and methanol (99%) (ZAO Vekton, Saint Petersburg, Russia) were distilled from CaH_2_. Solvents were stored over molecular sieves. Cyclopentadiene was freshly prepared through a thermal retro-Diels–Alder reaction from commercially obtained dicyclopentadiene (95%) (Acros Organics, Geel, Belgium). Other chemicals were purchased from Acros Organics, Sigma-Aldrich, and ABCR and employed without drying or any further purification. All glassware was dried for at least 2 h in a 150 °C oven and cooled under an argon atmosphere. Pd(acac)_2_ was synthesized according to a procedure from the literature [109] and recrystallized from acetone. The cationic acetylacetonate complexes were prepared according to known procedures from the literature [38,50,51,52,53,54]. All other reagents were obtained commercially and used as received. NMR spectra were recorded on a Bruker DPX-400 (400 MHz) spectrometer (at room temperature) (Karlsruhe, Germany) and Oxford X-Pulse benchtop (60 MHz) spectrometer (at 40 °C) (Abingdon, Oxon, UK). IR spectra were recorded on a Simex Infralum FT 801 spectrometer (Novosibirsk, Russia). Ultraviolet−visible light (UV-vis) spectra were recorded on an OKB Spectr SF-2000 spectrophotometer (Saint Petersburg, Russia). GC-FID and GC–MS analyses were performed on a Chromatec Crystall 5000.2 (Yoshkar-Ola, Russia) (SGE BPX-5 capillary column, Ringwood, Australia, internal standard benzene) and Shimadzu QP2010 Ultra (Kyoto, Japan) (GSBP-5MS capillary column, Delaware City, DE, USA), respectively. The molecular weights and polydispersity of the polymers were estimated in THF using a Thermo-Dionex HPLC system (Sunnyvale, CA, USA) (Phenomonex, Phenogel Linear column, Torrance, CA, USA). A set of monodisperse polystyrene standards (Agilent, Shropshire, UK) covering the molecular weight range of 10^3^ to 10^6^ was used.

### 3.2. General Procedure for 1,3-Butadiene Telomerization

All the reactions were carried out in a custom-made stainless-steel autoclave with a volume of 20 mL. The 1,3-butadiene (39 mmol) was first condensed into the reactor and its volume was controlled. The catalyst was then added directly into the reactor pot as a solution in CH_2_Cl_2_. After that, methanol (39 mmol) was added to the reactor via a syringe. The reactor was then closed and placed in an oil bath at a temperature of 70 °C. The reaction was stirred magnetically at 700 rpm for 2 h. The reaction was stopped using an ice bath. The catalytic reactions were analyzed using GC-FID, and the response factor for the telomers was determined using the pure fraction of telomers obtained (identified via GC–MS) after vacuum distillation of the reaction mixture.

### 3.3. General Procedure for Phenylacetylene Polymerization

All polymerizations were conducted in a 5 mL glass reactor equipped with a magnetic stirrer under an Ar atmosphere in an oil bath. The Pd catalyst was stirred in 1 mL of solvent, after which 1 mL of PA (9.1 mmol) was added. The reaction mixture was then stirred for the designated time, and subsequently poured into a large amount of acidified methanol to precipitate the polymer as a yellow powder. The product was isolated via filtration and dried under vacuum to reach a constant weight. If necessary, the polymer was purified through dissolving it in THF and precipitating it with methanol to obtain a fine yellow powder. The polymer samples were characterized using ^1^H NMR, FT-IR, and GPC analysis. PPA with high *cis*-content: ^1^H NMR (CDCl_3_): δ 6.97 (m, 3H), 6.73 (m, 2H), 5.86 (s, 1H).

### 3.4. Synthesis of Palladium Complexes

#### 3.4.1. Preparation of (η^5^-Cyclopentadienyl)bis(triphenylphosphine-κP)palladium(II) Tetrafluoroborate, [Pd(η^5^-C_5_H_5_)(PPh_3_)_2_]BF_4_ (**1**)

[Pd(acac)(PPh_3_)_2_]BF_4_ (0.400 g, 0.490 mmol) was dissolved in 10 mL of MeOH, and BF3∙OEt2 (0.30 mL, 2.45 mmol) was added to this solution. The obtained yellow solution was stirred for 1.0 h at room temperature, and then cooled to 0 °C. To the resulting reaction mixture, cyclopentadiene (0.22 mL, 2.45 mmol) was added and stirred for 8.0 h at room temperature. The resulting reaction mixture was concentrated to 5 mL under vacuum. Addition of diethyl ether (20 mL) formed a purple precipitate, which was collected, washed with diethyl ether (2 × 10 mL), and dried (8 h) under vacuum to afford complex **1** as a purple powder (327 mg, 85.3%). Anal. Calcd for C_41_H_35_BF_4_P_2_Pd: C, 62.90; H, 4.51. Found: C, 63.03; H, 4.44. ESI-MS (positive ion mode, MeCN): *m*/*z* 695.12 [M^+^]. ^1^H NMR (400 MHz, CDCl_3_, 25 °C): δ 7.45–7.38 (m, 6H, H_Ph_), 7.38–7.28 (m, 24H, H_Ph_), 5.51 (t, *J* = 2.1 Hz, 5H, H_Cp_).^13^C{^1^H} NMR (101 MHz, CDCl_3_, 25 °C): δ 133.73 (observed as virtual triplets (vt) due to virtual coupling [56,110], *J* = 6.1 Hz, C_Ph,ortho_), 131.57 (s, C_Ph,para_), 130.65 (vt, ^1^*J*(P,C_ipso_) = 24.5 Hz), 128.97 (vt, *J* = 5.6 Hz, C_Ph,meta_), 104.27 (t, *J* = 2.3 Hz, C_Cp_). ^13^C{^1^H, gated decoupling mode} NMR (101 MHz, CDCl_3_, 25 °C): δ 133.73 (d, *J*_CH_ = 163 Hz, C_Ph,ortho_), 131.57 (d, *J*_CH_ = 163 Hz, C_Ph,para_), the signals for *ipso*-C of PPh_3_ were not clearly observed, 128.97 (d, *J*_CH_ = 165 Hz, C_Ph,meta_), 104.27 (d, *J*_CH_ = 178 Hz, C_Cp_). ^31^P{^1^H} NMR (162 MHz, CDCl_3_, 25 °C): δ 33.77 (s). ^19^F NMR (376 MHz, CDCl_3_, 25 °C): δ −153.58, −153.64 (intensity ratio of approximately 20:80 corresponding to the natural abundances of ^10^B and ^11^B, respectively). IR (KBr, $\tilde{\nu }$, cm^−1^): 3115, 3075 (sh), 3055 [ν(C–H from CH, Ph and Cp)]; 1585, 1573, 1481, 1436 [ν_as_(C=C, Ph) and phenyl ring deformations]; 1398, 1352 [ν_as_(C=C, Cp) and cyclopentadienyl ring deformations]; 1312, 1284, 1185, 1162, 1122 δ(C–H, Ph and Cp, plane); 1084 δ(C–H, Ph and Cp, plane); 1098(sh), 1062, 1035(sh) ν_as_(B–F); 1026, 999, 928, 831, 752 δ(C–H, Ph and Cp, off-plane); 795 [partially resolved in the IR bands of the symmetric ν_s_(B–F) vibrations, which appear in the spectrum due to violation of the symmetry of the environment]; 703, 695 ν(P–C_Ar_, Ph); 617 (rings deformations). UV-vis (C_2_H_4_Cl_2_, λ_max_/nm, (ε_max_/L mol^−1^ cm^−1^)): 350 (15,660).

#### 3.4.2. Preparation of (η^5^-Cyclopentadienyl)bis[tris(4-methylphenyl)phosphine-κP]palladium(II) Tetrafluoroborate, [Pd(η^5^-C_5_H_5_)(P(p-MeC_6_H_4_)_3_)_2_]BF_4_ (**2**)

##### Preparation of **2** in CH_2_Cl_2_ as Solvent

[Pd(acac)(P(*p*-MeC_6_H_4_)_3_)_2_]BF_4_ (0.150 g, 0.167 mmol) was dissolved in 5 mL of CH_2_Cl_2_, and to this solution was added 10% *v*/*v* solution of BF_3_∙OEt_2_ in CH_2_Cl_2_ (0.23 mL, 0.183 mmol). The obtained yellow solution was stirred for 0.5 h at room temperature, and then cooled to 0 °C. To the resulting reaction mixture was added cyclopentadiene (0.08 mL, 0.832 mmol) and stirred for 1.0 h at room temperature. The resulting reaction mixture was concentrated to ~3 mL under vacuum. Addition of diethyl ether (10 mL) formed a purple precipitate, which was collected, washed with diethyl ether (2 × 10 mL), dissolved in MeCN (5 mL), filtered and dried (8 h) under vacuum to afford complex **2** as a purple powder (104 mg, 71.5%). Anal. Calcd for C_47_H_47_BF_4_P_2_Pd: C, 65.11; H, 5.46. Found: C, 65,22; H, 5,58. ESI-MS (positive ion mode, MeCN): *m*/*z* 779.22 [M^+^]. ^1^H NMR (400 MHz, CD_3_CN, 25 °C): δ 7.25–7.16 (m, 12H, H_Ph_), 7.16–7.08 (m, 12H, H_Ph_), 5.49 (t, *J* = 2.1 Hz, 5H, H_Cp_), 2.35 (s, 18H, C*H*_3_). ^13^C{^1^H} NMR (101 MHz, CD_3_CN, 25 °C): δ 142.06 (s, C_Ph,para_),133.67 (vt, *J* = 6.3 Hz, C_Ph,ortho_), 129.24 (vt, *J* = 5.8 Hz, C_Ph,meta_), 127.79 (vt, ^1^*J*(P,C_ipso_) = 26.5 Hz), 103.71 (t, *J* = 1.9 Hz, C_Cp_), 20.35 (s, *C*H_3_).^31^P{^1^H} NMR (162 MHz, CD_3_CN, 25 °C): δ 32.89 (s). IR (KBr, $\tilde{\nu }$, cm^−1^): 3110, 3042, 3023 [ν(C–H from CH, Ph and Cp)]; 1597, 1541, 1497, 1448 [ν_as_(C=C, Ph) and phenyl ring deformations]; 1398, 1354 [ν_as_(C=C, Cp) and cyclopentadienyl ring deformations]; 1310, 1281, 1211, 1268, 1191, 1016 δ(C–H, Ph and Cp, plane); 1094, 1055, 1035 ν_as_(B–F); 831, 804, 761 δ(C–H, Ph and Cp, off-plane); 795 [partially resolved in the IR bands of the symmetric ν_s_(B–F) vibrations, which appear in the spectrum due to violation of the symmetry of the environment]; 710 ν(P–C_Ar_, Ph); 615 (rings deformations). UV-vis (C_2_H_4_Cl_2_, λ_max_/nm, (ε_max_/L mol^−1^ cm^−1^)): 353 (16,200).

##### Preparation of **2** in MeOH as Solvent

[Pd(acac)(P(*p*-MeC_6_H_4_)_3_)_2_]BF_4_ (0.150 g, 0.167 mmol) was dissolved in 10 mL of MeOH, and BF3∙OEt2 (0.10 mL, 0.84 mmol) was added to this solution. The obtained yellow solution was stirred for 0.25 h at room temperature, and then cooled to 0 °C. To the resulting reaction mixture, cyclopentadiene (0.14 mL, 1.67 mmol) was added and stirred for 8.0 h at room temperature. The resulting reaction mixture was concentrated to ~3 mL under vacuum. Addition of diethyl ether (10 mL) formed a purple precipitate, which was collected, washed with diethyl ether (2 × 10 mL), and dried (8 h) under vacuum to afford complex **2** as a purple powder (120 mg, 82.5%). Anal. Calcd for C_47_H_47_BF_4_P_2_Pd: C, 65.11; H, 5.46. Found: C, 65.25; H, 5.53. ^1^H NMR (60 MHz, CD_3_CN, 40 °C): δ 7.27–7.09 (m, 24H, H_Ph_), 5.50 (t, *J* = 2.1 Hz, 5H, H_Cp_), 2.36 (s, 18H, C*H*_3_).

#### 3.4.3. Preparation of (η^5^-Cyclopentadienyl)bis[tris(2-methoxyphenyl)phosphine-κP]palladium(II) Tetrafluoroborate, [Pd(η^5^-C_5_H_5_)(TOMPP)_2_]BF_4_ (TOMPP = Tris(2-methoxyphenyl)phosphine) (**3**)

[Pd(acac)(TOMPP)_2_]BF_4_ (0.338 g, 0.339 mmol) was dissolved in 10 mL of MeOH, and BF_3_∙OEt_2_ (0.20 mL, 1.70 mmol) was added to this solution. The obtained yellow solution was stirred for 0.25 h at room temperature, and then cooled to 0 °C. Cyclopentadiene (0.14 mL, 1.70 mmol) was added to the resulting reaction mixture and stirred for 2.0 h at room temperature. Addition of diethyl ether formed a green precipitate, which was collected, washed with diethyl ether (2 × 10 mL), and dried (8 h) under vacuum to afford complex **3** as a green powder (231 mg, 71.0%). Anal. Calcd for C_47_H_47_BF_4_O_6_P_2_Pd: C, 58.62; H, 4.92. Found: C, 59.09; H, 5.15. ESI-MS (positive ion mode, MeCN): *m*/*z* 875.19 [M^+^]. ^1^H NMR (400 MHz, CD_3_CN, 25 °C): δ 7.61–7.05 (m, 12H, H_Ph_), 7.02–6.73 (m, 12H, H_Ph_), 5.12 (t, *J* = 2.2 Hz, 5H, H_Cp_), 3.75–2.84 (m, br., 18H, C*H*_3_O). ^13^C{^1^H} NMR (101 MHz, CD_3_CN, 25 °C): δ 159.99 (s, C_Ph,2-OMe_), 132.34 (s, C_Ph_), 119.99 (d, ^1^*J*(P,C_ipso_) =11.5 Hz), 111.64 (s, C_Ph_), 105.38 (t, br., *J* = 2.2 Hz, C_Cp_), 54.73 (s, *C*H_3_O). ^31^P{^1^H} NMR (162 MHz, CDCl_3_, 25 °C): δ 8.22 (br., Δν(FWHM) = 365 Hz). IR (KBr, $\tilde{\nu }$, cm^−1^): 3064, 3011 (sh) [ν(C–H from CH, Ar and Cp)]; 1586, 1574, 1518, 1457, 1431 [ν_as_(C=C, Ph) and aryl ring deformations]; 1396, 1372 [ν_as_(C=C, Cp) and cyclopentadienyl ring deformations]; 1277, 1134 δ(C–H, Ar and Cp, plane); 1249, 1165 (ν_Ar-O-C_); 1180 (δ_C–H_ from O-CH_3_); 1071, 1056, 1018 ν_as_(B–F); 960, 943, 904, 850, 828, 798, 754 δ(C–H, Ar and Cp, off-plane); 795 (partially resolved in the IR bands of the symmetric ν_s_(B–F) vibrations, which appear in the spectrum due to violation of the symmetry of the environment); 686, 668 ν(P–C_Ar_, Ph); 617 (rings deformations). UV-vis (C_2_H_4_Cl_2_, λ_max_/nm, (ε_max_/L mol^−1^ cm^−1^)): 283(20,700), 370 (11,300).

#### 3.4.4. Preparation of (η^5^-Cyclopentadienyl)bis[tris(2-furyl)phosphine-κP]palladium(II) Tetrafluoroborate, [Pd(η^5^-C_5_H_5_)(TFP)_2_]BF_4_ (TFP = Tris(2-furyl)phosphine) (**4**)

[Pd(acac)(TFP)_2_]BF_4_ (0.173 g, 0.229 mmol) was dissolved in a mixture of 5 mL of MeOH and 2 mL of CH_2_Cl_2_, and BF_3_∙OEt_2_ (0.14 mL, 1.15 mmol) was added to this solution. The obtained yellow solution was stirred for 0.5 h at room temperature, and then cooled to 0 °C. Cyclopentadiene (0.1 mL, 1.15 mmol) was added to the resulting reaction mixture and stirred for 2.0 h at room temperature. The resulting reaction mixture was concentrated to ~3 mL under vacuum. Addition of *n*-pentane formed a brown precipitate, which was collected, washed with diethyl ether (2 × 10 mL), and dried (8 h) under vacuum to afford complex **4** as a brown powder (123 mg, 74.1%). Anal. Calcd for C_29_H_23_BF_4_O_6_P_2_Pd: C, 48.20; H, 3.21. Found: C, 47.37; H, 3.15. ESI-MS (positive ion mode, MeCN): *m*/*z* 635.00 [M^+^]. ^1^H NMR (400 MHz, CD_3_CN, 25 °C): δ 7.79–7.74 (m, 6H, H_Fur5_) (Fur–furyl ring), 6.84–6.78 (m, 6H, H_Fur3_), 6.58–6.48 (m, 6H, H_Fur4_), 6.04 (t, *J* = 2.3 Hz, 5H, H_Cp_). ^13^C NMR (101 MHz, CD_3_CN, 25 °C): δ 151.10 (vt, *J* = 3.3 Hz, C_Fur5_), 141.95 (vt, ^1^*J*(P,C) = 44.6 Hz C_Fur2_), 124.35 (vt, *J* = 10.2 Hz, C_Fur3_), 111.90 (vt, *J* = 4.1 Hz, C_Fur4_), 102.68 (t, *J* = 2.3 Hz, C_Cp_). ^31^P{^1^H} NMR (162 MHz, CD_3_CN, 25 °C): δ −23.99 (s). IR (KBr, $\tilde{\nu }$, cm^−1^): 3151, 3132, 3089 ν(C–H from Fur and Cp); 1549, 1456, 1368 ν(C=C from Fur) and ring deformations; 1396 (ν(C=C, Cp) and cyclopentadienyl ring deformations); 1284, 1216, 1163, δ(C–H from Fur and Cp, plane) and ring deformations; 1127, 1059, 1034(sh) ν_as_(B–F); 1010 ring deformations and δ(C–H from Fur, plane); 908, 882, 830, 757, 669, 631 δ(C–H, Fur and Cp, off-plane). UV-vis (C_2_H_4_Cl_2_, λ_max_/nm, (ε_max_/L mol^−1^ cm^−1^)): 349 (14,400).

#### 3.4.5. Preparation of (η^5^-Cyclopentadienyl)bis[tris(2-thienyl)phosphine-κP]palladium(II) Tetrafluoroborate, [Pd(η^5^-C_5_H_5_)(TTP)_2_]BF_4_ (TTP = Tris(2-thienyl)phosphine) (**5**)

[Pd(acac)(TTP)_2_]BF_4_ (0.132 g, 0.154 mmol) was dissolved in 7 mL of MeOH, and cyclopentadiene (0.08 mL, 0.8 mmol) was added to this solution. The obtained solution was stirred for 0.5 h at room temperature, and then cooled to 0 °C. ∙OEt_2_ (0.1 mL, 0.772 mmol) was added to the resulting reaction mixture and stirred for 72 h at room temperature. The resulting brown precipitate was filtered, washed with diethyl ether (2 × 10 mL), and dried (8 h) under vacuum to afford complex **5** as a purple powder (21 mg, 16.5%). ESI-MS (positive ion mode, MeCN): *m*/*z* 732.86 [M^+^]. ^1^H NMR (400 MHz, CD_3_CN, 25 °C): δ 7.88–7.82 (m, 6H, H_Thi5_) (Thi–thienyl ring), 7.38–7.32 (m, 6H, H_Thi3_), 7.14 (t, *J* = 4.3 Hz, 6H, H_Thi4_), 5.80 (t, *J* = 2.2 Hz, 5H, H_Cp_). ^13^C NMR (101 MHz, CD_3_CN, 25 °C): δ 139.51 (vt, *J* = 7.2 Hz, C_Thi5_), 136.51 (vt, *J* = 2.5 Hz, C_Thi4_), 134.13 (vt, *J* = 29.9 Hz, C_Thi2_), 129.85 (vt, *J* = 6.9 Hz, C_Thi3_), 105.27 (t, *J* = 2.4 Hz, C_Cp_). ^31^P{^1^H} NMR (162 MHz, CD_3_CN, 25 °C): δ −1.84 (s). Due to lack of sample EA, FTIR and UV-vis spectra were not obtained.

#### 3.4.6. Preparation of (η^5^-Cyclopentadienyl)(1,1′-bis(diphenylphosphino)ferrocene-κ^2^P,P′)palladium(II) Tetrafluoroborate, [Pd(η^5^-C_5_H_5_)(dppf)]BF_4_ (Dppf = 1,1′-Bis(diphenylphosphino)ferrocene) (**6**)

[Pd(acac)(dppf)]BF_4_ (0.300 g, 0.354 mmol) was dissolved in 10 mL of MeOH and BF_3_∙OEt_2_ (0.22 mL, 1.77 mmol) was added to this solution. The obtained red solution was stirred for 0.25 h at room temperature, and then cooled to 0 °C. Cyclopentadiene (0.15 mL, 1.77 mmol) was added to the resulting reaction mixture and stirred for 8.0 h at room temperature. The resulting reaction mixture was concentrated to ~3 mL under vacuum. Addition of Et_2_O formed precipitate, which was collected, washed with diethyl ether (2 × 10 mL), and dried (8 h) under vacuum to afford complex 6 as a dark red powder (180 mg, 62.0%). Anal. Calcd for C_39_H_33_BF_4_FeP_2_Pd: C, 57.64; H, 4.09. Found: C, 57.05; H, 4.27. ESI-MS (positive ion mode, MeCN): *m*/*z* 525.04 [M^+^]. ^1^H NMR (400 MHz, CD_3_CN, 25 °C): δ 7.70–7.60 (m, 12H, H_Ph_), 7.59–7.49 (m, 8H, H_Ph,meta_), 5.45 (t, J = 2.1 Hz, 5H, H_Cp_), 4.59–4.52 (m, 4H, H_Cp′(dppf),β_), 4.39–4.34 (m, 4H, H_Cp′(dppf),α_). ^13^C NMR (101 MHz, CD_3_CN, 25 °C): δ 134.04 (vt, *J* = 24.7 Hz, C_Ph,ipso_), 132.94 (vt, *J* = 6.7 Hz, C_Ph,ortho_), 131.49 (s, C_Ph,para_), 128.66 (vt, *J* = 5.7 Hz, C_Ph,meta_), 102.86 (s, C_Cp_), 76.46 (vt, *J* = 5.8 Hz, C_Cp′(dppf),α_), 74.12 (vt, *J* = 3.7 Hz, C_Cp′(dppf),β_), 72.43 (vt, *J* = 33.0 Hz, C_Cp′(dppf),ipso_). ^31^P{^1^H} NMR (162 MHz, CD_3_CN, 25 °C): δ 38.51 (s). IR (KBr, $\tilde{\nu }$, cm^−1^): 3117, 3100, 3050 (ν(C–H from CH, Ph and Cp)); 1480, 1436 (ν_as_(C=C, Ph) and phenyl ring deformations); 1389, 1355 (ν_as_(C=C, Cp) and cyclopentadienyl ring deformations); 1307, 1282, 1182, 1174, 1167 δ(C–H, Ph and Cp, plane); 1082, 1058, 1037 ν_as_(B–F); 829, 800, 752, δ(C–H, Ph and Cp, off-plane); 696 ν(P–C_Ar_, Ph); 631 (rings deformations). UV-vis (C_2_H_4_Cl_2_, λ_max_/nm, (ε_max_/L mol^−1^ cm^−1^)): 341 (9000).

#### 3.4.7. Preparation of (η^5^-Cyclopentadienyl)(1,3-bis(diphenylphosphino)propane-κ^2^P,P′)palladium(II) Tetrafluoroborate, [Pd(η^5^-C_5_H_5_)(Dppp)]BF_4_ (Dppp = 1,3-Bis(diphenylphosphino)propane) (**7**)

[Pd(acac)(dppp)]BF_4_ (0.100 g, 0.142 mmol) was dissolved in mixture of 5 mL of MeOH and 2 mL of CH_2_Cl_2_, and BF_3_∙OEt_2_ (0.09 mL, 0.709 mmol) was added to this solution. The obtained yellow solution was stirred for 0.5 h at room temperature, and then cooled to 0 °C. Cyclopentadiene (0.06 mL, 0.709 mmol) was added to the resulting reaction mixture and stirred for 2.0 h at room temperature. The resulting reaction mixture was concentrated to ~3 mL under vacuum. Addition of *n*-pentane formed a pink precipitate, which was collected, washed with diethyl ether (2 × 10 mL), and dried (8 h) under vacuum to afford complex **7** as a pink powder (71 mg, 74.2%). Anal. Calcd for C_32_H_31_BF_4_P_2_Pd: C, 57.30; H, 4.66. Found: C, 56.95; H, 4.78. ESI-MS (positive ion mode, MeCN): *m*/*z* 583.09 [M^+^]. ^1^H NMR (400 MHz, CD_3_CN, 25 °C): δ 7.61–7.42 (m, 20H, H_Ph_), 5.51 (t, *J* = 2.1 Hz, 5H, H_Cp_), 2.78–2.57 (m, C*H*_2_, 4H), 2.17 (s, C*H*_2_, overlapped with impurities in CD_3_CN). ^13^C NMR (101 MHz, CD_3_CN, 25 °C): δ 132.11 (vt, *J* = 5.9 Hz, C_Ph,ortho_), 131.49 (vt, *J* = 25.0 Hz, C_Ph,ipso_), 131.19 (s, C_Ph,para_), 128.67 (vt, *J* = 5.6 Hz, C_Ph,meta_), 100.86 (t, *J* = 1.9 Hz, C_Cp_), 24.08 (vt, *J* = 19.8 Hz, *C*H_2_), 17.92 (s, *C*H_2_). ^31^P{^1^H} NMR (162 MHz, CD_3_CN, 25 °C): δ 16.65 (s). IR (KBr, $\tilde{\nu }$, cm^−1^): 3107, 3086, 3055 (ν(C–H from CH, Ph and Cp)); 2933, 2910, 2862 (ν(C–H from CH_2_)); 1586, 1573, 1452, 1483, 1436 (ν_as_(C=C, Ph) and phenyl ring deformations); 1400, 1346 (ν_as_(C=C, Cp) and cyclopentadienyl ring deformations); 1309, 1280, 1187, 1155, 1122(sh), 1100 δ(C–H, Ph and Cp, plane); 1082, 1059, 1036 ν_as_(B–F); 999, 971, 932 δ(C–H, Ph and Cp, off-plane); 911, 874, 857 δ(C–H from CH_2_, rocking); 831 δ(C–H, Ph and Cp, off-plane); 791 partially resolved in the IR bands of the symmetric ν_s_(B–F) vibrations, which appear in the spectrum due to violation of the symmetry of the environment; 751 δ(C–H, Ph and Cp, off-plane); 698, 673 ν(P–C); 617 (rings deformations). UV-vis (C_2_H_4_Cl_2_, λ_max_/nm, (ε_max_/L mol^−1^ cm^−1^)): 329 (15,500).

#### 3.4.8. Preparation of (η^5^-Cyclopentadienyl)(1,4-bis(diphenylphosphino)butane-κ^2^P,P′)palladium(II) Tetrafluoroborate, [Pd(η^5^-C_5_H_5_)(Dppb)]BF_4_ (Dppb = 1,4-Bis(diphenylphosphino)butane) (**8**)

[Pd(acac)(dppb)]BF_4_ (0.100 g, 0.139 mmol) was dissolved in 5 mL of MeOH, and BF_3_∙OEt_2_ (0.09 mL, 0.70 mmol) was added to this solution. The obtained yellow solution was stirred for 0.5 h at room temperature, and then cooled to 0 °C. Cyclopentadiene (0.06 mL, 0.70 mmol) was added to the resulting reaction mixture and stirred for 2.0 h at room temperature. The resulting reaction mixture was concentrated to ~3 mL under vacuum. Addition of diethyl ether formed a purple precipitate, which was collected, washed with diethyl ether (2 × 10 mL), and dried (8 h) under vacuum to afford complex **8** as a purple powder (60 mg, 60.0%). Anal. Calcd for C_33_H_33_BF_4_P_2_Pd: C, 57.88; H, 4.86. Found: C, 57.36; H, 4.96. ESI-MS (positive ion mode, MeCN): *m*/*z* 597.11 [M^+^]. ^1^H NMR (400 MHz, CD_3_CN, 25 °C): δ 7.68–7.49 (m, 20H, H_Ph_), 5.39 (t, *J* = 2.1 Hz, 5H, H_Cp_), 2.60 (s, br, 4H, C*H*_2_), 1.83–1.63 (m, 4H, C*H*_2_). ^13^C NMR (101 MHz, CD_3_CN, 25 °C): δ 133.98 (vt, *J* = 24.5 Hz, C_Ph,ipso_), 132.51 (vt, *J* = 5.8 Hz, C_Ph,ortho_), 131.59 (s, C_Ph,para_), 129.15 (vt, *J* = 5.4 Hz, C_Ph,meta_), 102.33 (t, *J* = 1.9 Hz, C_Cp_), 27.40 (vt, *J* = 16.4 Hz, *C*H_2_), 22.99 (s, *C*H_2_). ^31^P{^1^H} NMR (162 MHz, CD_3_CN, 25 °C): δ 33.06 (s). IR (KBr, $\tilde{\nu }$, cm^−1^): 3111, 3086, 3056 ν(C–H from CH, Ph and Cp); 2940, 2923, 2864 ν(C–H from CH_2_); 1586, 1573, 1483, 1452 (sh.), 1436 (ν_as_(C=C, Ph) and phenyl ring deformations); 1399, 1352 (ν_as_(C=C, Cp) and cyclopentadienyl ring deformations); 1308, 1281, 1232, 1188, 1163 δ(C–H, Ph and Cp, plane); 1099, 1055, 1036 ν_as_(B–F); 1000 δ(C–H, Ph and Cp, off-plane); 895, 877 δ(C–H from CH_2_, rocking); 832 δ(C–H, Ph and Cp, off-plane); 793 (partially resolved in the IR bands of the symmetric ν_s_(B–F) vibrations, which appear in the spectrum due to violation of the symmetry of the environment); 746 δ(C–H, Ph and Cp, off-plane); 698, 664 ν(P–C); 617 (rings deformations). UV-vis (C_2_H_4_Cl_2_, λ_max_/nm, (ε_max_/L mol^−1^ cm^−1^)): 334 (13,900).

#### 3.4.9. Preparation of (η^5^-Cyclopentadienyl)(1,5-bis(diphenylphosphino)pentane-κ^2^P,P′)palladium(II) Tetrafluoroborate, [Pd(η^5^-C_5_H_5_)(Dpppt)]BF_4_ (Dpppt = 1,5-Bis(diphenylphosphino)pentane) (**9**)

[Pd(acac)(dpppt)]BF_4_ (0.100 g, 0.136 mmol) was dissolved in 6 mL of MeOH, and BF_3_∙OEt_2_ (0.08 mL, 0.68 mmol) was added to this solution. The obtained yellow solution was stirred for 0.5 h at room temperature, and then cooled to 0 °C. Cyclopentadiene (0.06 mL, 0.70 mmol) was added to the resulting reaction mixture and stirred for 2.0 h at room temperature. Addition of diethyl ether formed a purple precipitate, which was collected, washed with diethyl ether (2 × 10 mL), and dried (8 h) under vacuum to afford complex **9** as a purple powder (45 mg, 48.0%). Anal. Calcd for C_34_H_35_BF_4_P_2_Pd: C, 58.44; H, 5.05. Found: C, 59.96; H, 4.95. ESI-MS (positive ion mode, MeCN): *m*/*z* 611.12 [M^+^] the obtained isotopic pattern can be interpreted as [M_1_]^+^:[M_2_]^2+^:[M_3_]^3+^ = 9.5:0.1:0.4. ^1^H NMR (400 MHz, CD_3_CN, 25 °C): δ 7.61–7.26 (m, 20H, H_Ph_), 5.79 (t, *J* = 2.0 Hz, 0.3H, isomer (dimer), H_Cp_), 5.72 (t, *J* = 2.0 Hz, 0.7H, isomer (trimer), H_Cp_), 5.44 (t, *J* = 2.0 Hz, 4H, major isomer (monomer), H_Cp_), 2.54 (tt, *J* = 9.6, 4.8 Hz, 4H, C*H*_2_), 2.30–2.10 (m, 2H, C*H*_2_, overlapped with impurities in CD_3_CN) 1.84–1.69 (m, 4H, C*H*_2_). ^13^C NMR (101 MHz, CD_3_CN, 25 °C): δ 133.57 (vt, *J* = 23.7 Hz, C_Ph,ipso_), 132.35 (vt, *J* = 5.7 Hz, C_Ph,ortho_), 131.34 (s, C_Ph,para_), 129.03 (vt, J = 5.4 Hz, C_Ph,meta_), 103.32 (t, *J* = 2.0 Hz, major isomer, C_Cp_), 103.02 (s, br, minor isomer, C_Cp_), 26.74 (vt, *J* = 15.8 Hz, P–*C*H_2_), 22.56 (t, *J* = 5.9 Hz, *C*H_2_), 22.16 (s, *C*H_2_). ^13^C{^1^H, gated decoupling mode} NMR (101 MHz, CD_3_CN, 25 °C): δ 132.35 (d, *J*_CH_ = 161 Hz, 8C, C_Ph,ortho_), 131.34 (d, *J*_CH_ = 163 Hz, 4C, C_Ph,para_), 129.03 (d, *J*_CH_ = 164 Hz, 8C, C_Ph,meta_), the signals for *ipso*-C of PPh_2_-moiety were not observed, 103.32 (dt, *J*_CH_ = 177 Hz, *J* = 6 Hz, 5C, C_Cp_), 26.74, (td, *J*_CH_ = 125 Hz, *J* = 16 Hz, 2C, *C*H_2_), 22.56, (t, *J*_CH_ = 125 Hz, 1C, *C*H_2_), 22.16 (t, *J*_CH_ = 127 Hz, 2C, *C*H_2_). ^31^P{^1^H} NMR (162 MHz, CD_3_CN, 25 °C): δ 28.76 (s, 0.04P), 28.25 (s, 0.16P), 19.08 (s, 0.8P). IR (KBr, $\tilde{\nu }$, cm^−1^): 3107, 3057 ν(C–H from CH, Ph and Cp); 2932, 2863 ν(C–H from CH_2_); 1586, 1572, 1483, 1453, 1436 (ν_as_(C=C, Ph) and phenyl ring deformations); 1403, 1352 (ν_as_(C=C, Cp) and cyclopentadienyl ring deformations); 1311, 1282, 1187, 1163 δ(C–H, Ph and Cp, plane); 1097, 1056, 1036 ν_as_(B–F); 999 δ(C–H, Ph and Cp, off-plane); 928, 894 δ(C–H from CH_2_, rocking); 830, 804 δ(C–H, Ph and Cp, off-plane); 792 (partially resolved in the IR bands of the symmetric ν_s_(B–F) vibrations, which appear in the spectrum due to violation of the symmetry of the environment); 745 δ(C–H, Ph and Cp, off-plane); 697, 652ν(P–C); 616(rings deformations). UV-vis (C_2_H_4_Cl_2_, λ_max_/nm, (ε_max_/L mol^−1^ cm^−1^)): 339 (11,800).

#### 3.4.10. Preparation of {[Pd(η^5^-C_5_H_5_)(Dpphx)]BF_4_}_n_ (Dpphx = 1,6-Bis(diphenylphosphino)hexane, n = 2, 3) (**10**) (Mixture of di-μ-(1,6-Bis(diphenylphosphino)hexane-κ^2^P,P′)-bis[(η^5^-cyclopentadienyl)palladium(II)] Bis(tetrafluoroborate) and Tri-μ-(1,6-Bis(diphenylphosphino)hexane-κ^2^P,P′)-tris[(η^5^-cyclopentadienyl)palladium(II)] Tris(tetrafluoroborate))

[Pd(acac)(dpphx)]_n_[BF_4_]_n_ (0.227 g, 0.304 mmol) (*n* = 2, as previously reported [52]) was dissolved 10 mL of MeOH, and BF_3_∙OEt_2_ (0.19 mL, 1.52 mmol) was added to this solution. The obtained yellow solution was stirred for 0.5 h at room temperature, and then cooled to 0 °C. Cyclopentadiene (0.14 mL, 1.52 mmol) was added to the resulting reaction mixture and stirred for 2.0 h at room temperature. Addition of diethyl ether/*n*-pentane mixture formed a purple precipitate, which was collected, washed with diethyl ether (2 × 10 mL), and dried (8 h) under vacuum to afford complex **10** as a purple powder (178 mg, 82.0%). The product contained Et_2_O as adduct ([Et_2_O]:[Pd] ≈ 1:3) via ^1^H NMR analysis. Anal. Calcd for C_35_H_37_BF_4_P_2_Pd: C, 58.97; H, 5.23. Found: C, 59.25; H, 5.48. ESI-MS (positive ion mode, MeCN): *m***/***z* 625.14 [M^+^], the obtained isotopic pattern can be interpreted as [M_1_]^+^:[M_2_]^2+^:[M_3_]^3+^ = 0:4:6. ^1^H NMR (400 MHz, CD_3_CN, 25 °C): δ 7.72–7.21 (m, 20H, H_Ph_), 5.78 (t, *J* = 2.0 Hz, 3H, major isomer (trimer), H_Cp_), 5.76–5.69 (m, 1.5H, minor isomer (dimer), H_Cp_), 5.61 (t, *J* = 2.0 Hz, 0.5H, minor isomer, H_Cp_), 2.34–2.06 (m, 3H, C*H*_2_), 1.99–1.46 (m, 3H, C*H*_2_, overlapped with impurities in CD_3_CN), 1.41–1.21 (m, 6H, C*H*_2_). ^13^C NMR (101 MHz, CD_3_CN, 25 °C): δ (the signals for *ipso*-C of PPh_2_-moiety were not observed) 131.93 (vt, *J* = 5.6 Hz, C_Ph,ortho_), 130.57 (s, C_Ph,para_), 128.11 (s, J C_Ph,meta_), 102.18 (s, minor isomer (dimer), C_Cp_), 101.51 (s, major isomer (trimer), C_Cp_), 29.16 (s, *C*H_2_), 25.46 (s, *C*H_2_). ^31^P{^1^H} NMR (162 MHz, CD_3_CN, 25 °C): δ 28.61 (s, 0.6P), 28.39–28.22 (m, 0.3P), 27.11(s, 0.1P). IR (KBr, $\tilde{\nu }$, cm^−1^): 3107, 3055 [ν(C–H from CH, Ph and Cp)]; 2933, 2864 (ν(C–H from CH_2_)); 1483, 1458, 1436 (ν_as_(C=C, Ph) and phenyl ring deformations); 1401, 1352 (ν_as_(C=C, Cp) and cyclopentadienyl ring deformations); 1312, 1281, 1186, 1161 δ(C–H, Ph and Cp, plane); 1099, 1056, 1036 ν_as_(B–F); 998 δ(C–H, Ph and Cp, off-plane); 929 δ(C–H from CH_2_, rocking); 831 δ(C–H, Ph and Cp, off-plane); 790 (partially resolved in the IR bands of the symmetric ν_s_(B–F) vibrations, which appear in the spectrum due to violation of the symmetry of the environment); 745 δ(C–H, Ph and Cp, off-plane); 697 ν(P–C); 616(rings deformations). UV-vis (C_2_H_4_Cl_2_, λ_max_/nm, (ε_max_/L mol^−1^ cm^−1^)): 333 (15,000).

### 3.5. Reaction of [Pd(κ^2^-O,O′-Acac)(TOMPP)_2_]BF_4_ with BF_3_∙OEt_2_

A solution of [Pd(*κ*^2^-*O*,*O*′-acac)(TOMPP)_2_]BF_4_ (100 mg), 5 eq. of MeCN, and 2 eq. of BF_3_∙OEt_2_ in 5 mL of CH_2_Cl_2_ was stirred at room temperature for 2 h. The resulting yellow solution was concentrated under vacuum and a yellow-orange precipitate was obtained, which was collected and dried under vacuum. Yield: 85 mg. No chemical analyses were completed. ESI-MS (positive ion mode, MeCN): *m*/*z* 1023.13. ^1^H NMR (400 MHz, CDCl_3_, 25 °C): 7.70–7.40 (m, 6H, H_Ar*i*_), 7.30–7.10 (m, 6H, H_Ar*i*_), 7.10–6.65 (m, 12H, H_Ar*i*_), 6.00 (s, 1H, C*H*(*κ*^1^-C-acac)), 3.81 (s, 15H, H_OMe_) 3.70–2.10 (m, 3H, H_OMe_), 2.30 (s, 6H, C*H*_3_(*κ*^1^-C-acac)), 2.02(s, 2.5H, C*H*_3_(MeCN)).^13^C{^1^H} NMR (101 MHz, CDCl_3_, 25 °C) δ 192.50 (s, C=O), 160.21 (s, C_Ar2_), 135.97 (s, C_Ar6_), 134.52 (s, C_Ar4_), 122.56 (s (br.), C_Ar5_,), 113.36 (br., C_Ar3_), 112.20 (d (br.), ^1^*J*(P,C_Ar1_) =67.1 Hz, C_Ar1_), 101.96 (d, ^2^*J*(P,C) = 3.9 Hz), *C*H(*κ*^1^-C-acac)), 58.76 (s, C_OMe_), 24.21 (s, *C*H_3_(*κ*^1^-C-acac)), 2.07 (s, MeCN). ^31^P{^1^H} NMR (162 MHz, CDCl_3_, 25 °C): δ 32.72 (s). ^19^F NMR (376 MHz, CDCl_3_, 25 °C): δ {−138.58 (0.7F), −138.64 (2.3F); 3F, BF_3_∙L}, {−152.85 (1.1F), −152.90 (2.9F); 4F, BF_4_} (intensity ratio ≈ 20:80 corresponds to the natural abundances of ^10^B and ^11^B, respectively). ^11^B NMR (128 MHz, CDCl_3_, 25 °C): δ 0.56 (1B, BF_3_∙L), −1.07 (1B, BF_4_).

### 3.6. X-ray Crystallographic Studies

Crystallographic data and refinement details are given in Table S1. The diffraction data for complexes **1**, **7**, and **8** were collected on a Bruker D8 Venture diffractometer with a CMOS PHOTON III detector and IµS 3.0 source (MoKα radiation, λ = 0.71073 Å) at 150 K. The φ- and ω-scan techniques were employed. Absorption correction was applied using SADABS (Bruker Apex3 software suite: Apex3, SADABS-2016/2 and SAINT, version 2018.7-2; Bruker AXS Inc.: Madison, WI, 2017). Structures were solved using SHELXT [111] and refined via full-matrix least-squares treatment against |F|^2^ in anisotropic approximation with SHELX 2014/7 [112] in the ShelXle program [113]. H-atoms were refined in geometrically calculated positions.

CCDC 2258771, 2258772, and 2258773 contain the supplementary crystallographic data for compounds **1**, **7**, and **8**. These data can be obtained free of charge via http://www.ccdc.cam.ac.uk/conts/retrieving.html accessed on 12 May 2023, or from the Cambridge Crystallographic Data Centre, 12 Union Road, Cambridge CB2 1EZ, UK; fax: (+44) 1223-336-033; or e-mail: deposit@ccdc.cam.ac.uk.

### 3.7. Computational Details

The single-point calculations based on the experimental X-ray geometries of compounds **1**, **7**, and **8** have been carried out at the DFT level of theory (ωB97XD/CEP-121G) with the help of the Gaussian-09 [114] program package. The topological analysis of the electron density distribution has been performed using the Multiwfn program (version 3.7) [115]. The Cartesian atomic coordinates for model structures are presented in Table S2.

## 4. Conclusions

We have demonstrated that cationic η^5^-cyclopentadienyl palladium complexes with tertiary phosphorus donor ligands can be prepared through reacting cationic acetylacetonate complexes with cyclopentadiene in the presence of BF_3_∙OEt_2_. Ten new arylphosphine-ligated cationic palladium cyclopentadienyl complexes [Pd(Cp)(L)_n_]_m_[BF_4_]_m_ (*n* = 2, *m* = 1: L = PPh_3_, P(*p*-Tol)_3_, *tris*(*ortho*-methoxyphenyl)phosphine, *tri*-2-furylphosphine, *tri*-2-thienylphosphine; *n* = 1, *m* = 1: L = dppf, dppp, dppb, 1,5-bis(diphenylphosphino)pentane; *n* = 1, *m* = 2 or 3: 1,6-bis(diphenylphosphino)hexane) were synthesized and characterized. Three palladium(II) complexes (**1**, **7**, and **8**) have been structurally characterized using X-ray crystallography. In the molecular structures of complexes **1**, **7**, and **8**, the Cp ring is nearly perpendicular to the PdP_2_ plane. In the cation of complex **1**, the cyclopentadienyl ring adopts a nearly eclipsed orientation, while in complexes **7** and **8**, it adopts a staggered orientation with respect to the perpendicular PdP_2_ fragment. Inspection of the crystal structures, as well as DFT calculations using QTAIM analysis, made it possible to recognize (Cp^−^)⋯(Ph-group) and (Cp^−^)⋯(CH_2_-group) non-covalent interactions, which are of C–H…π nature. The catalytic potential of [Pd(Cp)(L)_2_][BF_4_] (L—monodentate phosphine ligands) was demonstrated in the industrially important telomerization of 1,3–butadiene with methanol. Complexes containing PPh_3_ or P(*p*-Tol)_3_ catalyzed the formation of methoxyocta-2,7-dienes with good yields. The other studied palladium complexes showed substantially lower activities. In the presence of 0.0021 mol% palladium loading, the desired telomers were obtained with a chemoselectivity of 82% and *TON* of up to 2.4∙10^4^ mol BD per mol Pd. We have also demonstrated that complex [Pd(Cp)(TOMPP)_2_]BF_4_ is an efficient catalyst for the polymerization of phenylacetylene. Specifically, catalyst activities of (1.1–8.9) × 10^3^ g_PA_·(mol_Pd_·h)^−1^ were observed in neat monomer or THF as solvent, and obtained PPA featured >90% of *cis* double bond content.

## Figures and Tables

**Table 1:** ^31^P{^1^H} chemical shifts of the cationic phosphine-Pd-Cp complexes and coordination-induced shifts Δ for the complexes.

Free Ligand	δ ^31^P{^1^H} Chemical Shift, ppm	Complex	δ ^31^P{^1^H} Chemical Shift, ppm	Δ(^31^P{^1^H}) (δ_complex_ − δ_ligand_), ppm
PPh_3_	−6.3 [44]	**1**	33.8	40.1
P(*p*-Tol)_3_	−8.3 [44]	**2**	32.9	41.2
TOMPP	−37.9 [45]	**3**	8.2	46.1
TFP	−75.2 [46]	**4**	−24.0	51.2
TTP	−45.6 [47]	**5**	−1.8	43.8
dppf	−16.8 [48]	**6**	38.6	55.4
dppp	−17.7 [49]	**7**	16.7	34.4
dppb	−16.3 [49]	**8**	33.1	49.4
dppent	−16.5 [49]	**9**	19.1	35.6
dpphex	−16.4 [49]	10	28.6	45.0

**Table 2:** Selected ^1^H chemical shift values (δ, ppm) for the cationic palladium complexes (400 MHz CD_3_CN, 25 °C).

Complex	Cp	Phosphine Ligand
**1** *^a^*	5.51 (t, *J* = 2.1 Hz, 5H)	7.45–7.38 (m, 6H, H_Ph_), 7.38 –7.28 (m, 24H, H_Ph_)
**2**	5.49 (t, *J* = 2.1 Hz, 5H)	7.25–7.16 (m, 12H, H_Ph_), 7.16–7.08 (m, 12H, H_Ph_), 2.35 (s, 18H, C*H*_3_)
**3**	5.12 (t, *J* = 2.2 Hz, 5H)	7.61–7.05 (m, 12H, H_Ph_), 7.02–6.73 (m, 12H, H_Ph_), 3.75–2.84 (m, br., 18H, C*H*_3_O)
**4**	6.04 (t, *J* = 2.3 Hz, 5H)	7.79–7.74 (m, 6H, H_Fur5_) (Fur = furyl), 6.84–6.78 (m, 6H, H_Fur3_), 6.58–6.48 (m, 6H, H_Fur4_)
**5**	5.80 (t, *J* = 2.2 Hz, 5H)	7.88–7.82 (m, 6H, H_Thi5_) (Thi = thienyl), 7.38–7.32 (m, 6H, H_Thi3_), 7.14 (t, *J* = 4.3 Γц, 6H, H_Thi4_)
**6**	5.45 (t, J = 2.1 Hz, 5H)	7.70–7.60 (m, 12H, H_Ph_), 7.59–7.49 (m, 8H, H_Ph,meta_), 4.59–4.52 (m, 4H, H_Cp′(dppf),β_), 4.39–4.34 (m, 4H, H_Cp′(dppf),α_).
**7**	5.51 (t, *J* = 2.1 Hz, 5H)	7.61–7.42 (m, 20H, H_Ph_), 2.78–2.57 (m, C*H*_2_, 4H), 2.17 (s, C*H*_2_).
**8**	5.39 (t, *J* = 2.1 Hz, 5H)	7.68–7.49 (m, 20H, H_Ph_), 2.60 (s, br, 4H, C*H*_2_), 1.83–1.63 (m, 4H, C*H*_2_).
**9**	5.72 (t, *J* = 2.0 Hz, 0.7H, minor isomer), 5.44 (t, *J* = 2.0 Hz, 4H, major isomer),	7.61–7.26 (m, 20H, H_Ph_), 2.54 (tt, *J* = 9.6, 4.8 Γц, 4H, C*H*_2_), 2.30–2.10 (m, 2H, C*H*_2_) 1.84–1.69 (m, 4H, C*H*_2_).
**10**	5.78 (t, *J* = 2.0 Hz, 3H, major isomer), 5.76–5.69 (m, 1.5H, minor isomer),	7.72–7.21 (m, 20H, H_Ph_), 2.34–2.06 (m, 3H, C*H*_2_), 1.99–1.46 (m, 3H, C*H*_2_), 1.41–1.21 (m, 6H, C*H*_2_).

*^a^* 400 MHz, CDCl_3_, 25 °C.

**Table 4:** Influence of the nature of catalysts **1**–**10** on conversion and selectivity in telomerization of 1,3-butadiene with methanol.

Entry ^[a]^	Pd	Conv. BD % ^[b]^	Selectivity, mol%			Chemo,mol% ^[c]^	*n*/*iso* ^[d]^	*E*/*Z* ^[e]^	*TON* ^[f]^
			OCT	3-MOD	1-MOD				
1	**1**	67.4	13.5	5.0	81.5	86.5	16	10	8100
4	**2**	66.8	13.0	5.6	81.5	87.1	15	10	8000
5	**3**	25.9	4.7	3.3	92.0	95.3	28	18	3100
6	**4**	8.7	4.8	4.4	90.8	95.2	21	12	1050
7	**5**	18.0	5.0	4.9	90.1	95.0	18	10	2150
8	**6**	0.3	—	—	—	—	—	—	—
9	**7**	0.2	—	—	—	—	—	—	—
10	**8**	0.3	—	—	—	—	—	—	—
11	**9**	0.3	—	—	—	—	—	—	—
12	**10**	0.2	—	—	—	—	—	—	—
13 ^[g]^	**1**	49.2	12.8	4.9	81.5	86.4	17	12	11,800
14 ^[h]^	**1**	49.3	16.6	4.5	77.8	82.3	17	11	23,700
15 ^[i]^	**1**	3.5	11.4	4.5	69.9	74.4	15	10	4200

^[a]^ Reaction conditions: *t* = 70 °C, 0.0083 mol% of Pd, [BD]_0_/[Pd]_0_ = 12,000, [MeOH]_0_/[Bd]_0_ = 1, *n*_Pd_ = 3.25 μmol, reaction time—2 h. ^[b]^ 1,3-Butadiene conversion. ^[c]^ Chemoselectivity = (1-MOD + 3-MOD)(1-MOD + 3-MOD + OCT + VCH)^−1^. ^[d]^ Regioselectivity = (1-MOD)(3-MOD)^−1^. ^[e]^ Stereoselectivity = (*trans*-1-MOD)(*cis*-1-MOD)^−1^. ^[f]^ In units of (mol of BD) (mol of Pd)^−1^. ^[g]^ *n*_Pd_ = 1.63 μmol, [BD]_0_/[Pd]_0_ = 24,000. ^[h]^ *n*_Pd_ = 1.63 μmol, [BD]_0_/[Pd]_0_ = 48,000, reaction time—5 h. ^[i]^ *n*_Pd_ = 0.33 μmol, [BD]_0_/[Pd]_0_ = 120,000, reaction time—8 h.

**Table 5:** Polymerization of PA with Pd catalysts **1**–**10**.

Entry ^[a]^	Complex	Time (h)	Solvent	t (°C)	Yield (%)	*TON* ^[b]^	Activity ^[c]^	Sel ^[d]^ (%)	Cis ^[e]^ (%)	Polymer	
										*M*_w_ (/10^3^) ^[f]^	*M*_w_/ *M*_n_^[f]^
1	**1**	24	—	25	0.3	3	13	63	90	2.6	1.6
2	**2**	24	—	25	0.2	2	9	65	94	2.2	1.5
3	**3**	24	—	25	26.2	262	1113	78	94	17.2	2.1
4	**4**	24	—	25	0	0	0	—		—	—
5	**5**	24	—	25	0	0	0	—		—	—
6	**6**	24	—	25	1.3	13	55	92	92	19.0	2.1
7	**7**	24	—	25	0	0	0	—		—	—
8	**8**	24	—	25	0	0	0	—		—	—
9	**9**	24	—	25	0	0	0	—		—	—
10	**10**	24	—	25	0	0	0	—		—	—
11 ^[h]^	**3**	24	—	4	2.7	14	60	>99	90	21.4	2.1
12 ^[h]^	**3**	24	—	25	53.4	267	1140	79	94	15.5	2.0
13 ^[h]^	**3**	24	—	50	91.3	457	1940	47	98	7.9	1.6
14 ^[h]^	**3**	24	—	60	84.8	424	1800	38	98	2.0	1.5
15 ^[h]^	**3**	1	—	25	2.2	11	1219	99	n.d.	16.5	2.1
16 ^[h]^	**3**	2	—	25	5.2	26	1320	99	94	17.9	1.9
17 ^[h]^	**3**	3	—	25	7.0	35	1120	99	95	17.3	1.9
18 ^[h]^	**3**	4	—	25	12.3	62	1580	99	94	19.1	2.0
19 ^[h]^	**3**	5	—	25	25.2	126	2570	98	95	16.2	2.0
20 ^[h]^	**3**	24	—	25	53.9	270	1150	79	94	13.4	2.0
21 ^[h]^	**3**	48	—	25	57.0	285	605	67	96	16.4	2.0
22 ^[h]^	**3**	5	pentane	25	0.4	2	40	95	87	14.9	2.4
23 ^[h]^	**3**	5	benzene	25	2.0	10	200	98	90	8.5	1.9
24 ^[h]^	**3**	5	THF	25	87.0	435	8890	94	92	23.2	2.6
25 ^[h]^	**3**	5	MeCN	25	4.0	20	410	96	90	2.3	1.4
26 ^[h]^	**3**	5	CH_2_Cl_2_	25	19.3	97	1980	94	90	16.1	2.2
27 ^[h]^	**3**	5	DCE ^[g]^	25	25.6	128	2610	90	87	18.3	2.2

^[a]^ Reaction conditions: 0.1 mol% of Pd, [PA]_0_/[Pd]_0_ = 1000, *n*_Pd_ = 9.1 μmol, *V*_PA_ = 1.0 mL. ^[b]^ In units of (mol of PA) (mol of Pd)^−1^. ^[c]^ In units of (g of polymer) (mol of Pd)^−1^ h^−1^. ^[d]^ Selectivity to polymers, estimated via GPC using peak-area ratio (response factors were determined using polymer obtained in entry 1 and oligomer obtained using BF_3_∙OEt_2_ as initiator). ^[e]^ The percentages of *cis* sequences determined as prescribed in ref [102]. ^[f]^ Estimated via GPC in THF (polystyrene standard). ^[g]^ DCE—1,2-dichloroethane. ^[h]^ Reaction conditions: 0.2 mol% of Pd, [PA]_0_/[Pd]_0_ = 500, *n*_Pd_ = 18.2 μmol, *V*_PA_ = 1.0 mL; *V*_solv_. = 1 mL.

## Data Availability

Data are contained within the article and Supplementary Materials. Additionally, CIFs are openly available in www.ccdc.cam.ac.uk/data_request/cif (accessed on 12 May 2023).

## Supplementary Materials

The following supporting information can be downloaded at: https://www.mdpi.com/article/10.3390/molecules28104141/s1, spectral data (Figures S4–S94), XRD results (Figures S1–S3, Table S1), Cartesian coordinates for the calculated structures (Table S2), and polymers characterization results (Figures S95–S103).

## Sample Availability

Samples of the compounds are not available from the authors.
