# Supplementary material for: Novel Route to Cationic Palladium(II)–Cyclopentadienyl Complexes Containing Phosphine Ligands and Their Catalytic Activities
Source: Molecules. 2023 May 17;28(10):4141. doi: 10.3390/molecules28104141 (PMC10221666; doi:10.3390/molecules28104141)
Supplement: Supplementary file 1 [file molecules-28-04141-s001.zip › molecules-2408537-supplementary.pdf]

Supporting Information

Novel route to cationic palladium(II)–cyclopentadienyl complexes containing phosphine ligands and their catalytic activities

Dmitry S. Suslov[1†], Mikhail V. Bykov[1], Marina V. Pakhomova[1], Timur S. Orlov[1,2], Zorikto D. Abramov[1], Anastasia V. Suchkova[1], Igor A. Ushakov[3], Pavel A. Abramov[4,5], Alexander S. Novikov[6,7†]

[1]Irkutsk State University, ul. K. Marksa, 1, Irkutsk, 664003, Russia

[2]National Research Irkutsk State Technical University, Lermontov St, 83, Irkutsk, 664074, Russia

[3]A.E. Favorsky Irkutsk Institute of Chemistry SB RAS, Favorsky st., 1, Irkutsk, 664033, Russia

[4]Nikolaev Institute of Inorganic Chemistry SB RAS, pr-kt Akad. Lavrentieva, 3, Novosibirsk, 630090, Russia

[5]Russia Research School of Chemistry and Applied Biomedical Sciences, Tomsk Polytechnic University, Tomsk 634034, Russian Federation

[6]Institute of Chemistry, Saint Petersburg State University, Universitetskaya Nab., 7/9, Saint Petersburg, 199034, Russian Federation

[7]Research Institute of Chemistry, Peoples' Friendship University of Russia (RUDN University), Miklukho-Maklaya St., 6, Moscow, 117198, Russian Federation

[†]Correspondence should be addressed: e-mail: suslov@chem.isu.ru; suslov.dmitry@gmail.com.

[†]Correspondence should be addressed: a.s.novikov@spbu.ru

CONTENTS

S.1 XRD details 2

S.2 Computed Cartesian coordinates for palladium complexes 6

S.3 NMR spectra 20

S.4 FTIR spectra 43

S.5 ESI-MS spectra 46

S.6 UV-vis spectra 56

S.7 Reaction of [Pd(κ^2-O,O′-acac)(TOMPP)$_2$]BF_4 with BF_3·OEt_2 in the presence of MeCN 59

S.8 PPAs characterization..64

S.1 XRD details

Table S.1 – XRD experimental details

Compound	**1**	**7**	**8**
Chemical formula	$C_{48}H_{43}BF_4P_2Pd$	$C_{34.80}H_{35.20}BF_4N_{1.40}P_2Pd$	$C_{40}H_{41}BF_4P_2Pd$
M_r	874.97	728.19	776.88
Crystal system, space group	Monoclinic, $P2_1/c$	Orthorhombic, *Pnma*	Triclinic, *P*1
a, *b*, *c* (Å)	10.4737 (2), 18.7324 (4), 21.1218 (4)	16.6490 (4), 21.1894 (4), 10.7245 (2)	9.9489 (2), 9.9597 (3), 10.1331 (2)
α, β, γ (°)	90, 100.368 (1), 90	90, 90, 90	80.095 (1), 63.473 (1), 84.946 (1)
V (Å^3)	4076.38 (14)	3783.41 (14)	884.90 (4)
Z	4	4	1
μ (mm^{-1})	0.59	0.62	0.66
Crystal size (mm)	0.15 × 0.13 × 0.04	0.17 × 0.06 × 0.03	0.11 × 0.09 × 0.07
T_{min}, T_{max}	0.719, 0.746	0.695, 0.745	0.642, 0.746
No. of measured, independent and observed [$I > 2\sigma(I)$] reflections	57149, 10039, 8444	57062, 3970, 3210	10395, 7045, 6942
R_{int}	0.043	0.075	0.021
q values (°)	θ_{max} = 29.6, θ_{min} = 2.2	θ_{max} = 26.4, θ_{min} = 2.3	θ_{max} = 28.3, θ_{min} = 2.3
$(\sin \theta/\lambda)_{max}$ (Å^{-1})	0.694	0.625	0.668
Range of *h*, *k*, *l*	$-14 \leq h \leq 12$, $-24 \leq k \leq 23$, $-28 \leq l \leq 29$	$-20 \leq h \leq 20$, $-26 \leq k \leq 26$, $-13 \leq l \leq 13$	$-13 \leq h \leq 12$, $-13 \leq k \leq 13$, $-13 \leq l \leq 12$
$R[F^2 > 2\sigma(F^2)]$, $wR(F^2)$, S	0.033, 0.077, 1.06	0.061, 0.218, 1.13	0.029, 0.061, 1.02
No. of parameters, restraints	474, 6	215, 5	426, 3
H-atom treatment	H-atom parameters constrained	H atoms treated by a mixture of independent and constrained refinement	H-atom parameters constrained
Weighting scheme	$w = 1/[\sigma^2(F_o^2) + (0.0316P)^2 + 2.8122P]$ where $P = (F_o^2 + 2F_c^2)/3$	$w = 1/[\sigma^2(F_o^2) + (0.1423P)^2 + 7.1875P]$ where $P = (F_o^2 + 2F_c^2)/3$	$w = 1/[\sigma^2(F_o^2) + (0.0266P)^2]$ where $P = (F_o^2 + 2F_c^2)/3$
$\Delta\rho_{max}$, $\Delta\rho_{min}$ (e Å^{-3})	0.96, -0.61	1.61, -1.29	0.49, -0.32
Absolute structure	–	–	Flack x determined using 2498 quotients [(I+)-(I-)]/[(I+)+(I-)] (Parsons, Flack and Wagner, Acta Cryst. B69 (2013) 249-259).
Absolute structure parameter	–	–	-0.001 (11)

Computer programs: *APEX3* (Bruker-AXS, 2016), *SAINT* (Bruker-AXS, 2016), *SHELXT* 2014/5 (Sheldrick, 2014), *SHELXL2017*/1 (Sheldrick, 2017).

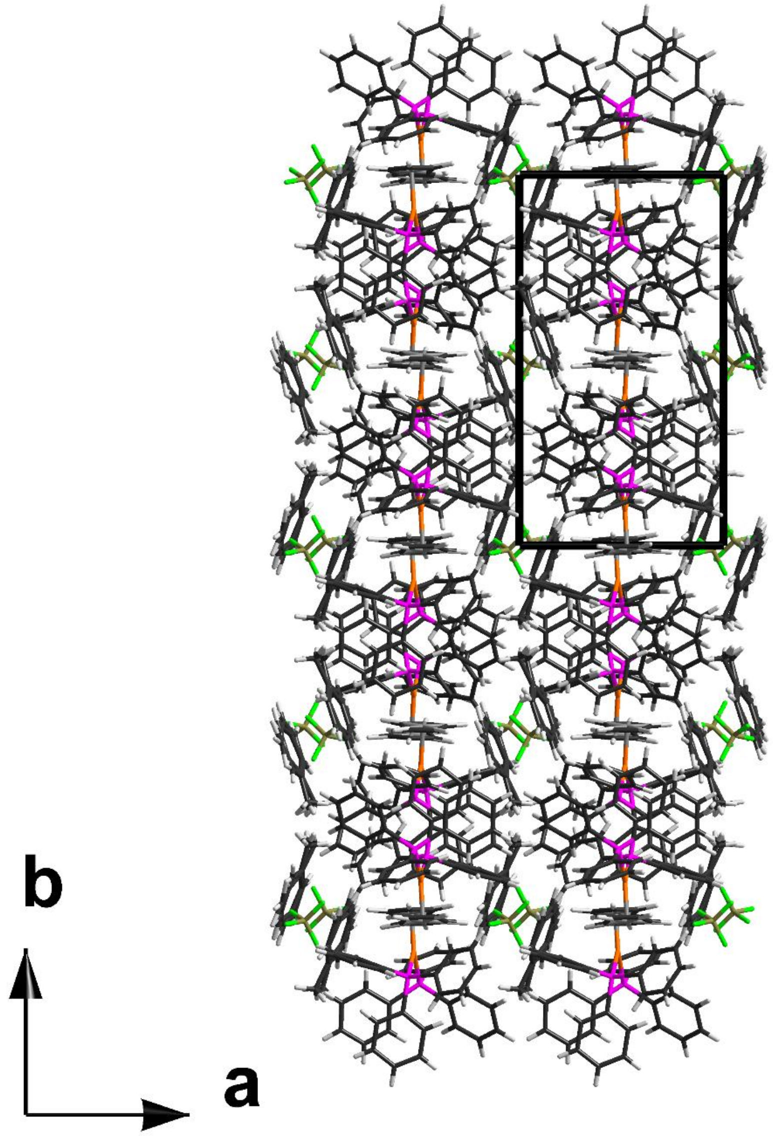

Figure S.1 – Crystal packing of **1**

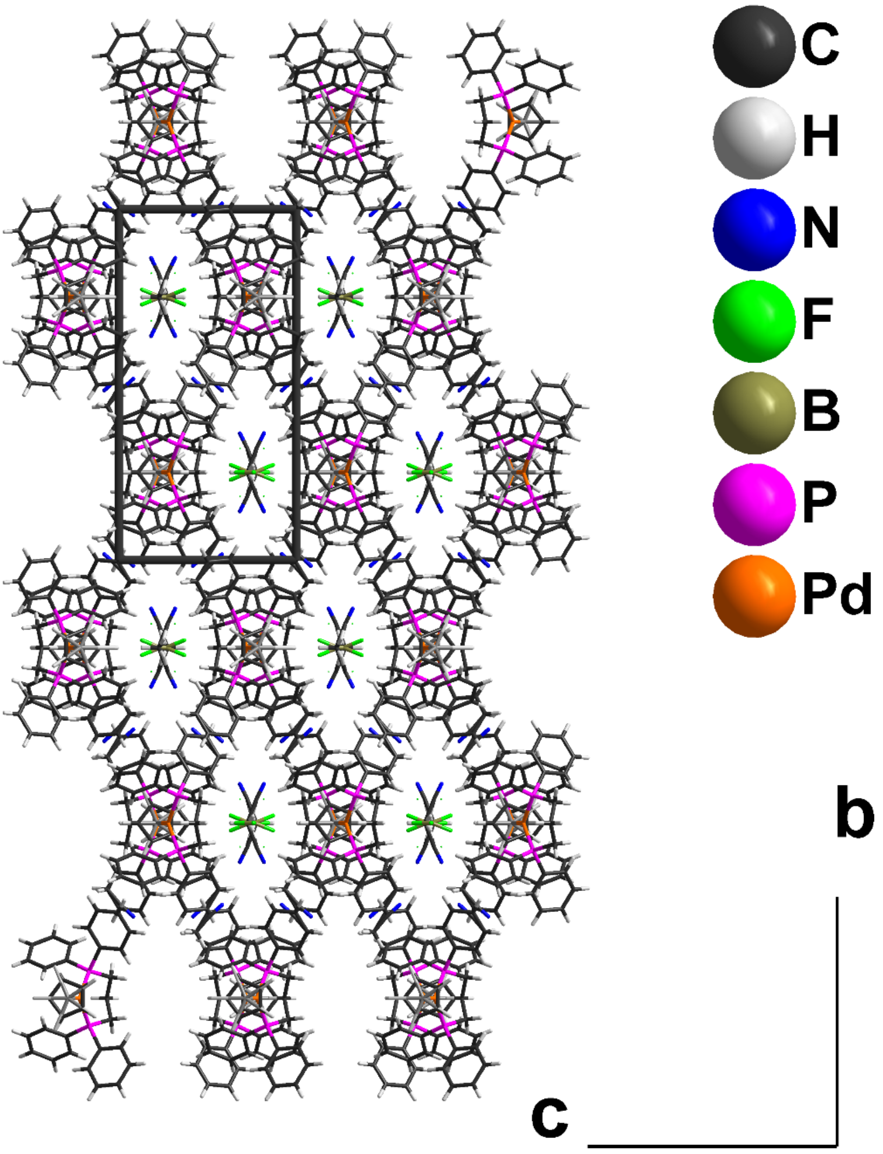

Figure S.2 – Crystal packing of **7**

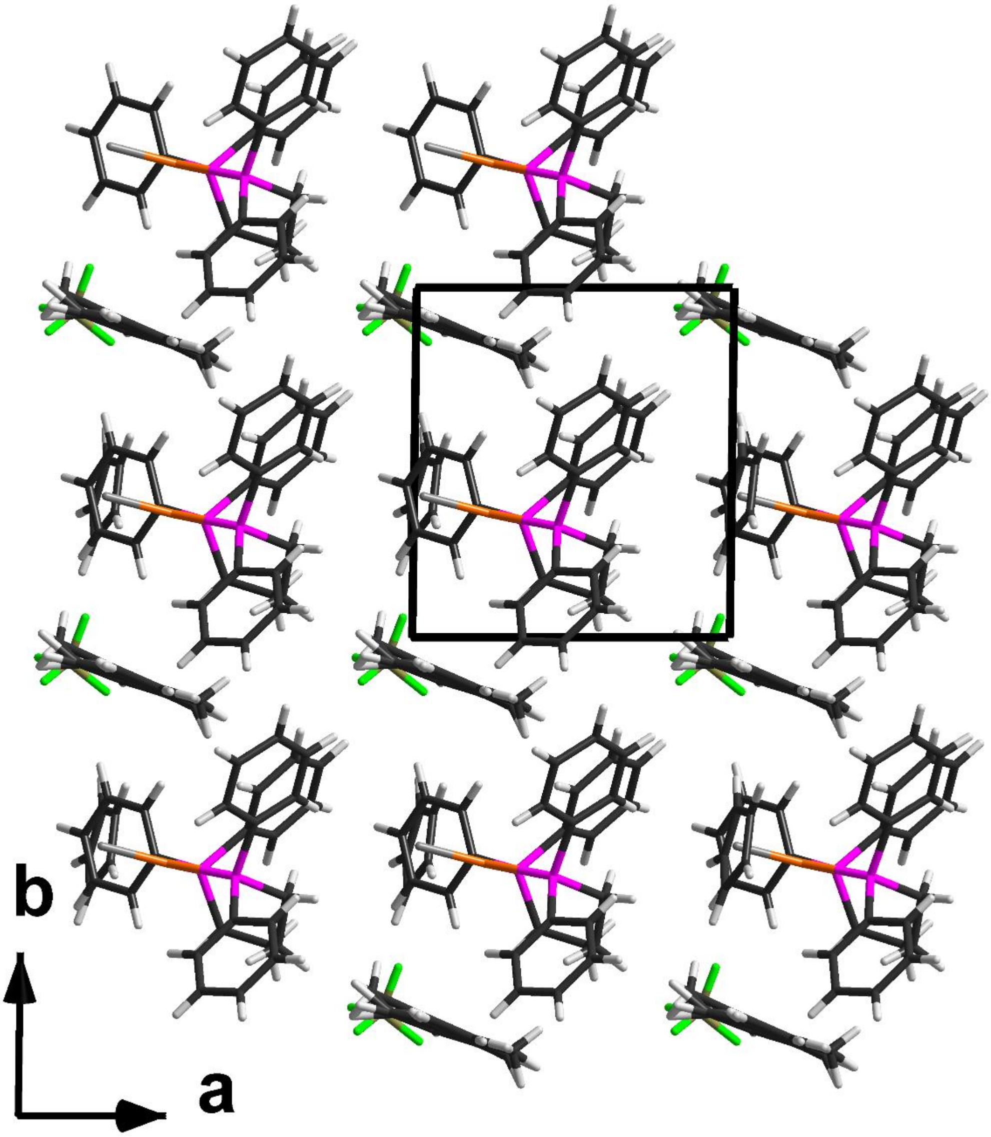

Figure S.3 – Crystal packing of **8**

S.2 Computed Cartesian coordinates for palladium complexes

Table S.2 – Cartesian atomic coordinates (Å) for model structures

Atom	*X*	*Y*	*Z*
**7**			
C	9.141966	15.892050	0.720686
H	8.474341	15.892050	0.107245
H	8.890566	15.892050	1.587226
C	9.937788	14.620686	0.469733
H	10.420892	14.710699	-0.389761
H	9.308473	13.861745	0.383165
C	12.065530	12.819587	1.112131
C	12.235350	11.639337	1.829600
H	11.851491	11.555300	2.694670
C	12.961247	10.586224	1.294447
H	13.076574	9.785922	1.793576
C	13.512328	10.702766	0.037536
H	13.992136	9.975842	-0.340814
C	13.367482	11.870302	-0.664919
H	13.761364	11.951606	-1.525807
C	12.654905	12.934010	-0.139419
H	12.567897	13.740627	-0.634118
C	10.139241	13.590881	3.155148
C	10.325710	14.057048	4.447450
H	11.007520	14.695633	4.625380
C	9.521563	13.595119	5.485582
H	9.654439	13.920631	6.367854
C	8.525953	12.662785	5.233556
H	7.966014	12.363909	5.940408
C	8.351138	12.166953	3.963775
H	7.679434	11.516926	3.796709
C	9.155285	12.620407	2.918136
H	9.038059	12.268514	2.043511
C	13.960187	15.892050	4.199714
H	13.818670	15.892050	5.083413
C	14.238225	17.002375	3.358913
H	14.135001	17.777907	3.828647
C	14.762668	16.521375	2.171711
H	15.067345	16.993899	1.447808
P	11.144674	14.237582	1.785415
Pd	12.542358	15.892050	2.373546
C	9.937788	17.163414	0.469733
H	10.420892	17.073401	-0.389761
H	9.308473	17.922355	0.383165
C	12.065530	18.964513	1.112131
C	12.235350	20.144763	1.829600
H	11.851491	20.228800	2.694670
C	12.961247	21.197876	1.294447

Atom	*X*	*Y*	*Z*
H	13.076574	21.998178	1.793576
C	13.512328	21.081334	0.037536
H	13.992136	21.808258	-0.340814
C	13.367482	19.913798	-0.664919
H	13.761364	19.832494	-1.525807
C	12.654905	18.850090	-0.139419
H	12.567897	18.043473	-0.634118
C	10.139241	18.193219	3.155148
C	10.325710	17.727052	4.447450
H	11.007520	17.088467	4.625380
C	9.521563	18.188981	5.485582
H	9.654439	17.863469	6.367854
C	8.525953	19.121315	5.233556
H	7.966014	19.420191	5.940408
C	8.351138	19.617147	3.963775
H	7.679434	20.267174	3.796709
C	9.155285	19.163693	2.918136
H	9.038059	19.515586	2.043511
P	11.144674	17.546518	1.785415
C	14.238225	14.781725	3.358913
H	14.135001	14.006193	3.828647
C	14.762668	15.262725	2.171711
H	15.067345	14.790201	1.447808
C	17.466466	15.892050	4.641564
H	16.798841	15.892050	5.255005
H	17.215066	15.892050	3.775024
C	18.262288	17.163414	4.892517
H	18.745392	17.073401	5.752011
H	17.632973	17.922355	4.979085
C	20.390030	18.964513	4.250119
C	20.559850	20.144763	3.532650
H	20.175991	20.228800	2.667580
C	21.285747	21.197876	4.067803
H	21.401074	21.998178	3.568674
C	21.836828	21.081334	5.324714
H	22.316636	21.808258	5.703064
C	21.691982	19.913798	6.027169
H	22.085864	19.832494	6.888057
C	20.979405	18.850090	5.501669
H	20.892397	18.043473	5.996368
C	18.463741	18.193219	2.207102
C	18.650210	17.727052	0.914800
H	19.332020	17.088467	0.736870
C	17.846063	18.188981	-0.123332
H	17.978939	17.863469	-1.005604
C	16.850453	19.121315	0.128694
H	16.290514	19.420191	-0.578158
C	16.675638	19.617147	1.398475
H	16.003934	20.267174	1.565541

Atom	*X*	*Y*	*Z*
C	17.479785	19.163693	2.444114
H	17.362559	19.515586	3.318739
C	22.284687	15.892050	1.162536
H	22.143170	15.892050	0.278837
C	22.562725	14.781725	2.003337
H	22.459501	14.006193	1.533604
C	23.087168	15.262725	3.190539
H	23.391845	14.790201	3.914443
P	19.469174	17.546518	3.576835
Pd	20.866858	15.892050	2.988704
C	18.262288	14.620686	4.892517
H	18.745392	14.710699	5.752011
H	17.632973	13.861745	4.979085
C	20.390030	12.819587	4.250119
C	20.559850	11.639337	3.532650
H	20.175991	11.555300	2.667580
C	21.285747	10.586224	4.067803
H	21.401074	9.785922	3.568674
C	21.836828	10.702766	5.324714
H	22.316636	9.975842	5.703064
C	21.691982	11.870302	6.027169
H	22.085864	11.951606	6.888057
C	20.979405	12.934010	5.501669
H	20.892397	13.740627	5.996368
C	18.463741	13.590881	2.207102
C	18.650210	14.057048	0.914800
H	19.332020	14.695633	0.736870
C	17.846063	13.595119	-0.123332
H	17.978939	13.920631	-1.005604
C	16.850453	12.662785	0.128694
H	16.290514	12.363909	-0.578158
C	16.675638	12.166953	1.398475
H	16.003934	11.516926	1.565541
C	17.479785	12.620407	2.444114
H	17.362559	12.268514	3.318739
P	19.469174	14.237582	3.576835
C	22.562725	17.002375	2.003337
H	22.459501	17.777907	1.533604
C	23.087168	16.521375	3.190539
H	23.391845	16.993899	3.914443
C	15.831534	26.486750	-4.641564
H	16.499159	26.486750	-5.255005
H	16.082934	26.486750	-3.775024
C	15.035712	27.758114	-4.892517
H	14.552608	27.668101	-5.752011
H	15.665027	28.517055	-4.979085
C	12.907970	29.559213	-4.250119
C	12.738150	30.739463	-3.532650
H	13.122009	30.823500	-2.667580

Atom	*X*	*Y*	*Z*
C	12.012254	31.792576	-4.067803
H	11.896926	32.592878	-3.568674
C	11.461172	31.676034	-5.324714
H	10.981364	32.402958	-5.703064
C	11.606018	30.508498	-6.027169
H	11.212136	30.427194	-6.888057
C	12.318595	29.444790	-5.501669
H	12.405603	28.638173	-5.996368
C	14.834259	28.787919	-2.207102
C	14.647790	28.321752	-0.914800
H	13.965980	27.683167	-0.736870
C	15.451937	28.783681	0.123332
H	15.319061	28.458169	1.005604
C	16.447547	29.716015	-0.128694
H	17.007486	30.014891	0.578158
C	16.622362	30.211847	-1.398475
H	17.294066	30.861874	-1.565541
C	15.818215	29.758393	-2.444114
H	15.935441	30.110286	-3.318739
C	11.013314	26.486750	-1.162536
H	11.154830	26.486750	-0.278837
C	10.735275	25.376425	-2.003337
H	10.838499	24.600893	-1.533604
C	10.210832	25.857425	-3.190539
H	9.906155	25.384901	-3.914443
P	13.828826	28.141218	-3.576835
Pd	12.431142	26.486750	-2.988704
C	15.035712	25.215386	-4.892517
H	14.552608	25.305399	-5.752011
H	15.665027	24.456445	-4.979085
C	12.907970	23.414287	-4.250119
C	12.738150	22.234037	-3.532650
H	13.122009	22.150000	-2.667580
C	12.012254	21.180924	-4.067803
H	11.896926	20.380622	-3.568674
C	11.461172	21.297466	-5.324714
H	10.981364	20.570542	-5.703064
C	11.606018	22.465002	-6.027169
H	11.212136	22.546306	-6.888057
C	12.318595	23.528710	-5.501669
H	12.405603	24.335327	-5.996368
C	14.834259	24.185581	-2.207102
C	14.647790	24.651748	-0.914800
H	13.965980	25.290333	-0.736870
C	15.451937	24.189819	0.123332
H	15.319061	24.515331	1.005604
C	16.447547	23.257485	-0.128694
H	17.007486	22.958609	0.578158
C	16.622362	22.761653	-1.398475

Atom	*X*	*Y*	*Z*
H	17.294066	22.111626	-1.565541
C	15.818215	23.215107	-2.444114
H	15.935441	22.863214	-3.318739
P	13.828826	24.832282	-3.576835
C	10.735275	27.597075	-2.003337
H	10.838499	28.372607	-1.533604
C	10.210832	27.116075	-3.190539
H	9.906155	27.588599	-3.914443
**8**			
C	5.814225	2.107158	3.143239
H	5.376353	1.552299	3.837020
H	5.370704	1.899813	2.283152
C	7.252533	1.656694	3.037448
H	7.678981	2.166469	2.304041
H	7.255127	0.702884	2.772075
C	8.646072	3.240563	4.569621
H	8.519565	3.789311	3.755104
H	9.616289	3.206398	4.763460
C	8.654421	5.612911	6.264961
C	9.507417	6.197552	5.324500
H	9.698846	5.746743	4.510189
C	10.075657	7.441820	5.582701
H	10.650298	7.838219	4.938265
C	9.817747	8.096770	6.743709
H	10.207905	8.947787	6.906528
C	8.978310	7.519518	7.700308
H	8.802624	7.975131	8.514897
C	8.406245	6.289855	7.460038
H	7.837727	5.899006	8.113726
C	8.335647	2.991982	7.347075
C	9.696827	2.994250	7.685067
H	10.283968	3.610530	7.263805
C	10.192437	2.114280	8.621046
H	11.118966	2.111414	8.830036
C	9.346019	1.247862	9.245032
H	9.691132	0.636526	9.884922
C	7.981270	1.242509	8.959038
H	7.399152	0.641032	9.408909
C	7.479488	2.126137	8.007818
H	6.549807	2.135273	7.811550
C	3.787919	4.123186	2.906555
C	3.384293	5.423434	2.620561
H	4.000070	6.141334	2.707148
C	2.076176	5.674720	2.207260
H	1.801305	6.564216	2.017662
C	1.180618	4.631623	2.072781
H	0.296407	4.803875	1.772613
C	1.563032	3.352972	2.370429
H	0.938997	2.640625	2.297864

Atom	*X*	*Y*	*Z*
C	2.875772	3.097717	2.783730
H	3.141165	2.207656	2.981433
C	3.361022	4.646271	6.196825
H	2.611720	4.349445	5.604675
C	3.910700	5.963588	6.221928
H	3.637061	6.729090	5.639927
C	4.820075	6.011559	7.245767
H	5.332643	6.818916	7.537247
C	4.862805	4.716872	7.865270
H	5.382465	4.487934	8.688600
C	3.877715	3.903701	7.274456
H	3.579365	3.000411	7.581930
C	6.485867	4.843588	2.400015
C	7.104502	5.998550	2.829453
H	7.019627	6.266261	3.736976
C	7.847409	6.774597	1.954438
H	8.259342	7.573021	2.261043
C	7.988154	6.387976	0.638330
H	8.505420	6.914472	0.039716
C	7.368632	5.221386	0.190961
H	7.467925	4.948659	-0.713307
C	6.609135	4.458958	1.064183
H	6.172895	3.674486	0.753768
C	8.134962	1.803249	4.294385
H	8.917003	1.203041	4.201272
H	7.616441	1.497286	5.080294
P	5.471063	3.851823	3.535113
P	7.788989	4.041005	5.964265
Pd	5.567994	4.376724	5.734215
C	0.390965	3.456766	12.108553
H	-0.046907	2.901907	12.802334
H	-0.052556	3.249422	11.248466
C	1.829273	3.006302	12.002762
H	2.255721	3.516078	11.269355
H	1.831867	2.052493	11.737389
C	3.222812	4.590172	13.534935
H	3.096305	5.138920	12.720418
H	4.193029	4.556006	13.728774
C	3.231161	6.962519	15.230275
C	4.084157	7.547161	14.289814
H	4.275586	7.096352	13.475503
C	4.652397	8.791428	14.548015
H	5.227038	9.187827	13.903579
C	4.394487	9.446378	15.709023
H	4.784645	10.297395	15.871842
C	3.555050	8.869126	16.665622
H	3.379364	9.324739	17.480211
C	2.982985	7.639463	16.425352
H	2.414467	7.248614	17.079040

Atom	*X*	*Y*	*Z*
C	2.912387	4.341591	16.312389
C	4.273567	4.343858	16.650381
H	4.860708	4.960139	16.229119
C	4.769177	3.463889	17.586360
H	5.695706	3.461023	17.795350
C	3.922759	2.597471	18.210346
H	4.267872	1.986134	18.850236
C	2.558010	2.592118	17.924352
H	1.975892	1.990640	18.374223
C	2.056228	3.475746	16.973132
H	1.126547	3.484882	16.776864
C	-1.635341	5.472795	11.871869
C	-2.038967	6.773043	11.585875
H	-1.423190	7.490943	11.672462
C	-3.347084	7.024329	11.172574
H	-3.621955	7.913825	10.982976
C	-4.242642	5.981232	11.038095
H	-5.126853	6.153484	10.737927
C	-3.860229	4.702580	11.335743
H	-4.484263	3.990234	11.263178
C	-2.547488	4.447326	11.749044
H	-2.282095	3.557264	11.946747
C	-2.062238	5.995880	15.162139
H	-2.811540	5.699054	14.569989
C	-1.512560	7.313196	15.187242
H	-1.786199	8.078699	14.605241
C	-0.603185	7.361167	16.211081
H	-0.090617	8.168524	16.502561
C	-0.560455	6.066480	16.830584
H	-0.040795	5.837542	17.653914
C	-1.545545	5.253310	16.239770
H	-1.843895	4.350019	16.547244
C	1.062607	6.193197	11.365329
C	1.681242	7.348158	11.794767
H	1.596367	7.615870	12.702290
C	2.424149	8.124206	10.919752
H	2.836082	8.922629	11.226357
C	2.564894	7.737585	9.603644
H	3.082160	8.264080	9.005030
C	1.945372	6.570995	9.156275
H	2.044665	6.298268	8.252007
C	1.185875	5.808567	10.029497
H	0.749635	5.024094	9.719082
C	2.711702	3.152857	13.259699
H	3.493743	2.552649	13.166586
H	2.193181	2.846895	14.045608
P	0.047803	5.201431	12.500427
P	2.365729	5.390613	14.929579
Pd	0.144734	5.726333	14.699529

Atom	*X*	*Y*	*Z*
C	1.288585	0.757549	-5.822075
H	0.850713	0.202690	-5.128294
H	0.845064	0.550205	-6.682162
C	2.726893	0.307085	-5.927866
H	3.153341	0.816860	-6.661273
H	2.729488	-0.646724	-6.193239
C	4.120432	1.890955	-4.395693
H	3.993925	2.439702	-5.210210
H	5.090649	1.856789	-4.201854
C	4.128781	4.263302	-2.700353
C	4.981777	4.847944	-3.640814
H	5.173206	4.397135	-4.455125
C	5.550017	6.092211	-3.382613
H	6.124658	6.488610	-4.027049
C	5.292107	6.747161	-2.221605
H	5.682265	7.598178	-2.058786
C	4.452670	6.169909	-1.265006
H	4.276984	6.625522	-0.450417
C	3.880605	4.940246	-1.505276
H	3.312087	4.549397	-0.851588
C	3.810007	1.642374	-1.618239
C	5.171187	1.644641	-1.280247
H	5.758328	2.260922	-1.701509
C	5.666797	0.764672	-0.344268
H	6.593326	0.761806	-0.135278
C	4.820379	-0.101746	0.279718
H	5.165492	-0.713083	0.919608
C	3.455630	-0.107099	-0.006276
H	2.873512	-0.708577	0.443595
C	2.953848	0.776529	-0.957496
H	2.024167	0.785665	-1.153764
C	-0.737721	2.773578	-6.058759
C	-1.141347	4.073826	-6.344753
H	-0.525570	4.791726	-6.258166
C	-2.449464	4.325112	-6.758054
H	-2.724335	5.214608	-6.947652
C	-3.345022	3.282015	-6.892533
H	-4.229233	3.454266	-7.192701
C	-2.962608	2.003363	-6.594885
H	-3.586643	1.291016	-6.667450
C	-1.649868	1.748109	-6.181584
H	-1.384475	0.858047	-5.983881
C	-1.164618	3.296663	-2.768489
H	-1.913920	2.999837	-3.360639
C	-0.614940	4.613979	-2.743386
H	-0.888579	5.379481	-3.325387
C	0.294435	4.661950	-1.719547
H	0.807003	5.469307	-1.428067
C	0.337165	3.367263	-1.100044

Atom	*X*	*Y*	*Z*
H	0.856825	3.138325	-0.276714
C	-0.647925	2.554093	-1.690858
H	-0.946275	1.650802	-1.383384
C	1.960227	3.493979	-6.565299
C	2.578862	4.648941	-6.135861
H	2.493987	4.916652	-5.228338
C	3.321769	5.424989	-7.010876
H	3.733702	6.223412	-6.704271
C	3.462514	5.038368	-8.326984
H	3.979780	5.564863	-8.925598
C	2.842992	3.871778	-8.774353
H	2.942285	3.599050	-9.678621
C	2.083495	3.109350	-7.901131
H	1.647255	2.324877	-8.211546
C	3.609322	0.453640	-4.670929
H	4.391363	-0.146568	-4.764042
H	3.090801	0.147677	-3.885020
P	0.945423	2.502214	-5.430201
P	3.263349	2.691396	-3.001049
Pd	1.042354	3.027115	-3.231099
C	5.444138	-1.154883	4.948853
H	6.302341	-1.545824	4.830986
C	5.094386	-0.534814	6.168136
H	5.716512	-0.504561	6.885460
C	3.926963	-0.011981	6.299926
H	3.709345	0.371585	7.141330
C	2.933763	0.033441	5.276984
C	1.616994	0.673104	5.440153
H	1.554821	1.068334	6.334586
H	1.509047	1.375015	4.764580
H	0.912045	0.001821	5.329368
C	3.279422	-0.596929	4.107907
H	2.652699	-0.635471	3.394501
C	4.527666	-1.180090	3.951014
H	4.744981	-1.603875	3.129388
		**1**	
C	4.131747	9.614030	14.260867
H	5.127456	9.696864	14.214576
C	3.206414	9.720242	13.153041
H	3.459042	9.937032	12.210288
C	1.884451	9.721366	13.663530
H	1.048642	9.917569	13.150070
C	1.971141	9.423896	15.027327
H	1.201863	9.396678	15.665885
C	3.377051	9.394861	15.397572
H	3.733797	9.307268	16.327485
C	2.324095	6.442072	10.791120
C	1.538230	7.581377	10.645266
H	1.562423	8.256155	11.313514

Atom	*X*	*Y*	*Z*
C	0.718776	7.744911	9.535778
H	0.200659	8.535399	9.440827
C	0.657441	6.760523	8.571521
H	0.091124	6.867710	7.816342
C	1.423193	5.617097	8.708233
H	1.378581	4.937598	8.045740
C	2.253401	5.455999	9.803593
H	2.778657	4.668788	9.884768
C	5.095479	6.829833	11.506469
C	5.375483	6.704701	10.142049
H	4.701580	6.410864	9.540349
C	6.647053	7.013598	9.672282
H	6.838082	6.935802	8.744946
C	7.627600	7.429270	10.535564
H	8.492630	7.635495	10.201263
C	7.368882	7.551030	11.886687
H	8.054690	7.831492	12.481822
C	6.098097	7.259929	12.369335
H	5.913454	7.355670	13.296173
C	3.702123	4.520877	12.447249
C	4.971940	4.001803	12.731269
H	5.728425	4.576213	12.742426
C	5.124455	2.645390	12.996798
H	5.985586	2.297735	13.195363
C	4.034059	1.801120	12.974359
H	4.146998	0.874747	13.152563
C	2.776582	2.304460	12.691585
H	2.025375	1.722463	12.676481
C	2.608682	3.657314	12.430004
H	1.742667	3.996764	12.237921
C	3.772622	4.963711	15.768648
C	4.986122	5.663928	15.790879
H	4.990262	6.601204	15.635780
C	6.179220	5.005110	16.036255
H	6.997059	5.487806	16.044670
C	6.175284	3.638019	16.269580
H	6.991829	3.182073	16.436543
C	4.986363	2.941361	16.258984
H	4.988164	2.005790	16.424202
C	3.782542	3.593249	16.009245
H	2.968461	3.103378	16.002846
C	1.718249	6.571326	17.026691
C	2.553874	6.617782	18.145113
H	3.449350	6.308042	18.082450
C	2.074032	7.116439	19.348097
H	2.643624	7.141203	20.108158
C	0.773463	7.578380	19.449696
H	0.450267	7.916106	20.276846
C	-0.057818	7.545785	18.337300

Atom	*X*	*Y*	*Z*
H	-0.949109	7.867158	18.403017
C	0.410140	7.046005	17.130576
H	-0.162537	7.026579	16.372550
C	0.867610	4.975513	14.857995
C	0.435282	3.831900	15.527636
H	0.916162	3.517064	16.284435
C	-0.690984	3.152476	15.094437
H	-0.969240	2.361800	15.541826
C	-1.411822	3.621535	14.013829
H	-2.175990	3.145095	13.712231
C	-1.024428	4.783693	13.367667
H	-1.534379	5.115425	12.637981
C	0.113279	5.464054	13.789854
H	0.377916	6.263347	13.349923
P	3.480906	6.317877	12.195432
P	2.297014	5.968143	15.401520
Pd	2.864715	7.630081	13.965626
C	0.640024	18.980230	16.904523
H	-0.355685	19.063064	16.950814
C	1.565357	19.086442	18.012348
H	1.312729	19.303232	18.955101
C	2.887320	19.087566	17.501859
H	3.723129	19.283769	18.015319
C	2.800631	18.790096	16.138062
H	3.569908	18.762878	15.499504
C	1.394720	18.761061	15.767817
H	1.037975	18.673468	14.837904
C	2.447677	15.808272	20.374269
C	3.233541	16.947577	20.520123
H	3.209348	17.622355	19.851875
C	4.052995	17.111111	21.629611
H	4.571112	17.901599	21.724562
C	4.114330	16.126723	22.593868
H	4.680647	16.233910	23.349047
C	3.348579	14.983297	22.457156
H	3.393190	14.303798	23.119649
C	2.518370	14.822199	21.361797
H	1.993114	14.034988	21.280621
C	-0.323707	16.196033	19.658920
C	-0.603712	16.070901	21.023341
H	0.070191	15.777064	21.625040
C	-1.875281	16.379798	21.493107
H	-2.066311	16.302002	22.420443
C	-2.855829	16.795470	20.629826
H	-3.720859	17.001695	20.964126
C	-2.597111	16.917230	19.278702
H	-3.282919	17.197692	18.683568
C	-1.326326	16.626129	18.796054
H	-1.141683	16.721870	17.869216

Atom	*X*	*Y*	*Z*
C	1.069648	13.887077	18.718141
C	-0.200169	13.368003	18.434120
H	-0.956654	13.942413	18.422963
C	-0.352684	12.011590	18.168591
H	-1.213815	11.663935	17.970026
C	0.737713	11.167320	18.191030
H	0.624774	10.240947	18.012826
C	1.995190	11.670660	18.473804
H	2.746396	11.088663	18.488909
C	2.163090	13.023514	18.735385
H	3.029104	13.362964	18.927468
C	0.999150	14.329911	15.396741
C	-0.214351	15.030128	15.374510
H	-0.218491	15.967404	15.529610
C	-1.407449	14.371310	15.129134
H	-2.225288	14.854006	15.120720
C	-1.403513	13.004219	14.895809
H	-2.220058	12.548273	14.728846
C	-0.214592	12.307561	14.906406
H	-0.216393	11.371990	14.741188
C	0.989229	12.959449	15.156144
H	1.803310	12.469578	15.162544
C	3.053522	15.937526	14.138698
C	2.217897	15.983982	13.020276
H	1.322421	15.674242	13.082940
C	2.697739	16.482639	11.817292
H	2.128148	16.507403	11.057231
C	3.998309	16.944580	11.715693
H	4.321505	17.282306	10.888543
C	4.829589	16.911985	12.828090
H	5.720880	17.233358	12.762372
C	4.361631	16.412205	14.034814
H	4.934308	16.392779	14.792839
C	3.904161	14.341713	16.307394
C	4.336489	13.198100	15.637753
H	3.855609	12.883264	14.880954
C	5.462755	12.518676	16.070952
H	5.741011	11.728000	15.623563
C	6.183594	12.987735	17.151560
H	6.947761	12.511295	17.453158
C	5.796199	14.149893	17.797723
H	6.306150	14.481625	18.527408
C	4.658492	14.830254	17.375536
H	4.393855	15.629547	17.815466
P	1.290865	15.684077	18.969957
P	2.474757	15.334343	15.763869
Pd	1.907056	16.996281	17.199763
C	2.540667	9.118370	6.516060
H	1.544958	9.035536	6.562351

Atom	*X*	*Y*	*Z*
C	3.466000	9.012158	7.623885
H	3.213372	8.795368	8.566638
C	4.787963	9.011034	7.113396
H	5.623772	8.814831	7.626856
C	4.701274	9.308504	5.749599
H	5.470551	9.335722	5.111041
C	3.295363	9.337539	5.379354
H	2.938618	9.425132	4.449441
C	4.348320	12.290328	9.985806
C	5.134184	11.151023	10.131660
H	5.109991	10.476245	9.463412
C	5.953638	10.987489	11.241148
H	6.471755	10.197001	11.336099
C	6.014973	11.971877	12.205405
H	6.581290	11.864690	12.960584
C	5.249221	13.115303	12.068693
H	5.293833	13.794802	12.731186
C	4.419013	13.276401	10.973334
H	3.893757	14.063612	10.892158
C	1.576936	11.902567	9.270457
C	1.296931	12.027699	10.634877
H	1.970834	12.321536	11.236577
C	0.025361	11.718802	11.104644
H	-0.165668	11.796598	12.031980
C	-0.955186	11.303130	10.241362
H	-1.820216	11.096905	10.575663
C	-0.696468	11.181370	8.890239
H	-1.382276	10.900908	8.295105
C	0.574317	11.472471	8.407591
H	0.758960	11.376730	7.480753
C	2.970291	14.211523	8.329677
C	1.700474	14.730597	8.045657
H	0.943989	14.156187	8.034500
C	1.547959	16.087010	7.780128
H	0.686828	16.434665	7.581563
C	2.638355	16.931280	7.802567
H	2.525417	17.857653	7.624363
C	3.895832	16.427940	8.085341
H	4.647039	17.009937	8.100446
C	4.063732	15.075086	8.346922
H	4.929747	14.735636	8.539005
C	2.899793	13.768689	5.008278
C	1.686292	13.068472	4.986047
H	1.682152	12.131196	5.141146
C	0.493194	13.727290	4.740671
H	-0.324645	13.244594	4.732257
C	0.497130	15.094381	4.507346
H	-0.319415	15.550327	4.340383
C	1.686051	15.791039	4.517943

Atom	*X*	*Y*	*Z*
H	1.684250	16.726610	4.352724
C	2.889872	15.139151	4.767681
H	3.703953	15.629022	4.774081
C	4.954165	12.161074	3.750235
C	4.118540	12.114618	2.631813
H	3.223064	12.424358	2.694476
C	4.598382	11.615961	1.428829
H	4.028790	11.591197	0.668768
C	5.898952	11.154020	1.327230
H	6.222147	10.816294	0.500080
C	6.730232	11.186615	2.439627
H	7.621523	10.865242	2.373909
C	6.262274	11.686395	3.646351
H	6.834951	11.705821	4.404376
C	5.804804	13.756887	5.918931
C	6.237132	14.900500	5.249290
H	5.756252	15.215336	4.492491
C	7.363398	15.579924	5.682489
H	7.641654	16.370600	5.235100
C	8.084236	15.110865	6.763097
H	8.848404	15.587305	7.064695
C	7.696842	13.948707	7.409260
H	8.206793	13.616975	8.138945
C	6.559135	13.268346	6.987073
H	6.294498	12.469053	7.427003
P	3.191508	12.414523	8.581494
P	4.375400	12.764257	5.375406
Pd	3.807699	11.102319	6.811300

S.3 NMR spectra

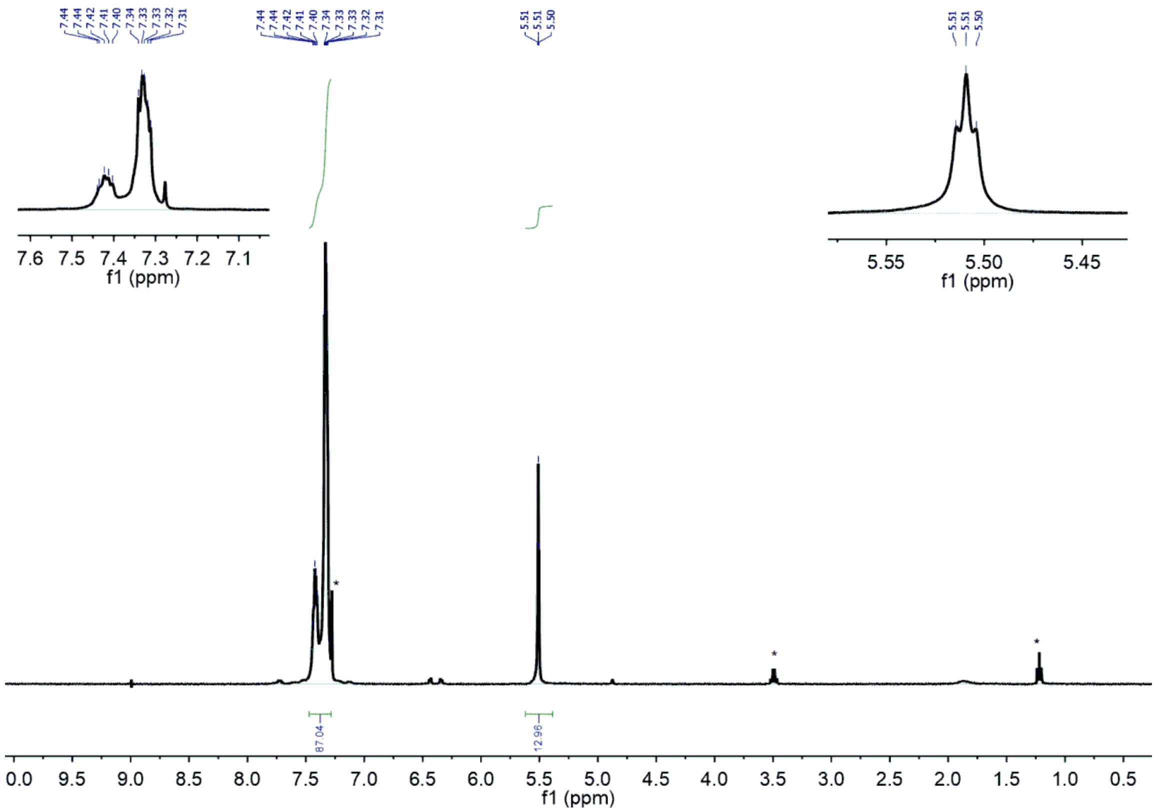

Figure S.4 – ^{1}H NMR (400 MHz, $CDCl_3$, 25°C) spectrum of **1**

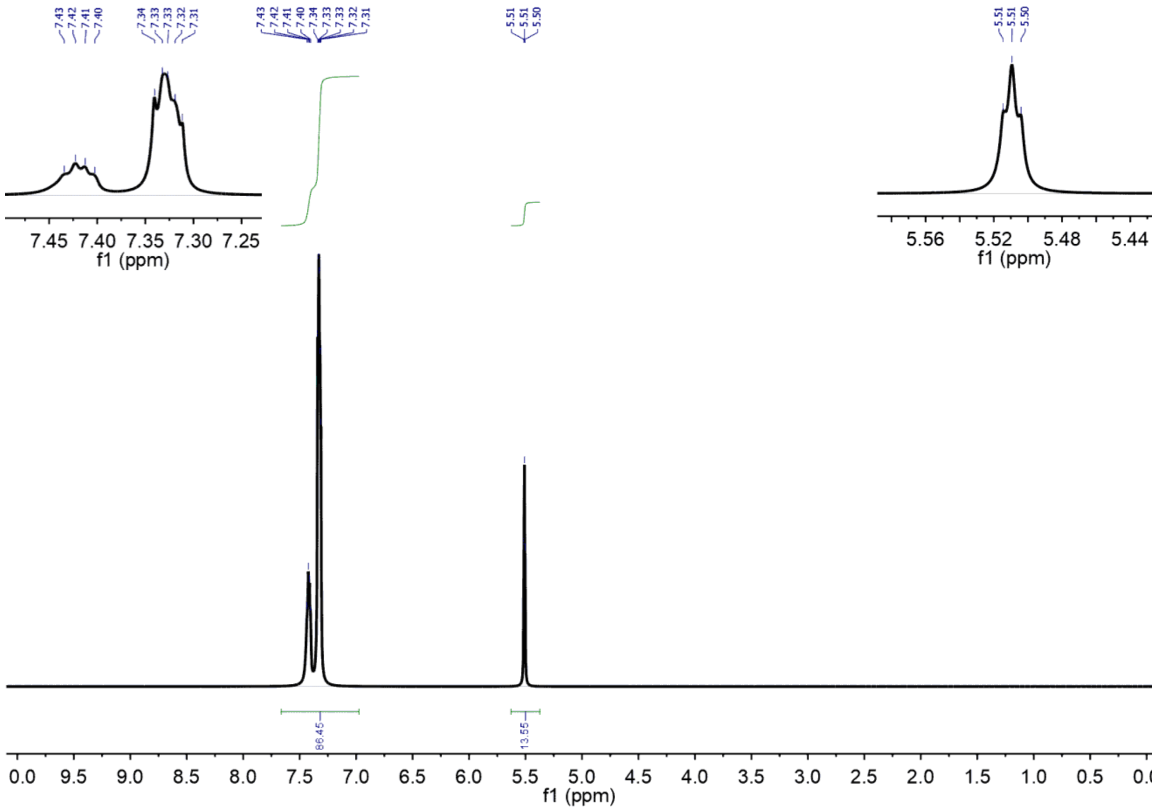

Figure S.5 – ^{1}H (deconvoluted) NMR (400 MHz, $CDCl_3$, 25°C) spectrum of **1**

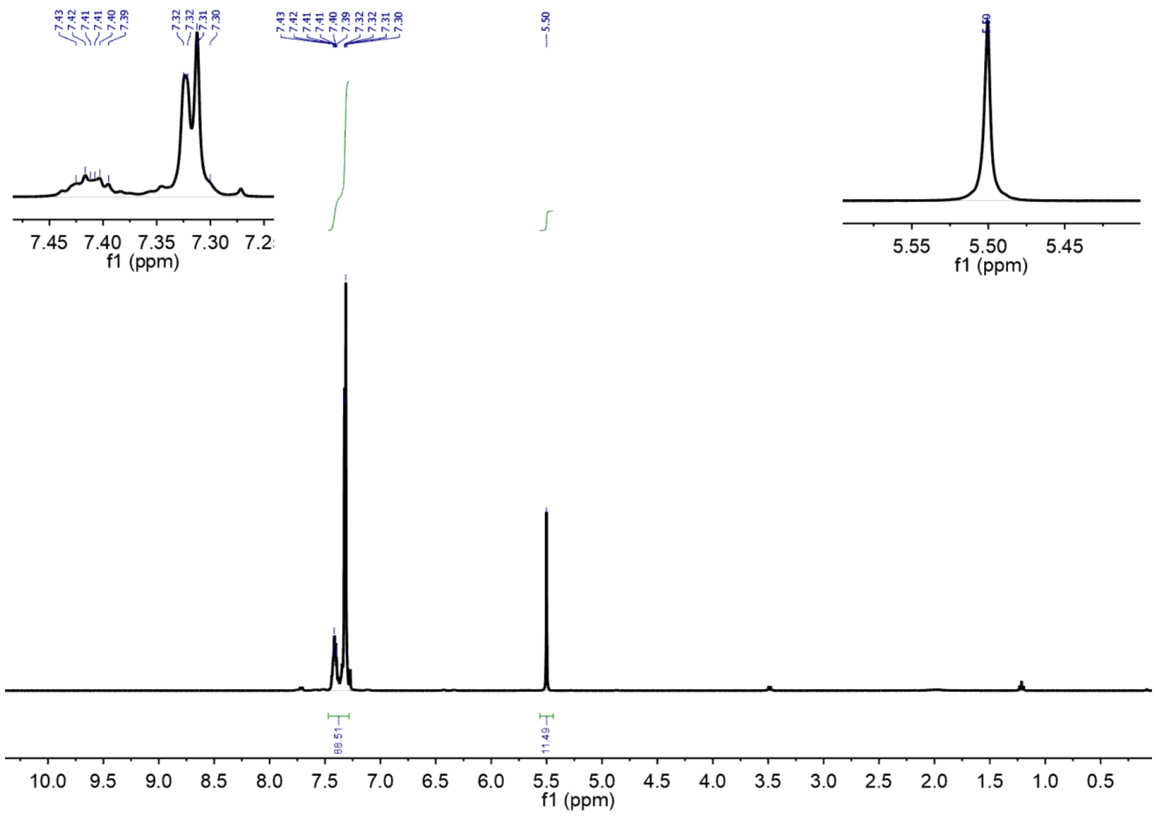

Figure S.6 – $^{1}H\{^{31}P\}$ NMR (400 MHz, $CDCl_3$, 25°C) spectrum of **1**

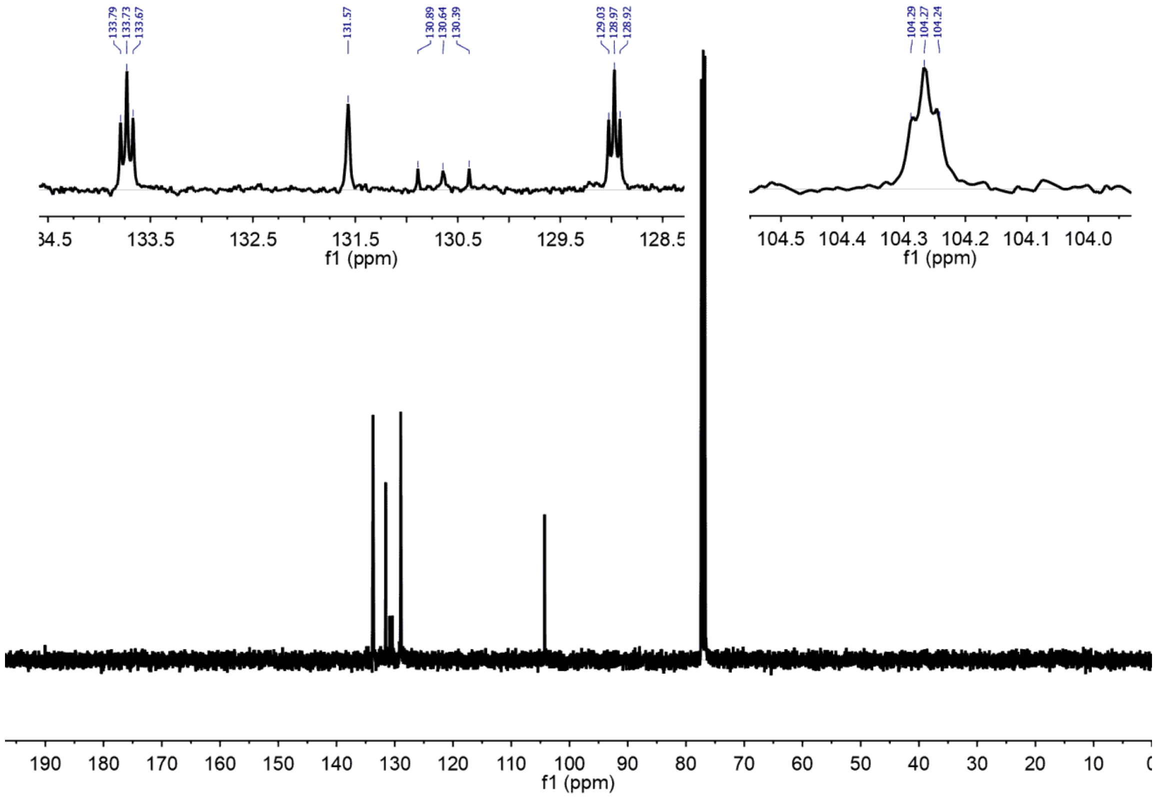

Figure S.7 – $^{13}C\{^{1}H\}$ NMR (101 MHz, $CDCl_3$, 25°C) spectrum of **1**

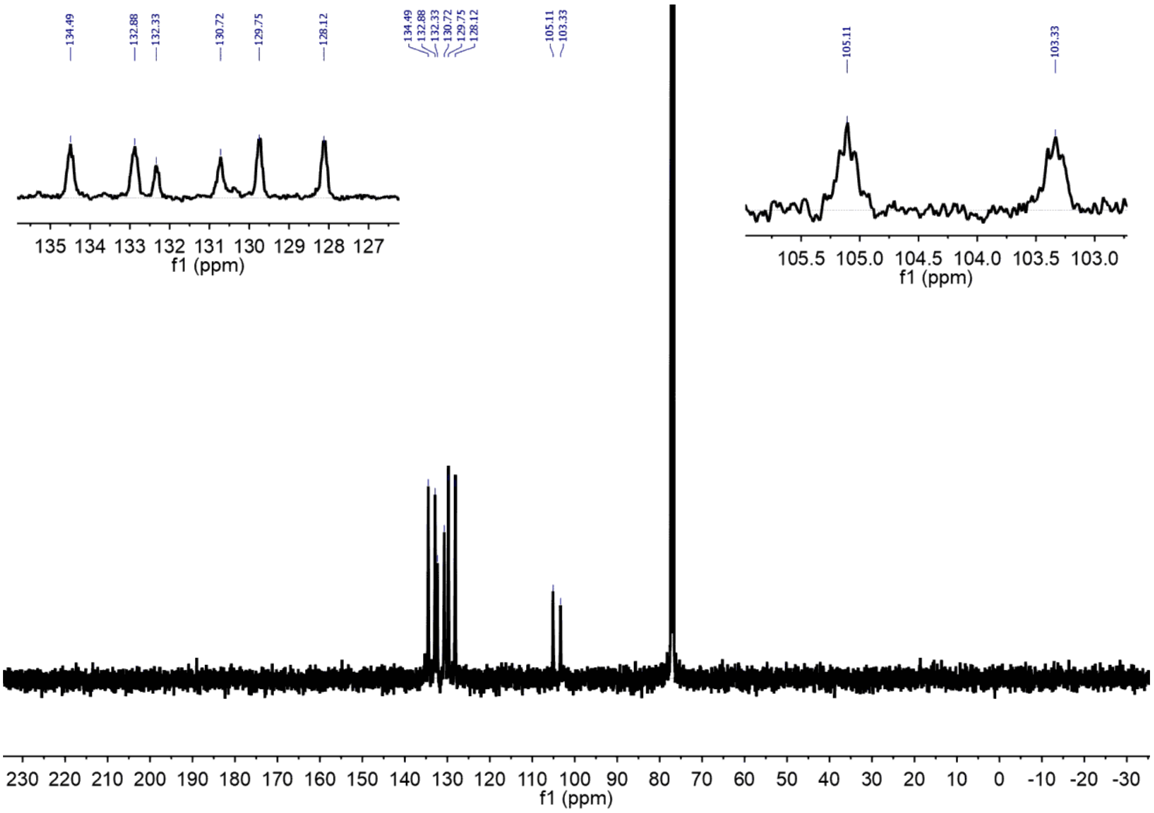

Figure S.8 – $^{13}C\{^{1}H$, gated decoupling mode$\}$ NMR (101 MHz, $CDCl_3$, 25°C) spectrum of **1**

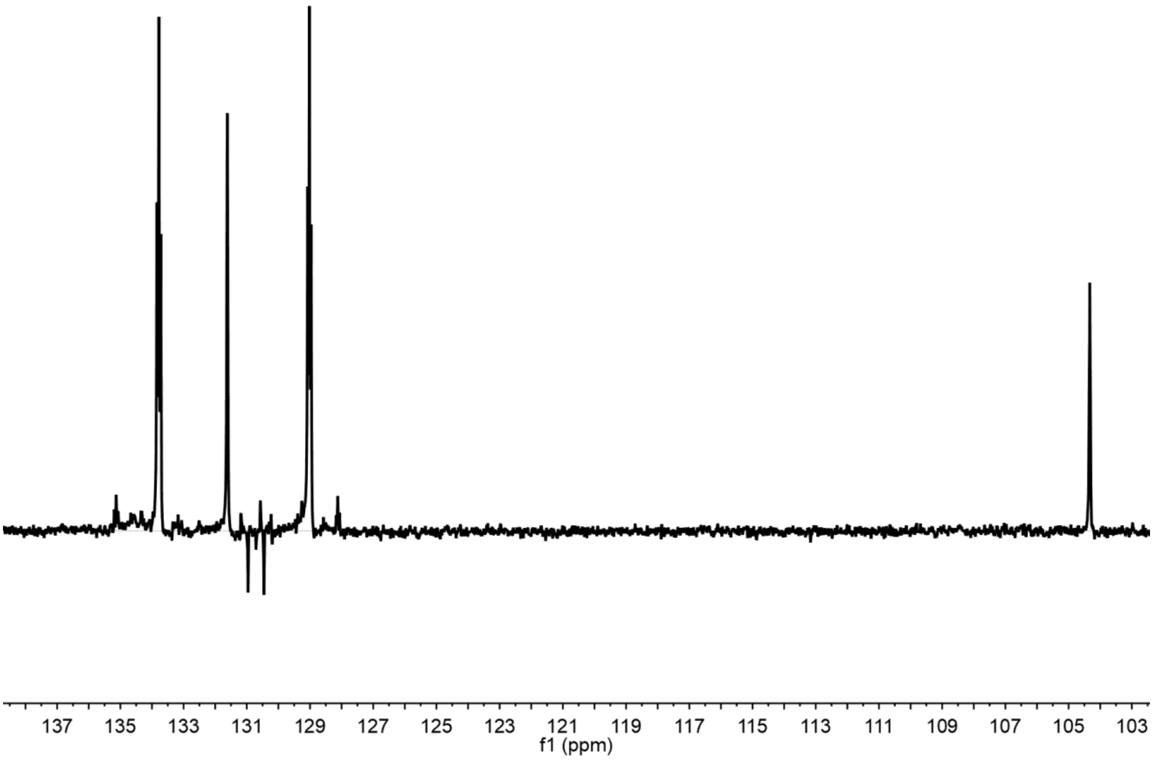

Figure S.9 – J-modulated ^{13}C NMR (101 MHz, $CDCl_3$, 25°C) spectrum of **1**

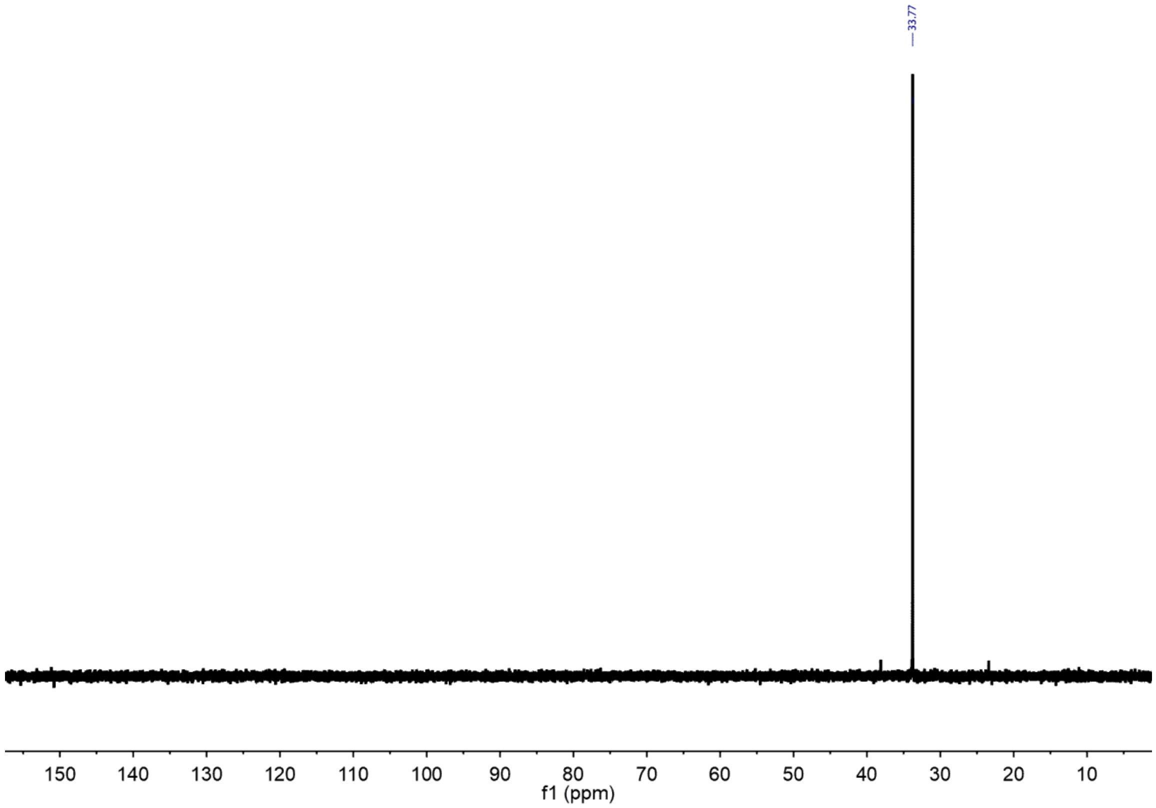

Figure S.10 – ^{31}P{^{1}H} NMR (162 MHz, $CDCl_3$, 25°C) spectrum of **1**

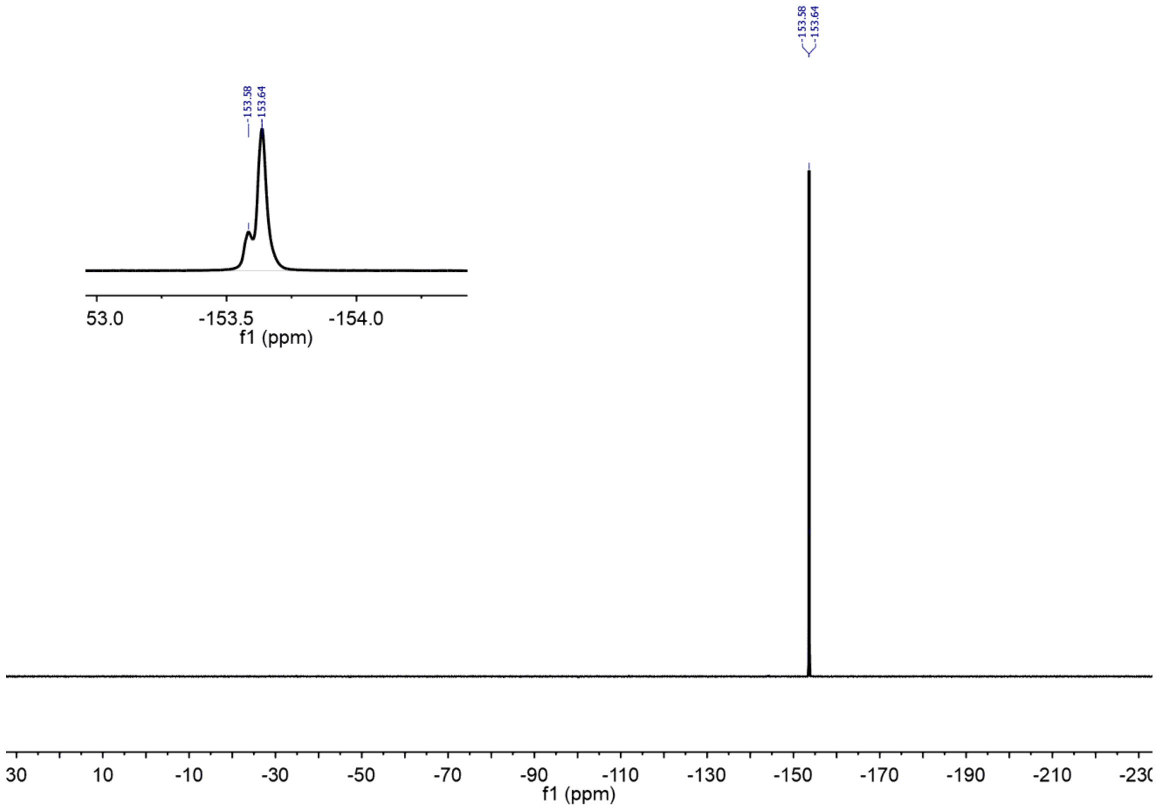

Figure S.11 – ^{19s}F NMR (376 MHz, $CDCl_3$, 25°C) spectrum of **1**

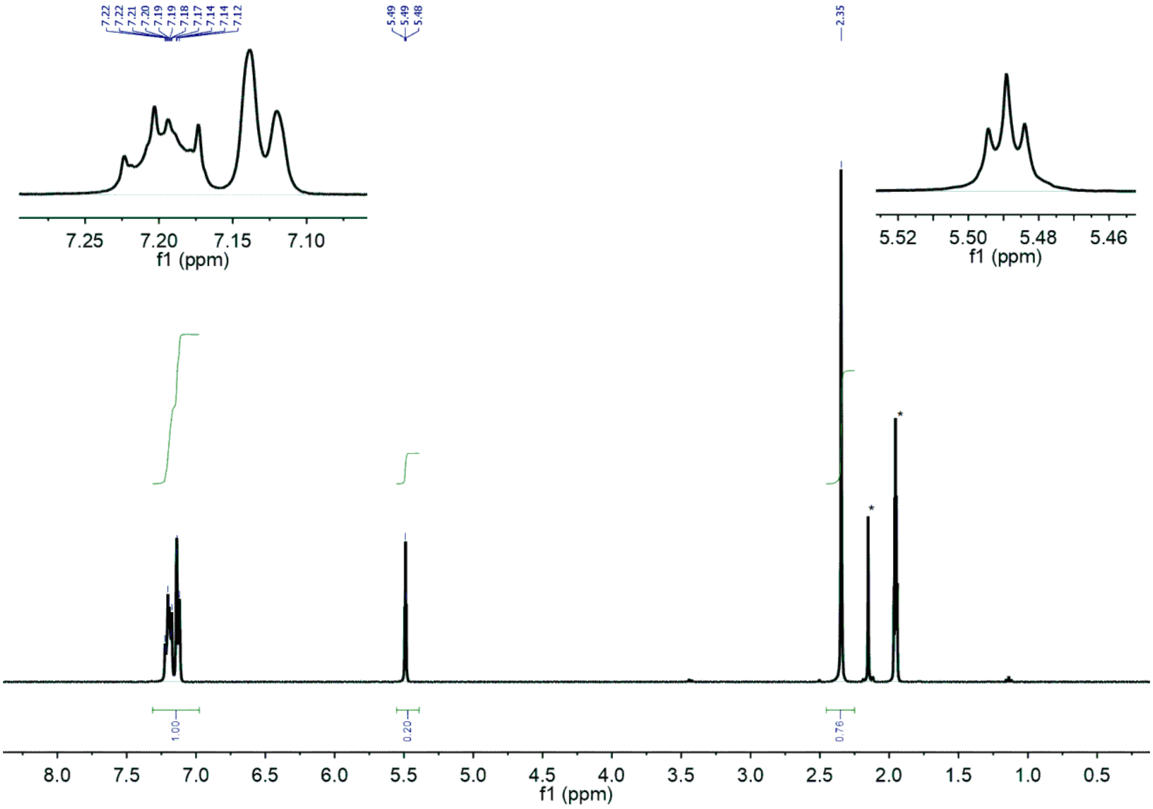

Figure S.12 – ^{1}H NMR (400 MHz, CD_3CN, 25°C) spectrum of **2**

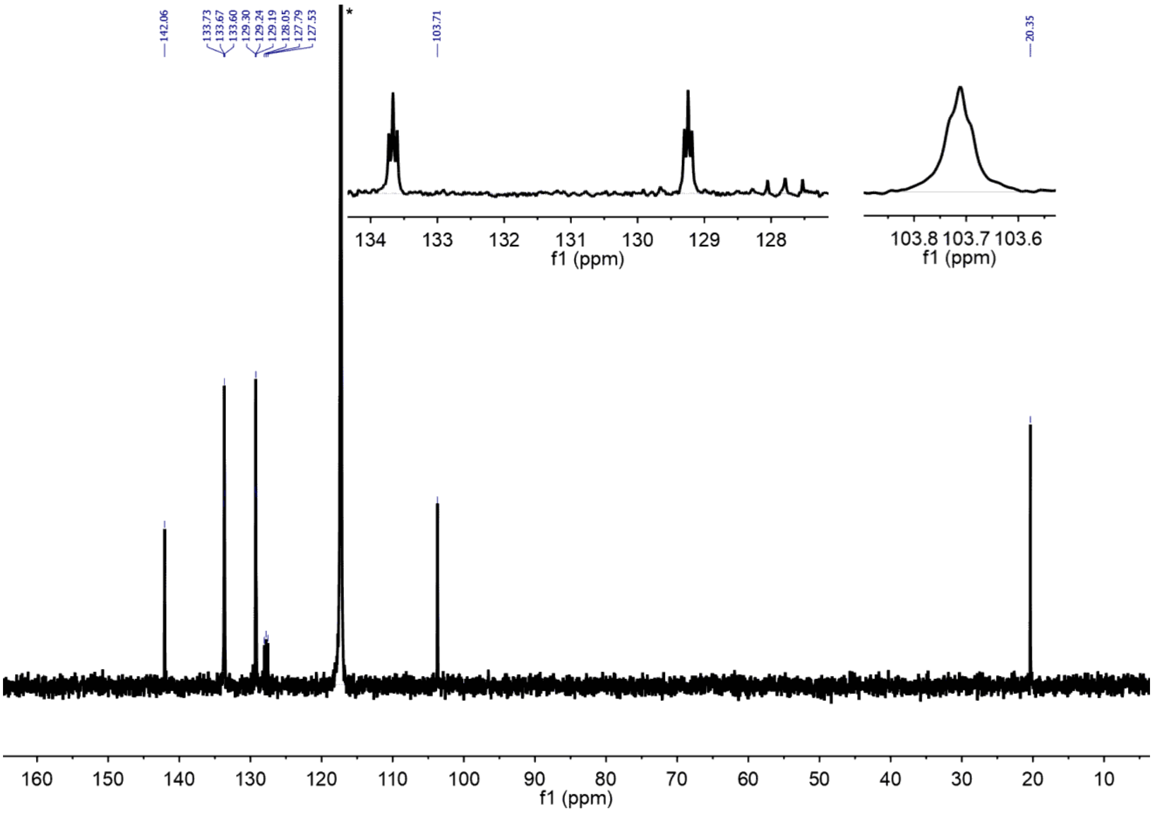

Figure S.13 – ^{13}C{^{1}H} NMR (101 MHz, CD_3CN, 25°C) spectrum of **2**

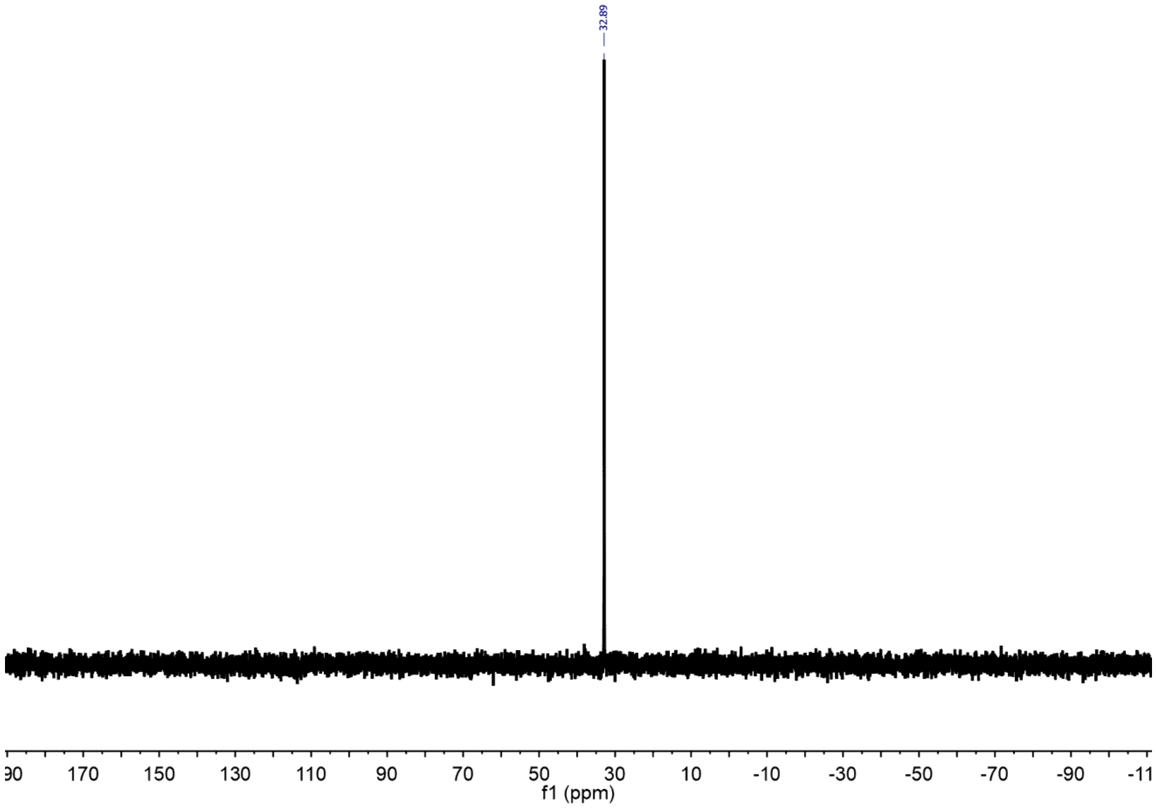

Figure S.14 – $^{31}P\{^1H\}$ NMR (162 MHz, CD_3CN, 25°C) spectrum of **2**

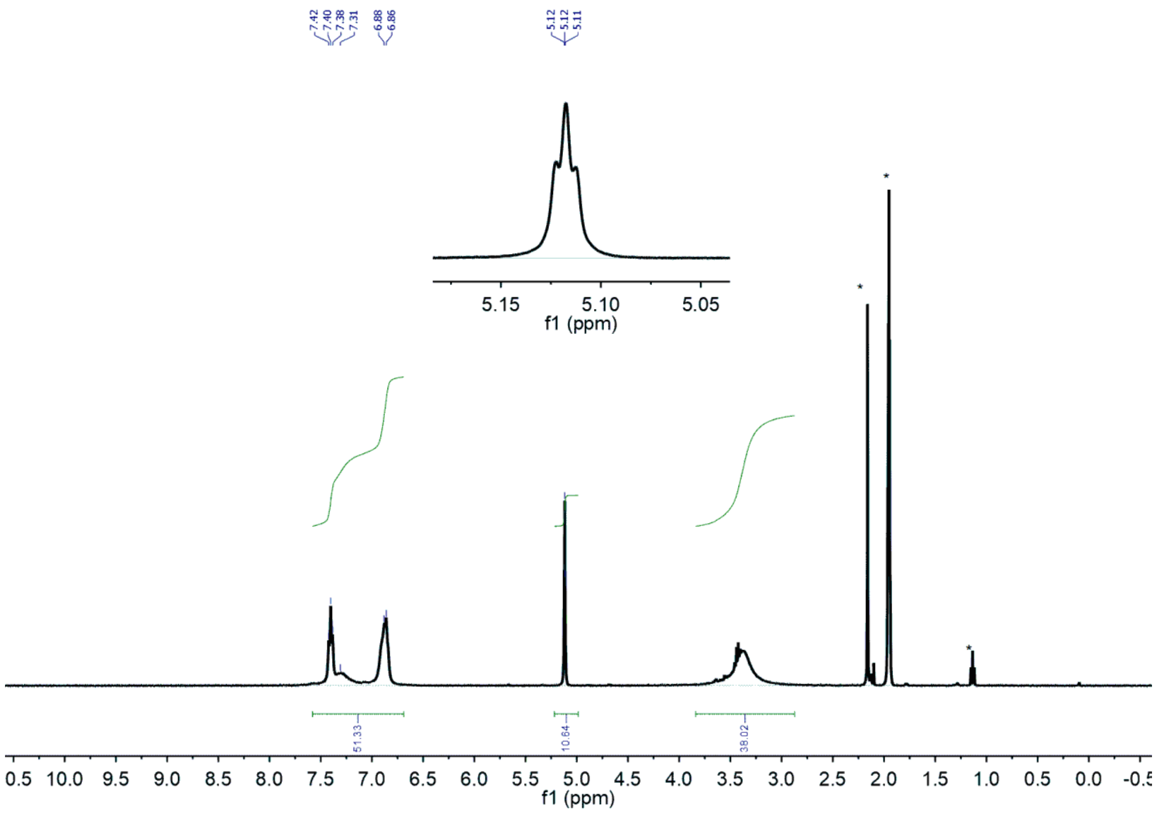

Figure S.15 – ^{1}H NMR (400 MHz, CD_3CN, 25°C) spectrum of **3**

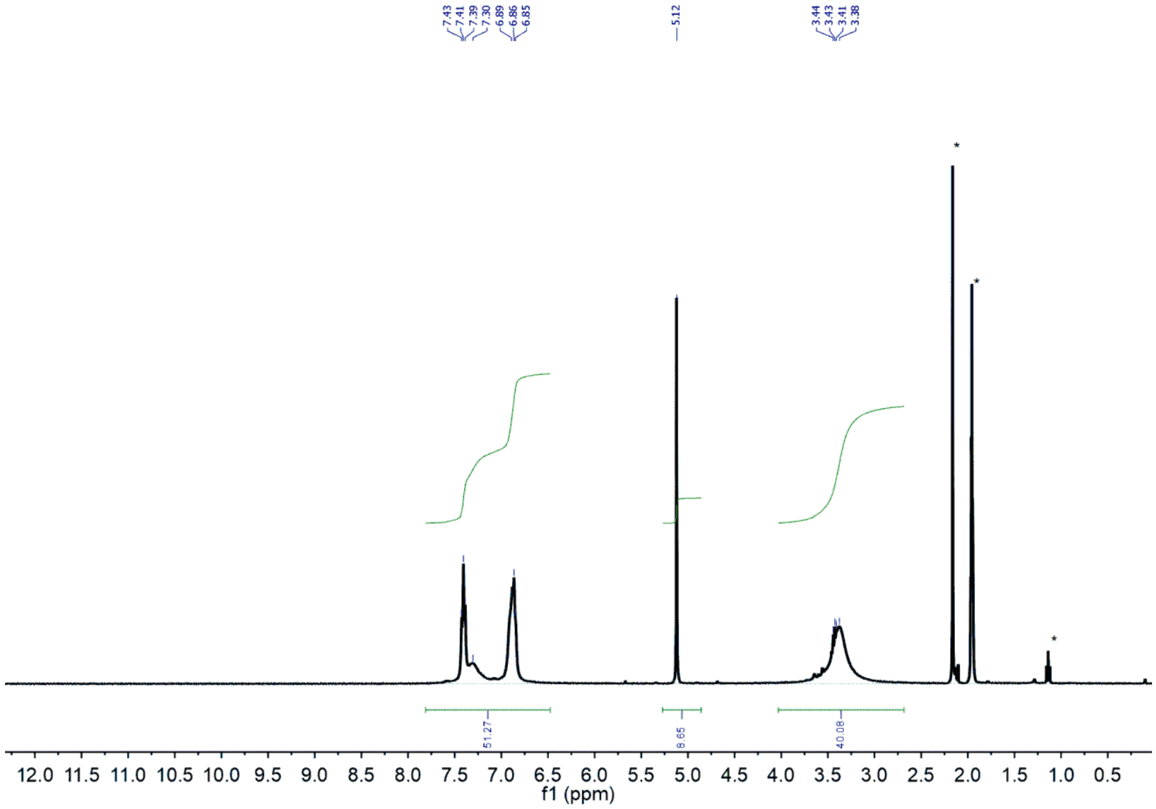

Figure S.16 – $^{1}H\{^{31}P\}$ NMR (400 MHz, CD_3CN, 25°C) spectrum of **3**

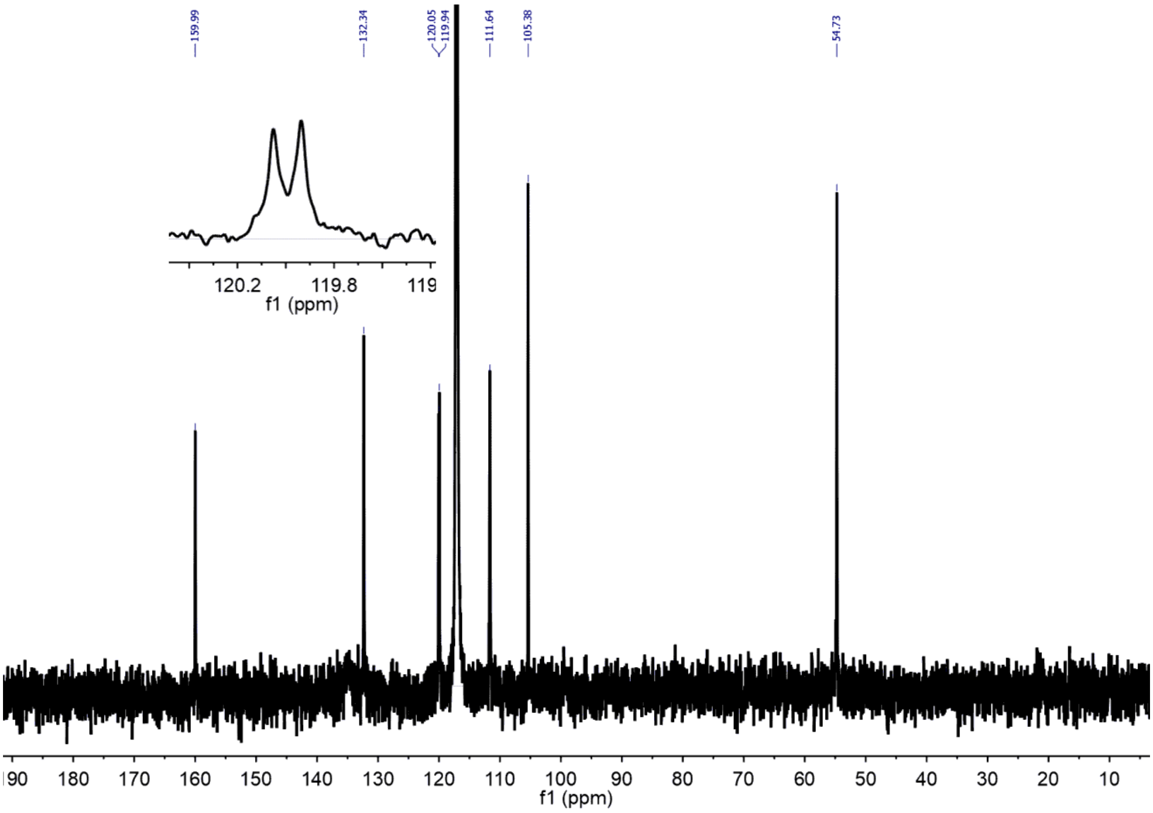

Figure S.17 – $^{13}C\{^{1}H\}$ NMR (101 MHz, CD_3CN, 25°C) spectrum of **3**

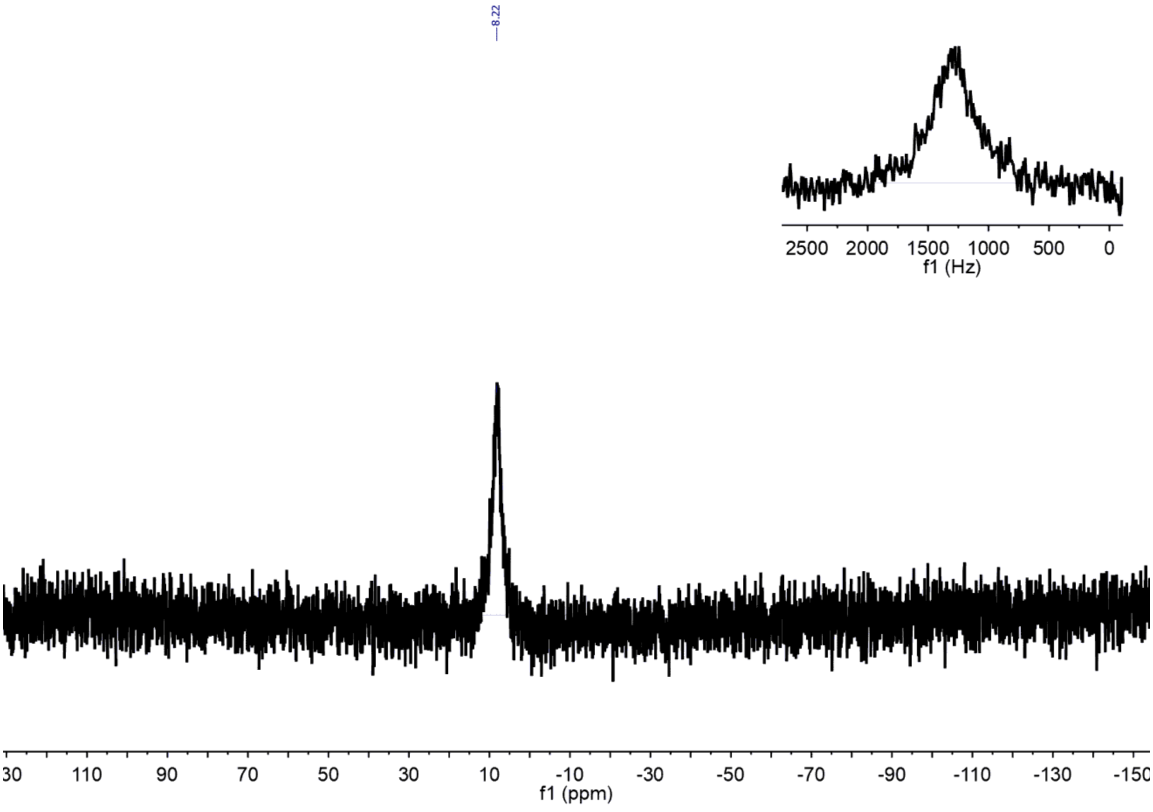

Figure S.18 – $^{31}P\{^{1}H\}$ NMR (162 MHz, CD_3CN, 25°C) spectrum of **3**

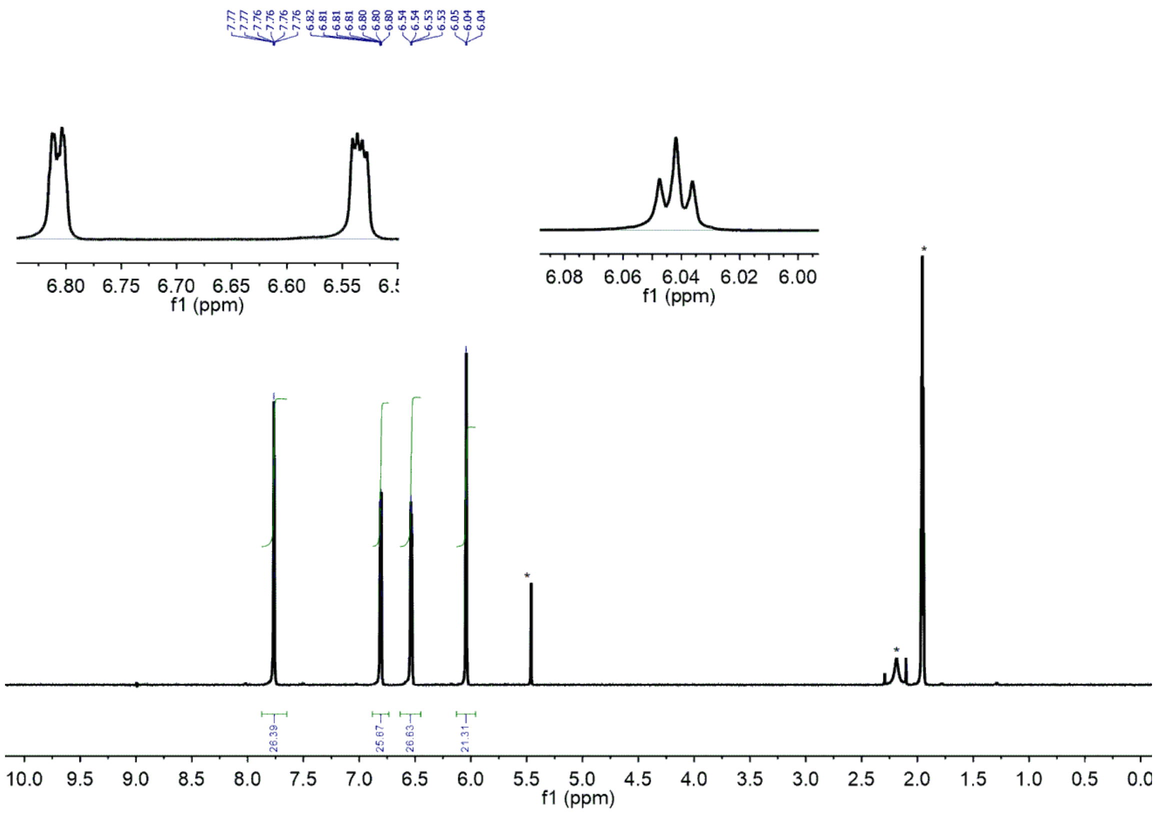

Figure S.19 – ^{1}H NMR (400 MHz, CD_3CN, 25°C) spectrum of **4**

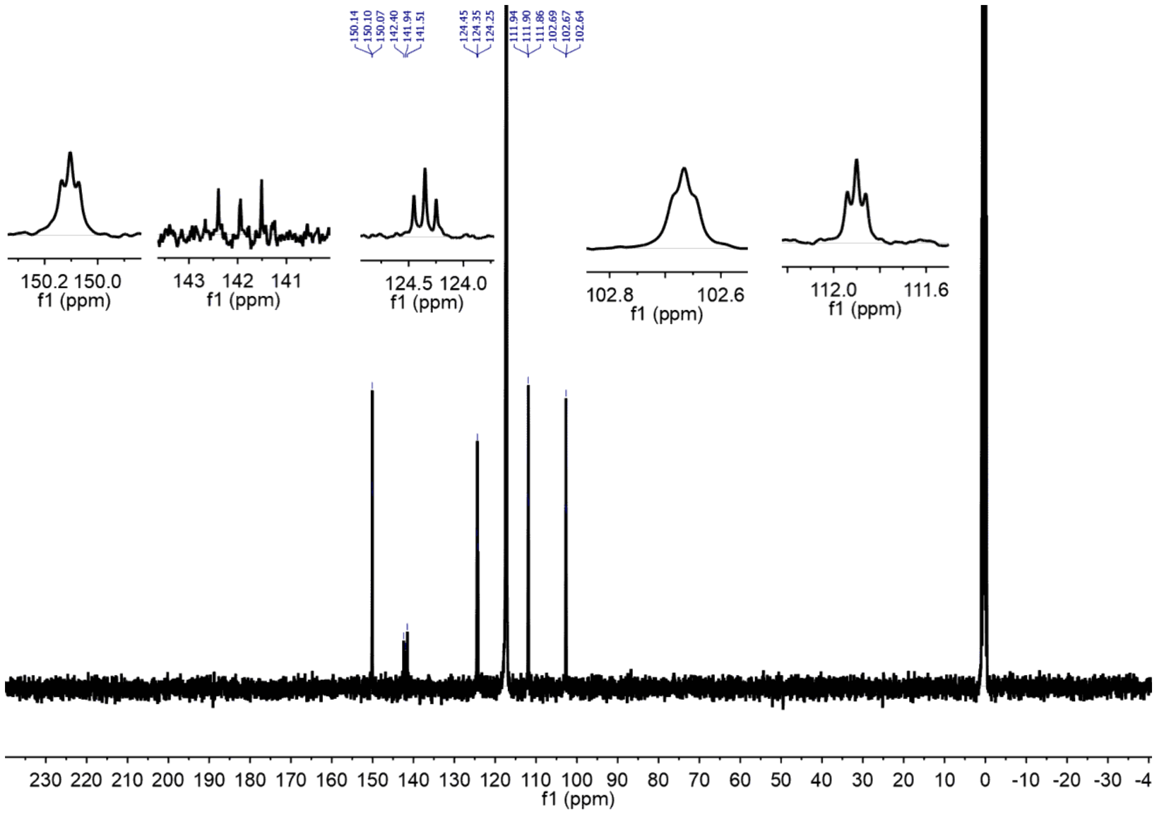

Figure S.20 – $^{13}C\{^{1}H\}$ NMR (101 MHz, CD_3CN, 25°C) spectrum of **4**

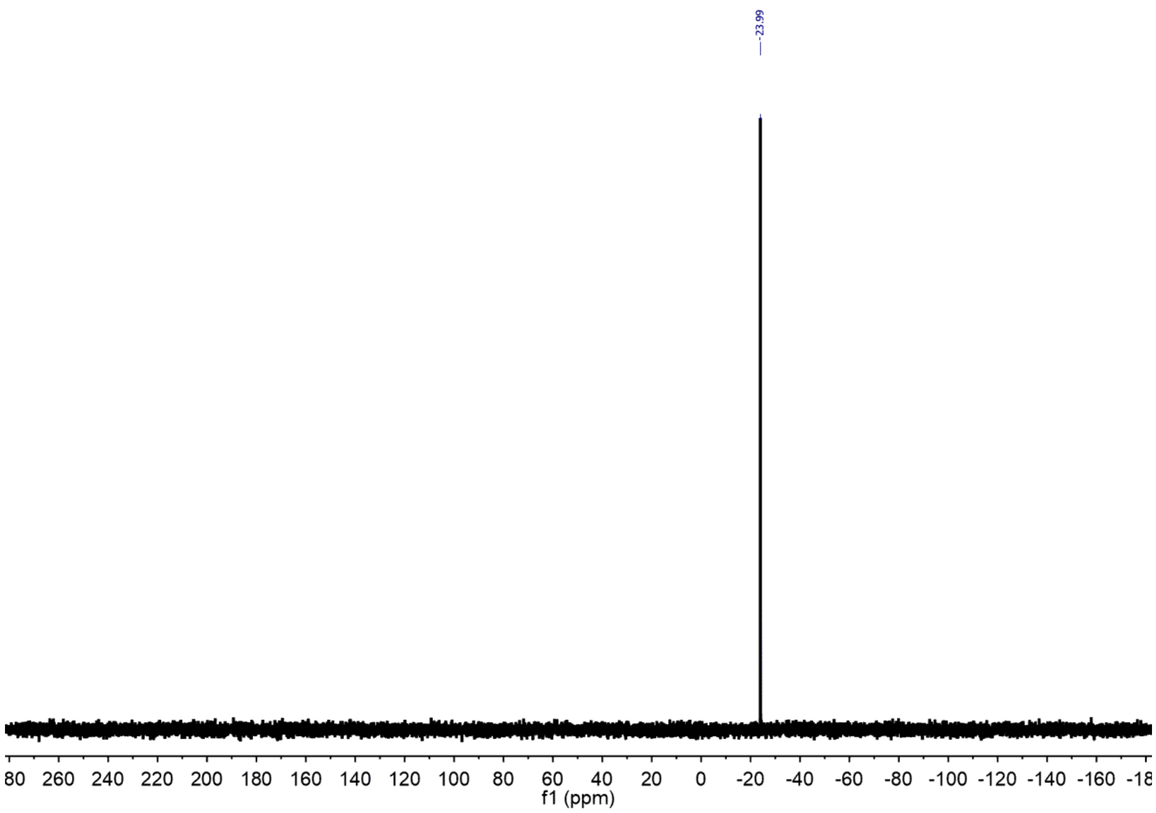

Figure S.21 – $^{31}P\{^1H\}$ NMR (162 MHz, CD_3CN, 25°C) spectrum of **4**

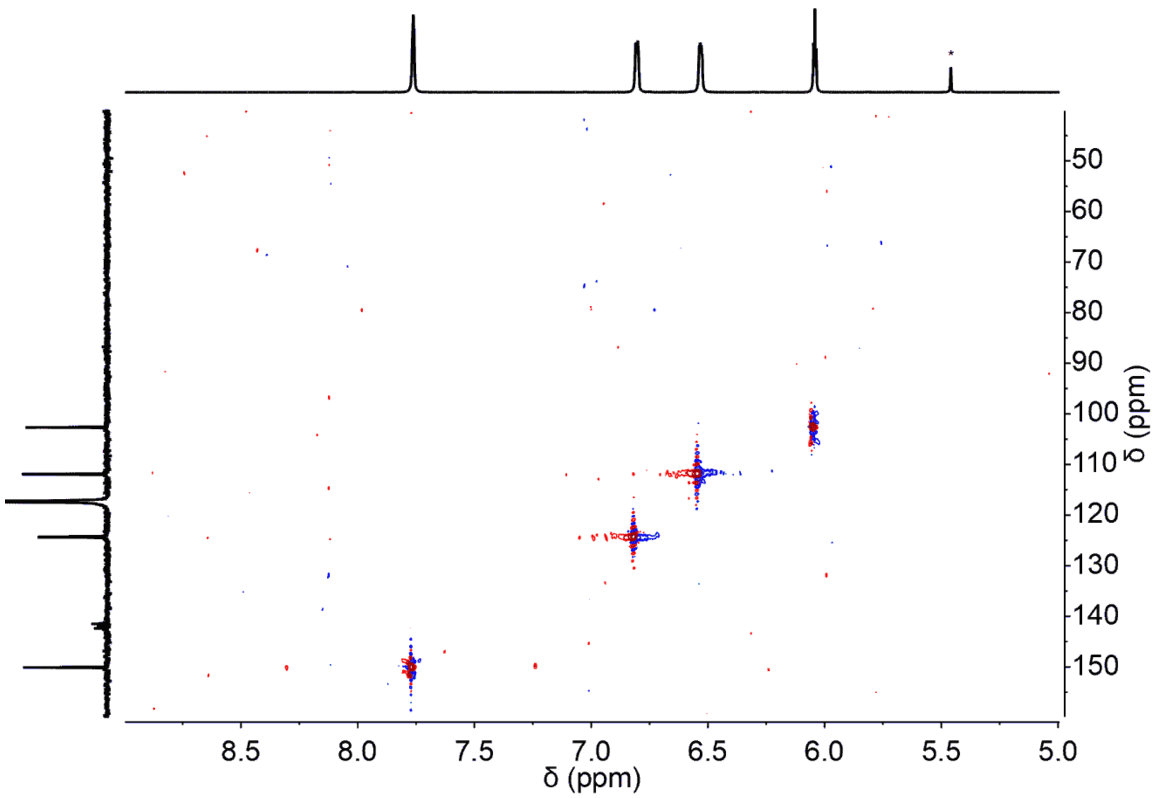

Figure S.22 – ^1H–^{13}C HSQC NMR (400 MHz, CD_3CN, 25°C) spectrum of **4**

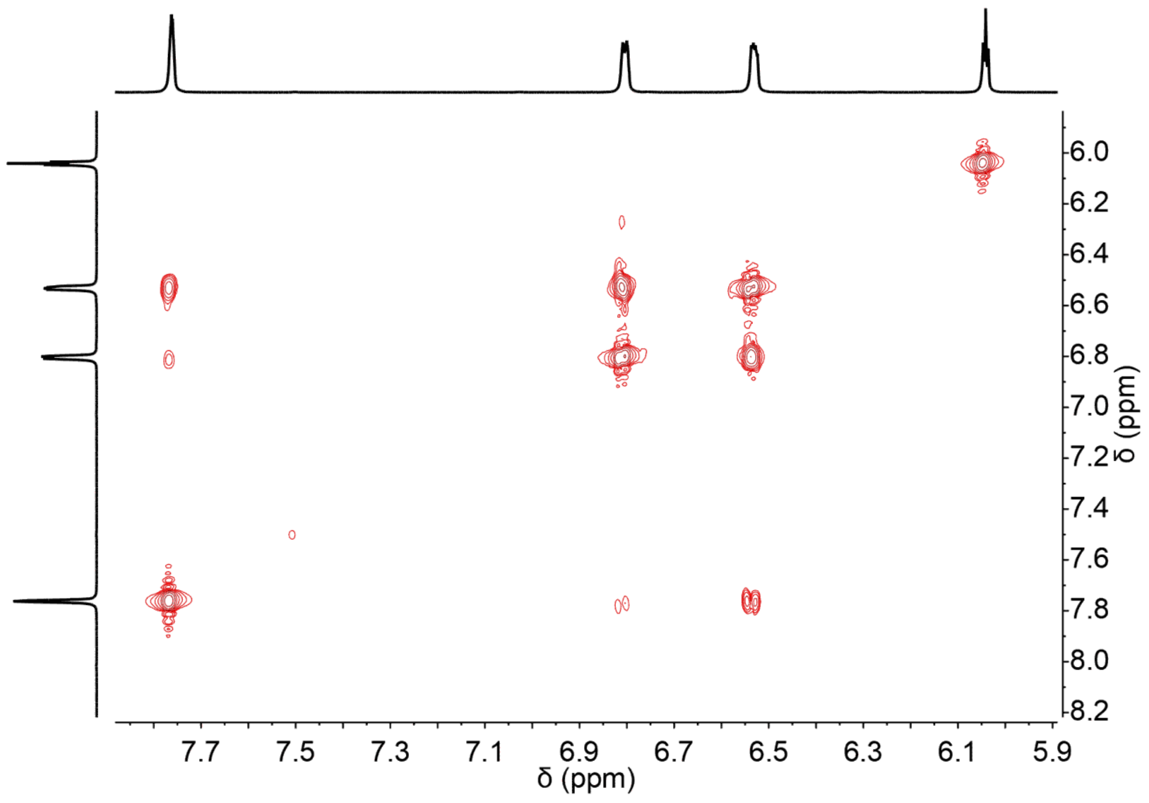

Figure S.23 – ^{1}H–^{1}H COSY NMR (400 MHz, CD_3CN, 25°C) spectrum of **4**

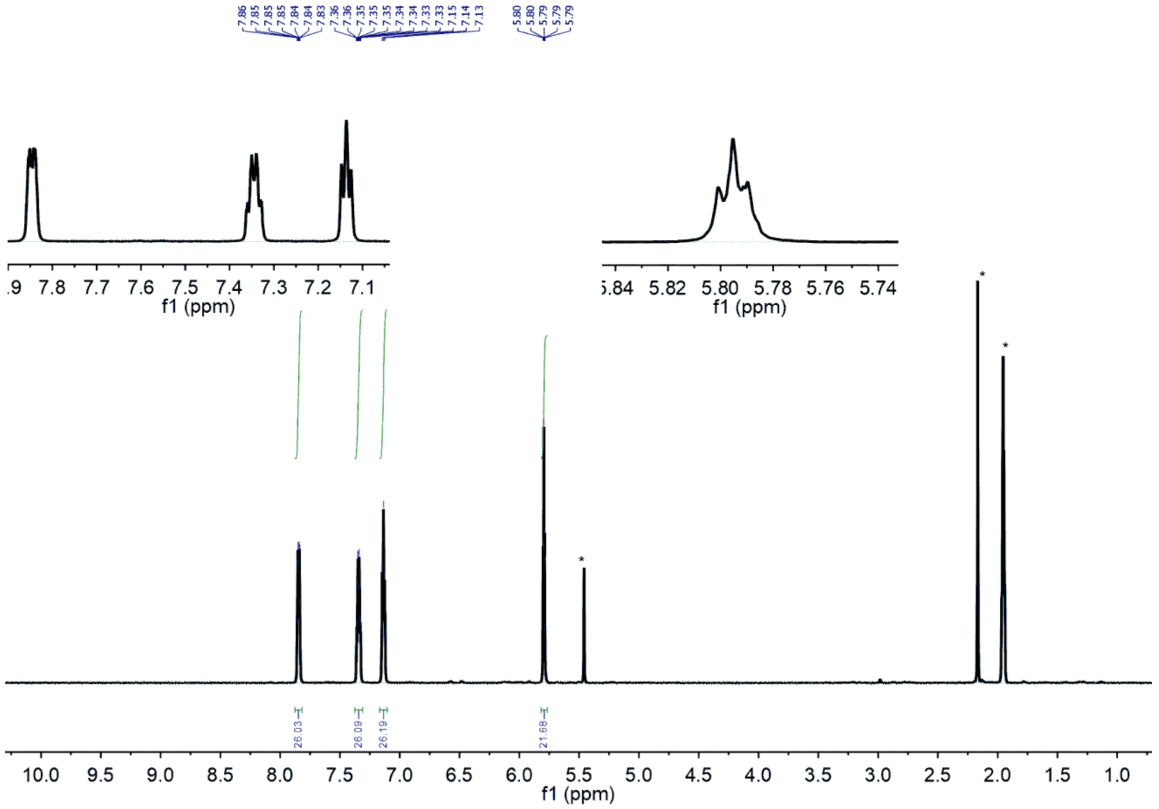

Figure S.24 – ^{1}H NMR (400 MHz, CD_3CN, 25°C) spectrum of **5**

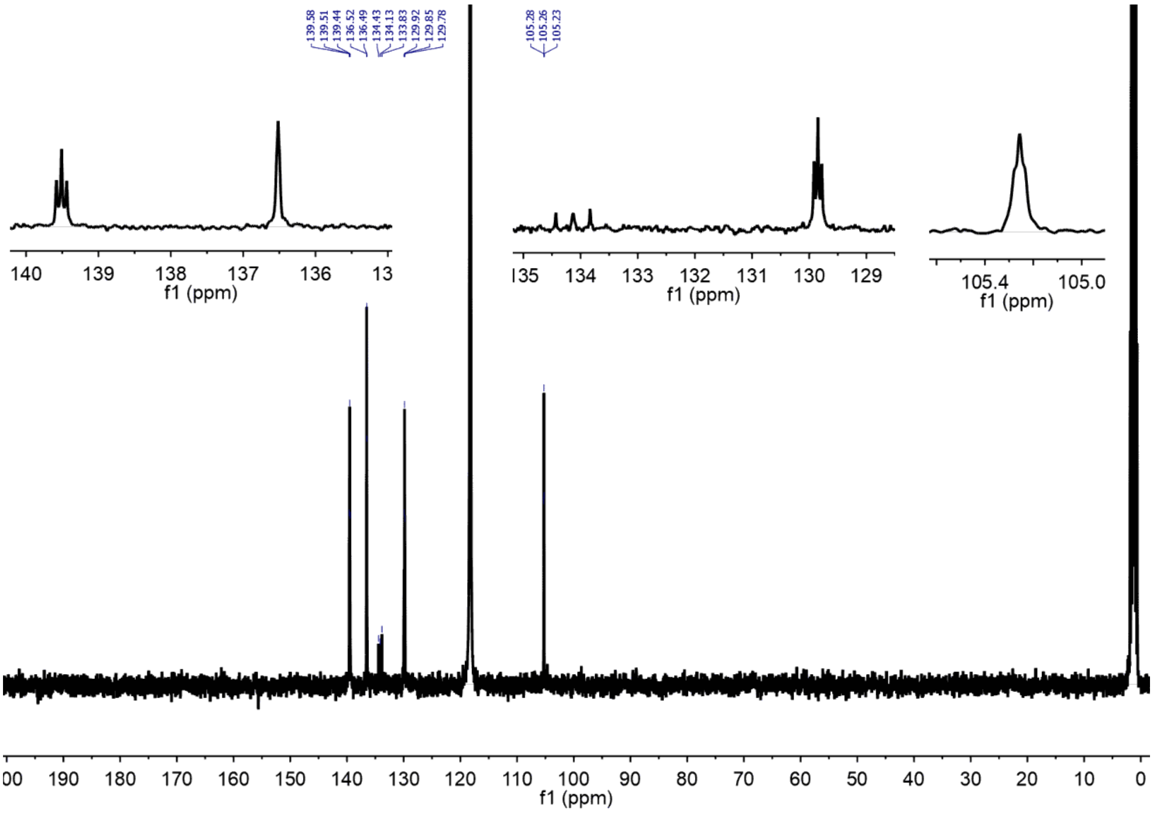

Figure S.25 – $^{13}C\{^{1}H\}$ NMR (101 MHz, CD_3CN, 25°C) spectrum of **5**

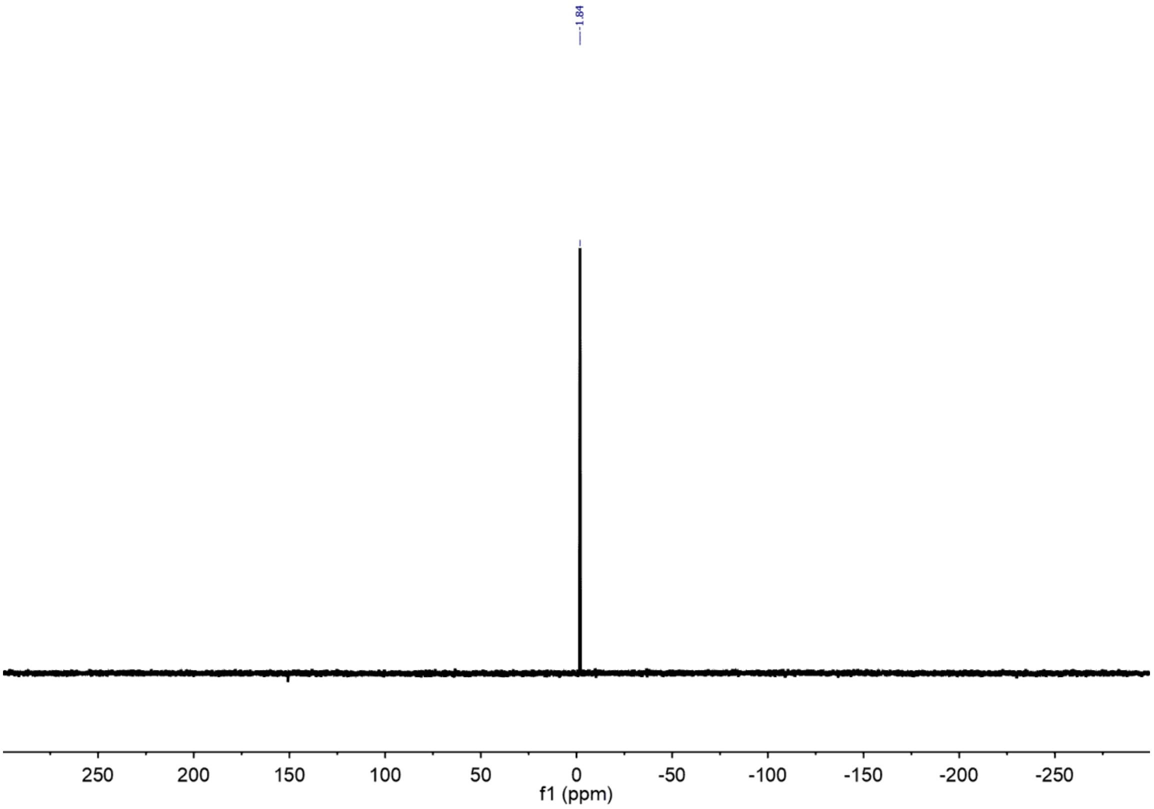

Figure S.26 – $^{31}P\{^{1}H\}$ NMR (162 MHz, CD_3CN, 25°C) spectrum of **5**

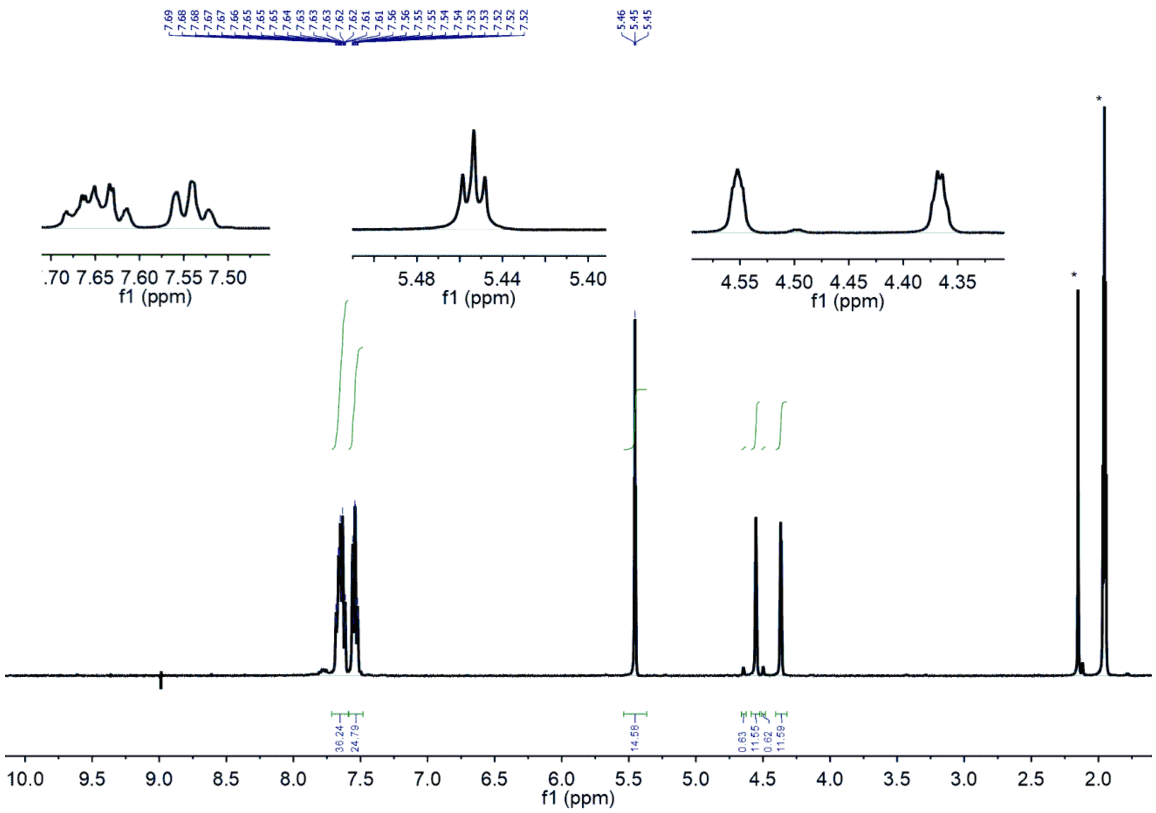

Figure S.27 – ^{1}H NMR (400 MHz, CD_3CN, 25°C) spectrum of **6**

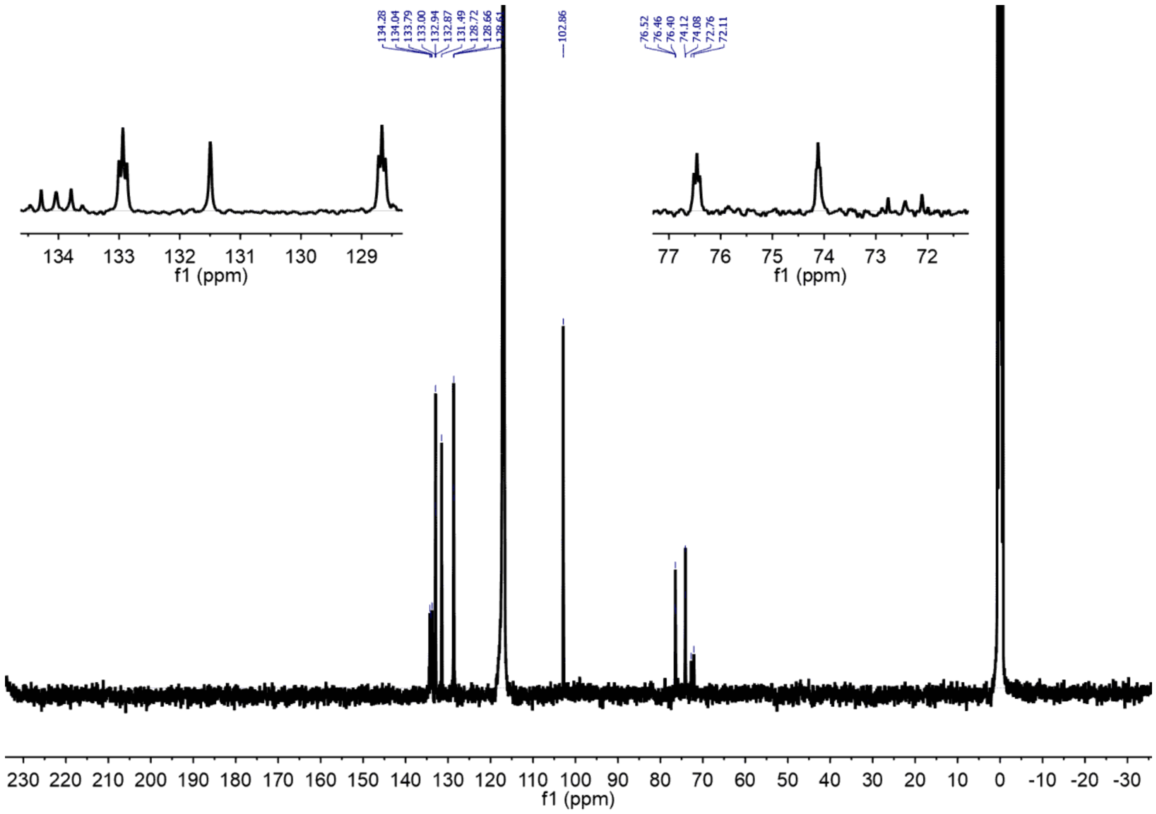

Figure S.28 – $^{13}C\{^{1}H\}$ NMR (101 MHz, CD_3CN, 25°C) spectrum of **6**

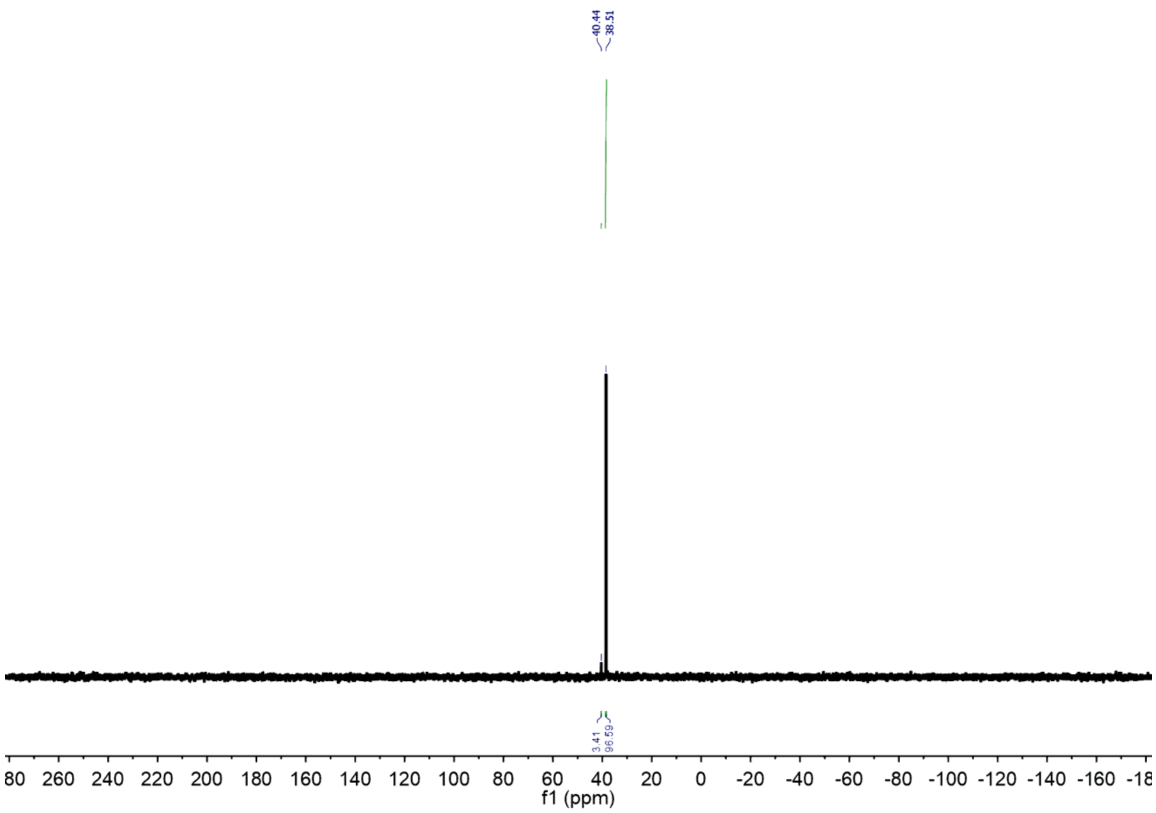

Figure S.29 – $^{31}P\{^{1}H\}$ NMR (162 MHz, CD_3CN, 25°C) spectrum of **6**

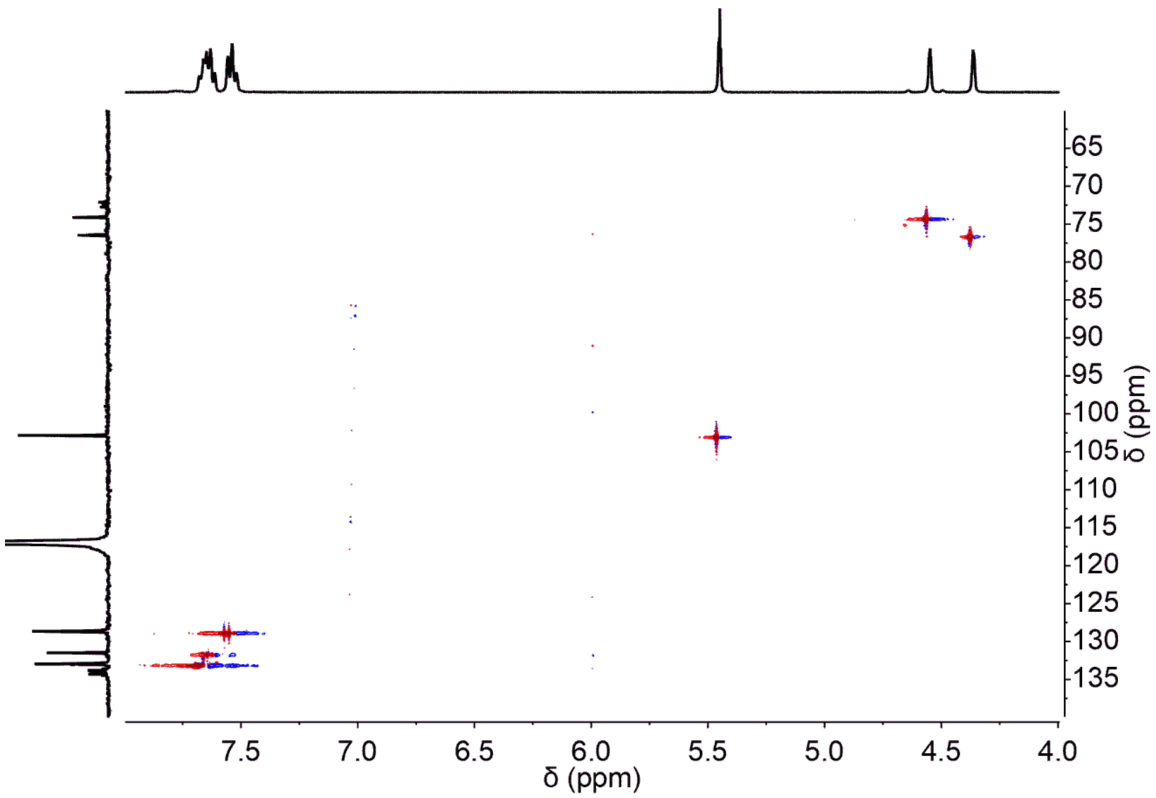

Figure S.30 – ^{1}H–^{13}C HSQC NMR (400 MHz, CD_3CN, 25°C) spectrum of **6**

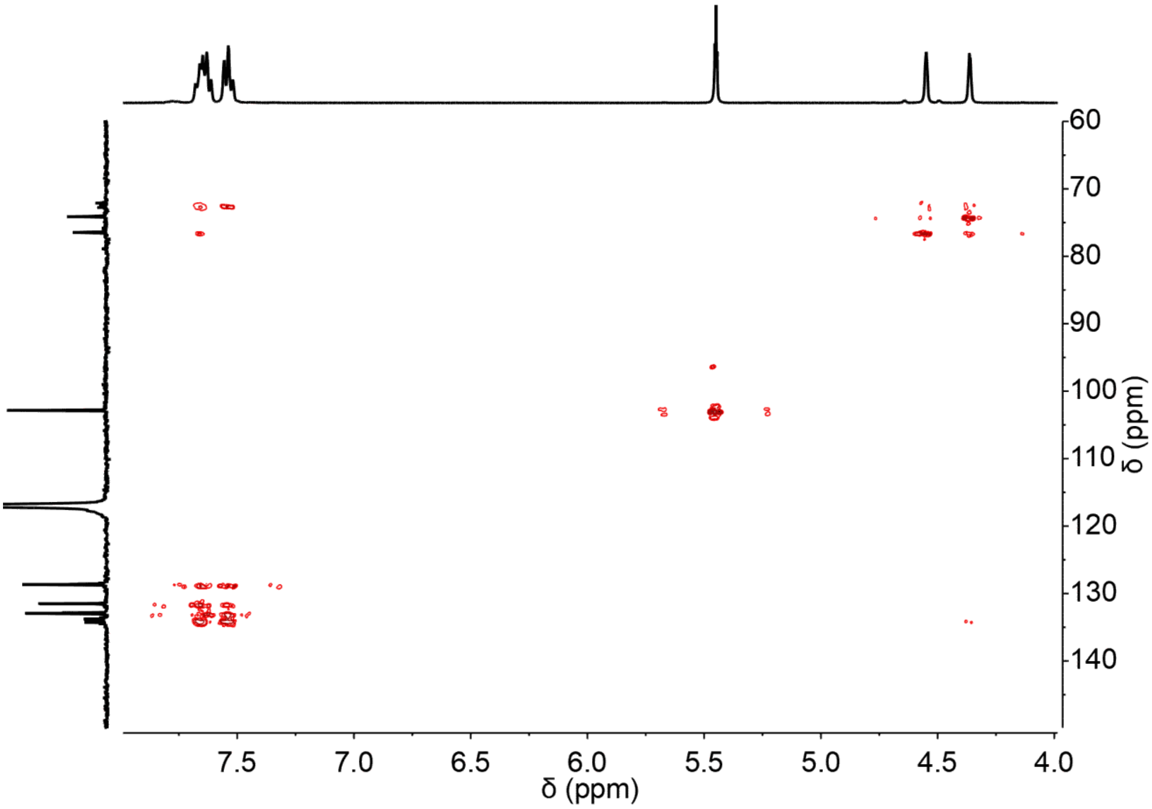

Figure S.31 – ^{1}H–^{13}C HMBC NMR (400 MHz, CD_3CN, 25°C) spectrum of **6**

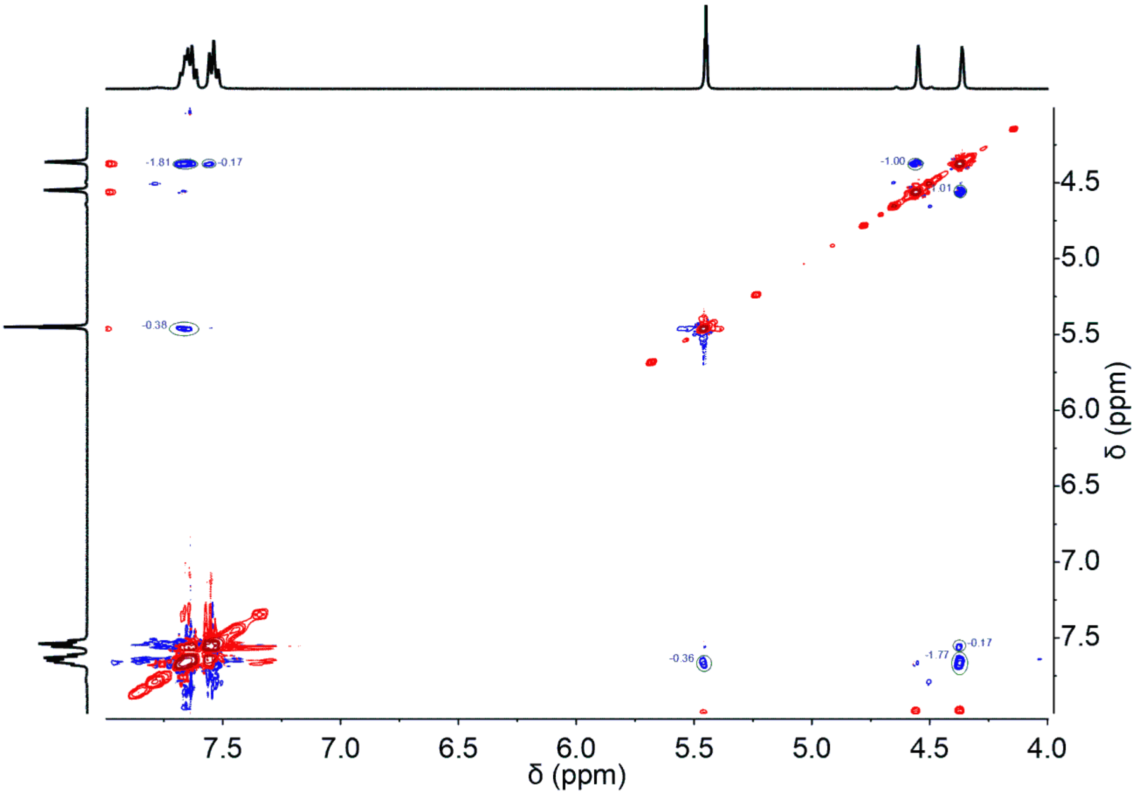

Figure S.32 – ^{1}H–^{1}H NOESY NMR (400 MHz, CD_3CN, 25°C) spectrum of **6**

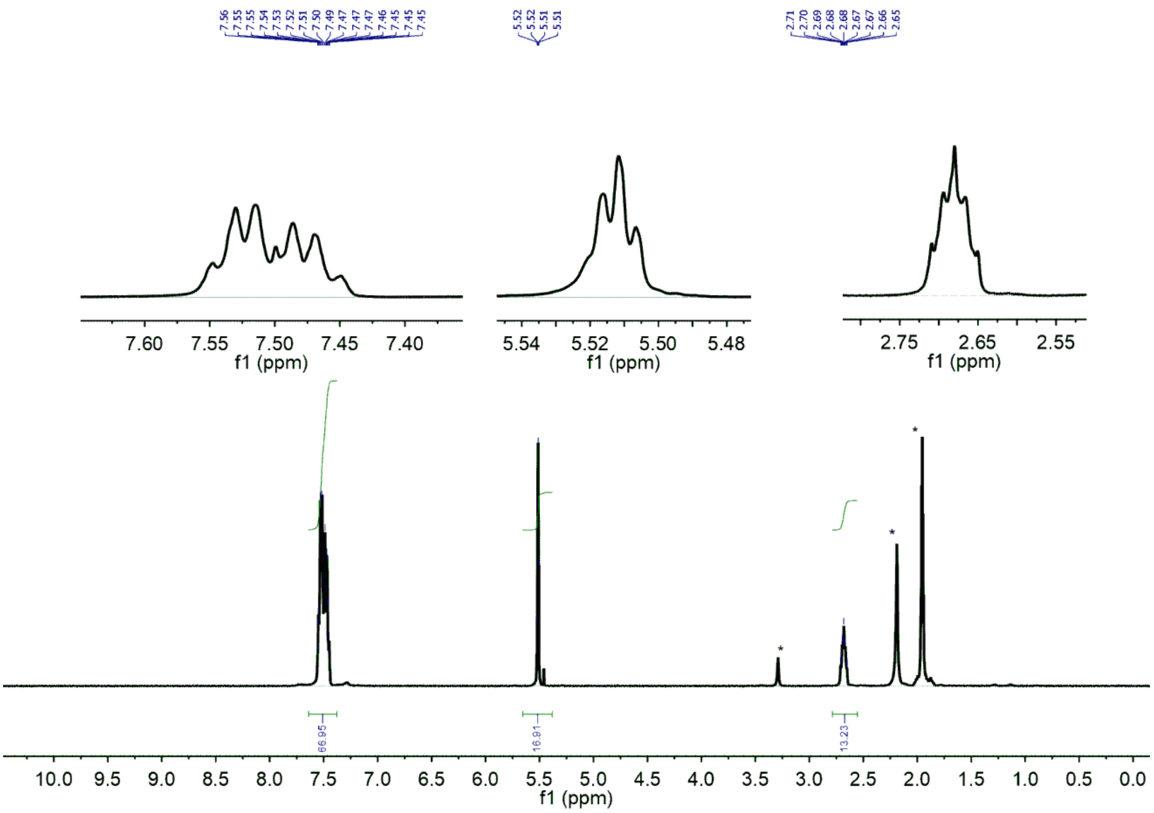

Figure S.33 – ^{1}H NMR (400 MHz, CD_3CN, 25°C) spectrum of **7**

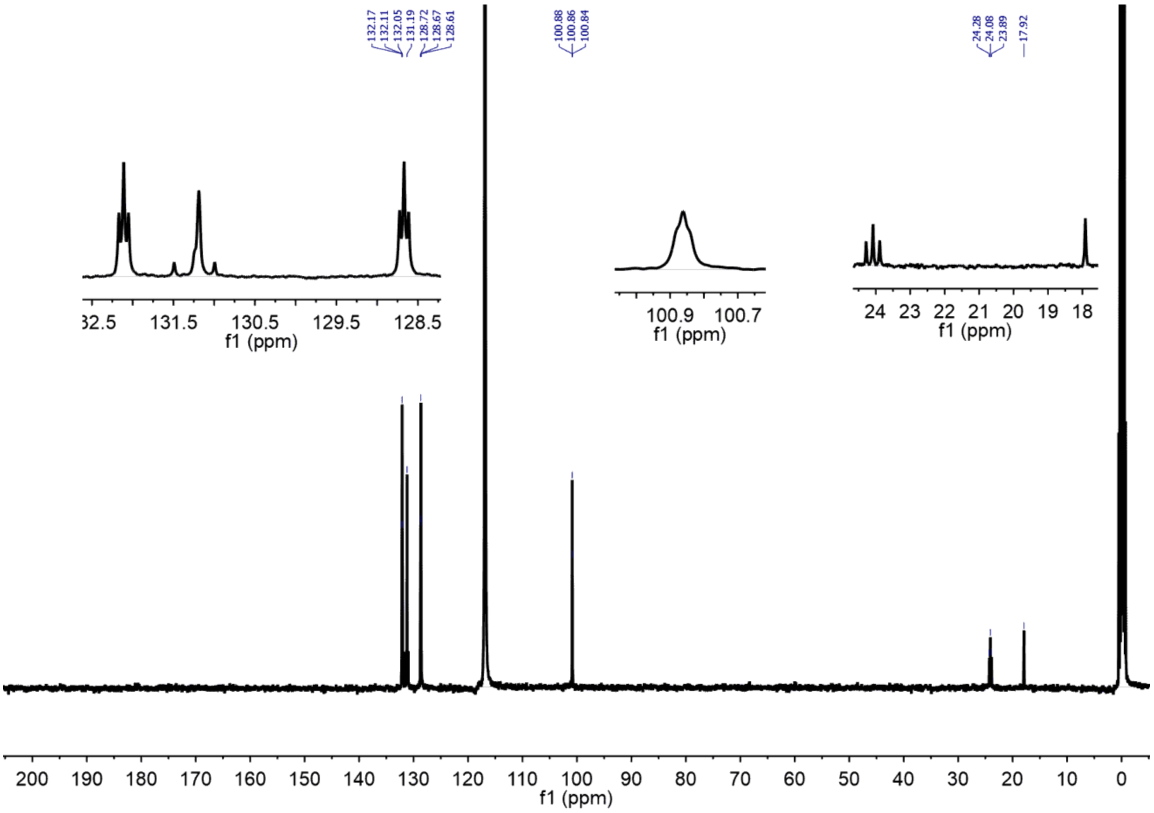

Figure S.34 – $^{13}C\{^{1}H\}$ NMR (101 MHz, CD_3CN, 25°C) spectrum of **7**

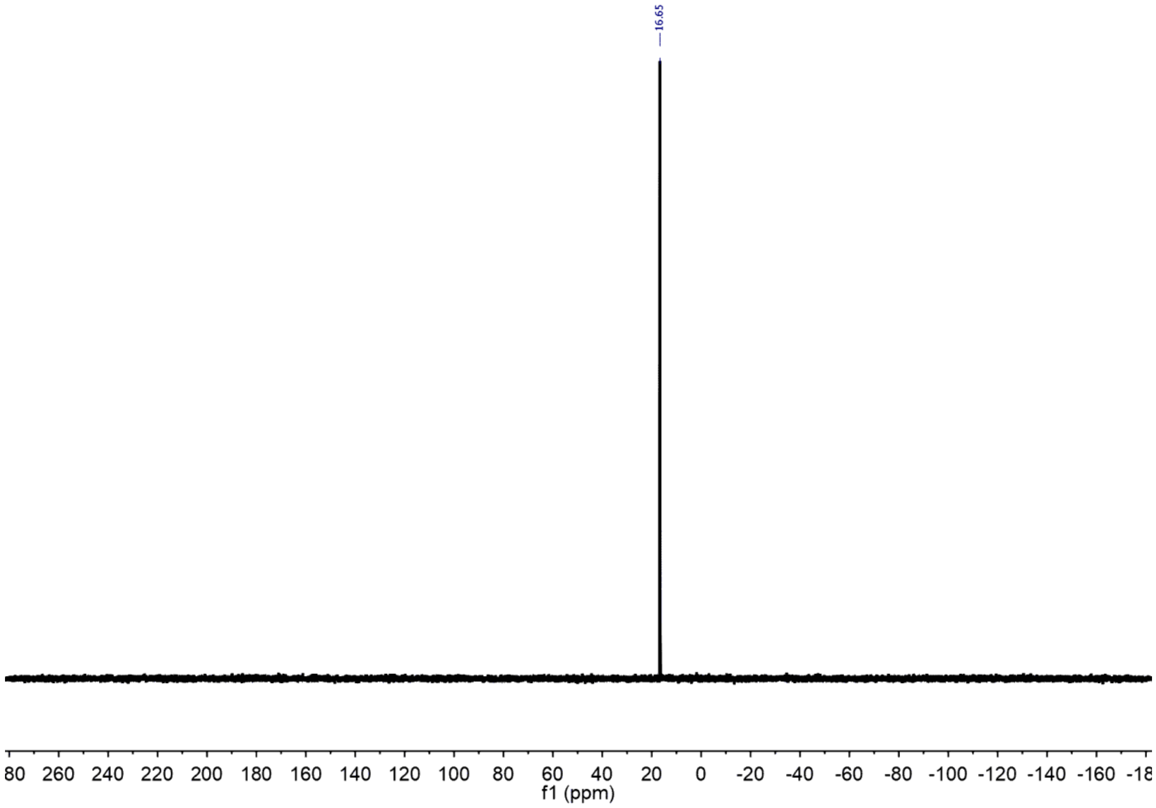

Figure S.35 – $^{31}P\{^{1}H\}$ NMR (162 MHz, CD_3CN, 25°C) spectrum of **7**

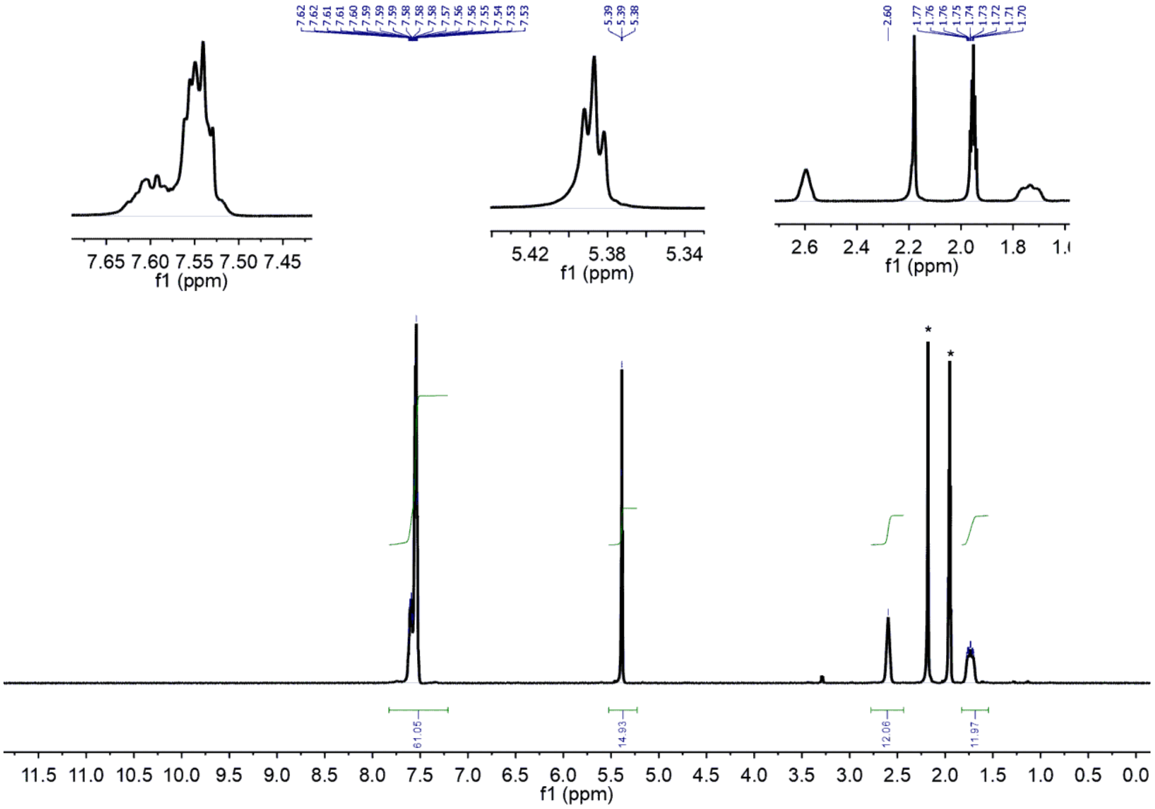

Figure S.36 – ^{1}H NMR (400 MHz, CD_3CN, 25°C) spectrum of **8**

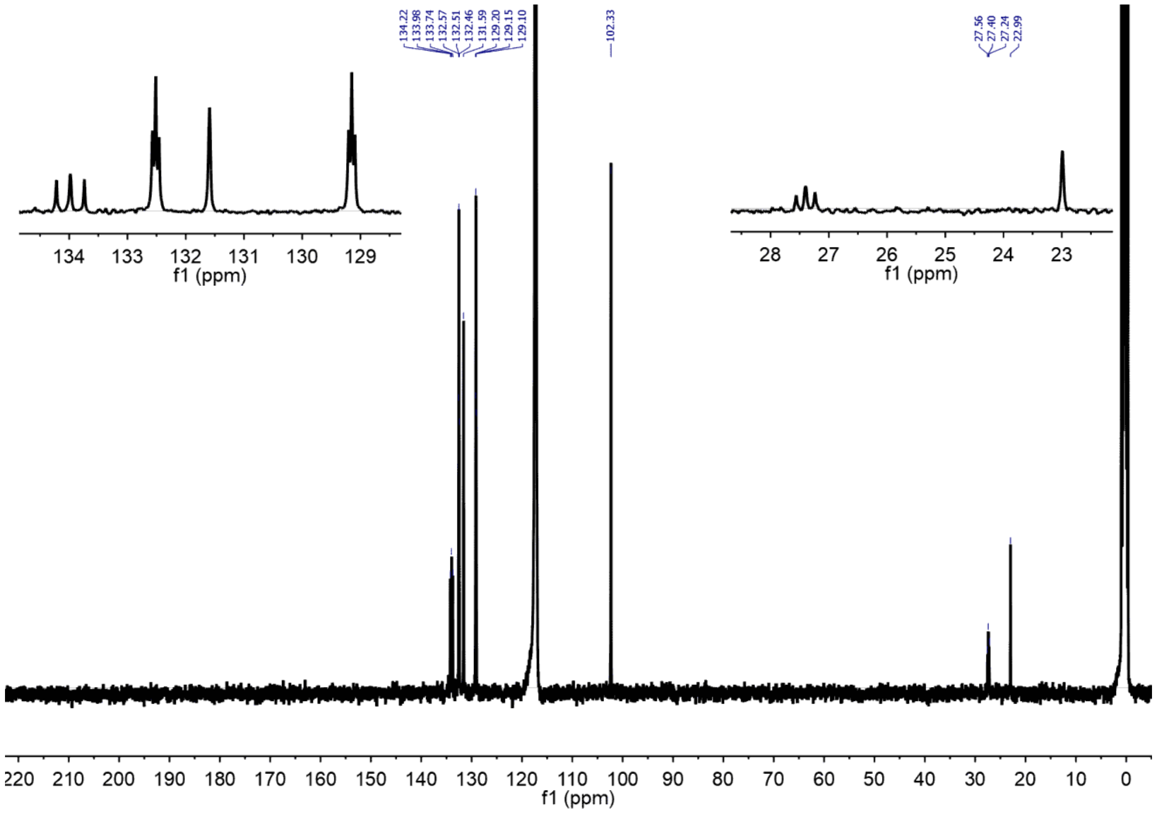

Figure S.37 – $^{13}C\{^1H\}$ NMR (101 MHz, CD_3CN, 25°C) spectrum of **8**

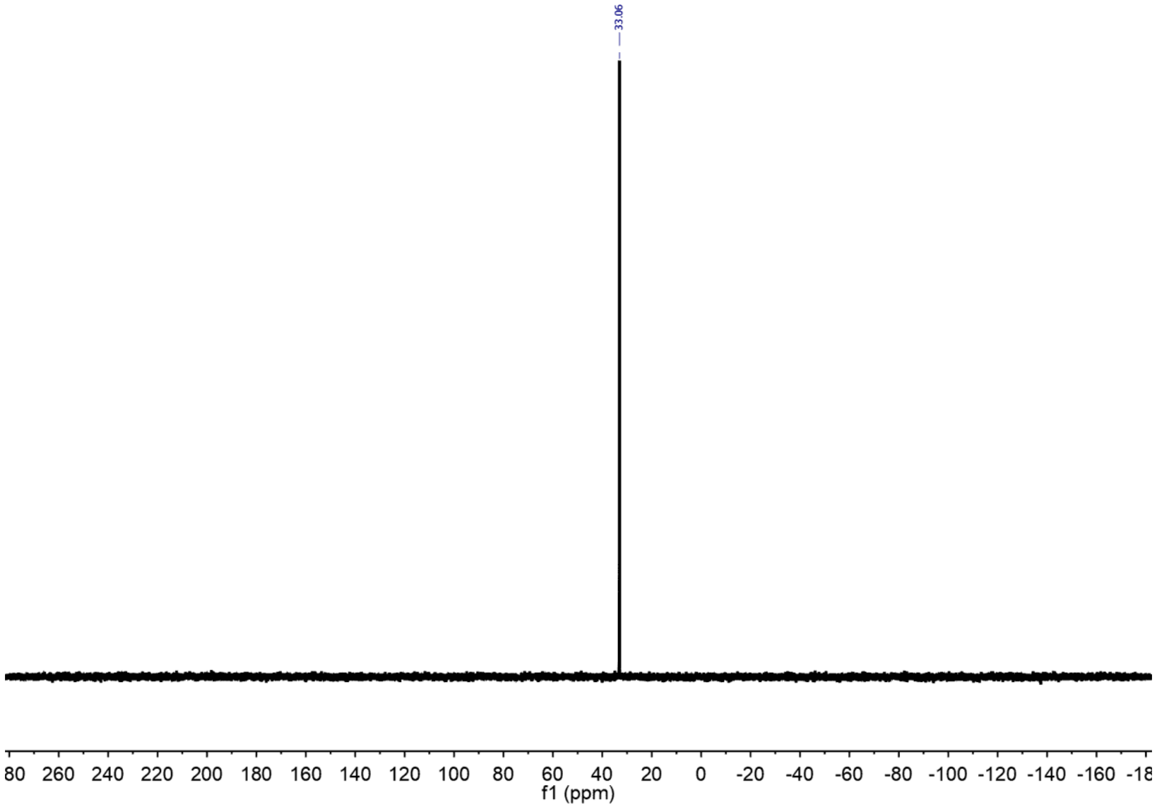

Figure S.38 – $^{31}P\{^1H\}$ NMR (162 MHz, CD_3CN, 25°C) spectrum of **8**

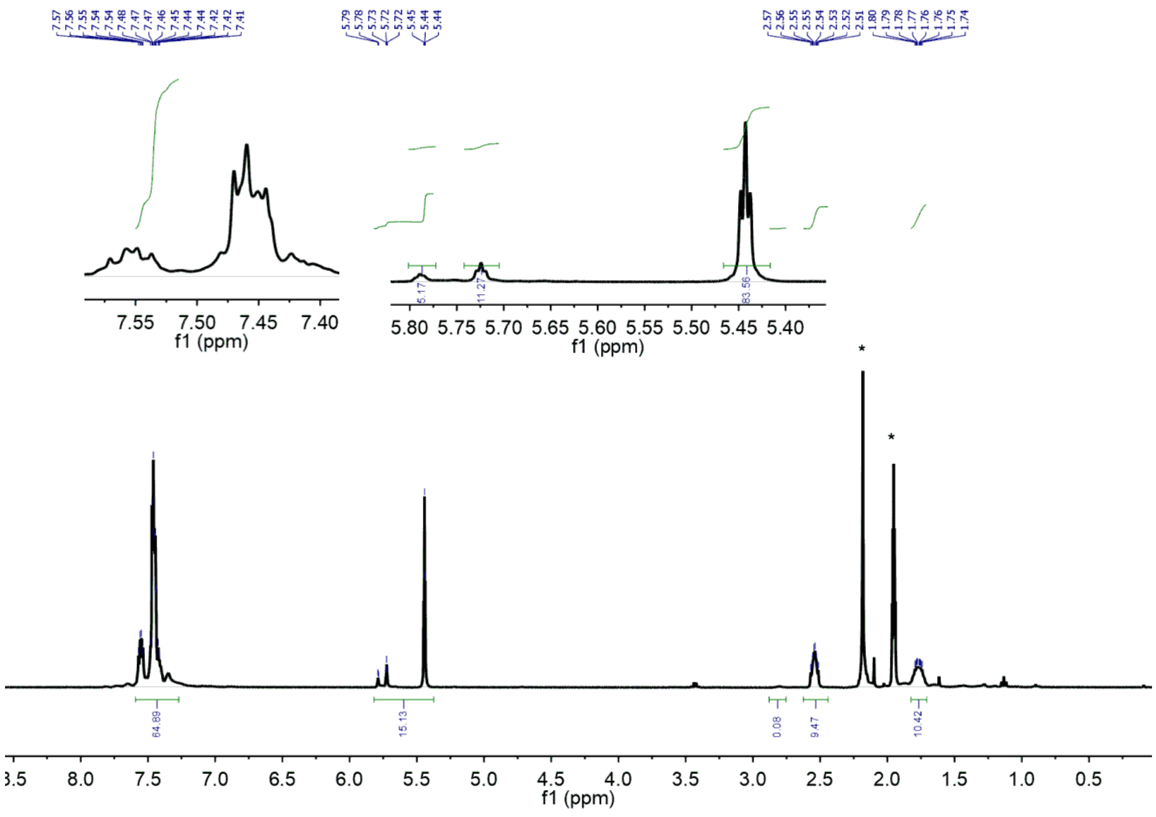

Figure S.39 – ^{1}H NMR (400 MHz, CD_3CN, 25°C) spectrum of **9**

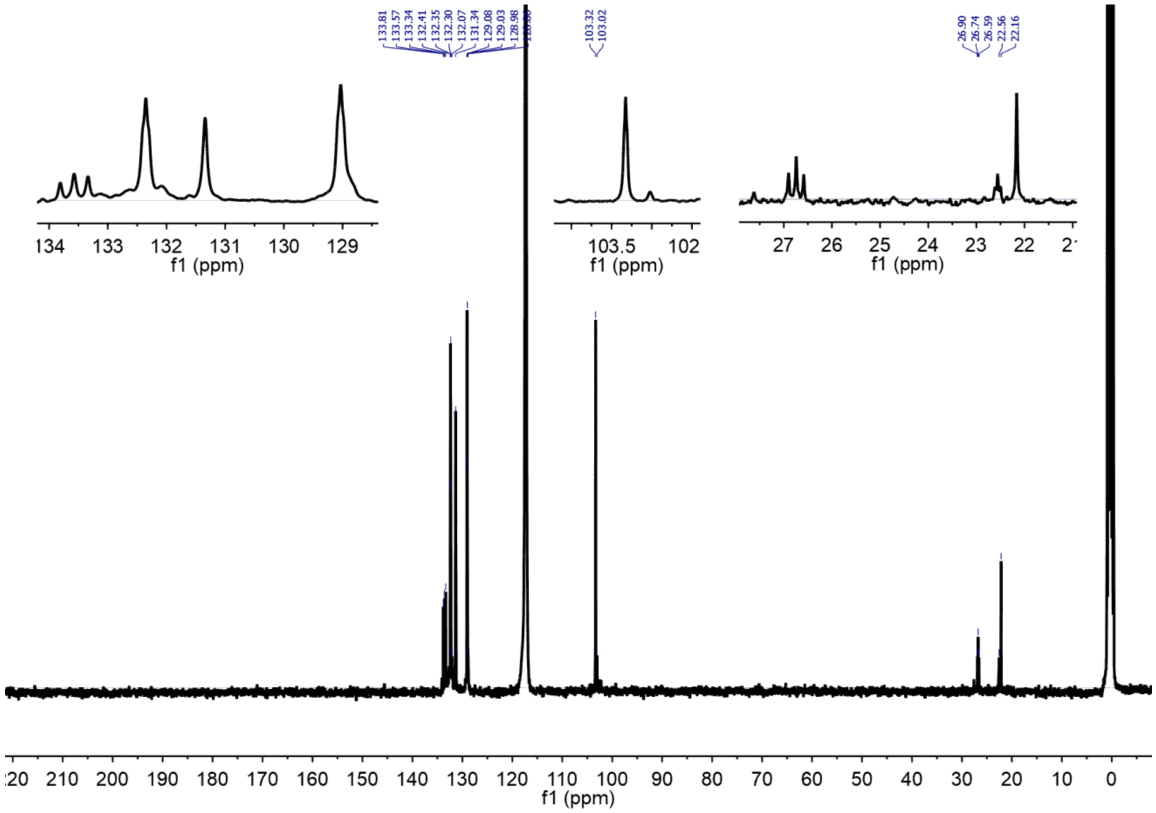

Figure S.40 – $^{13}C\{^{1}H\}$ NMR (101 MHz, CD_3CN, 25°C) spectrum of **9**

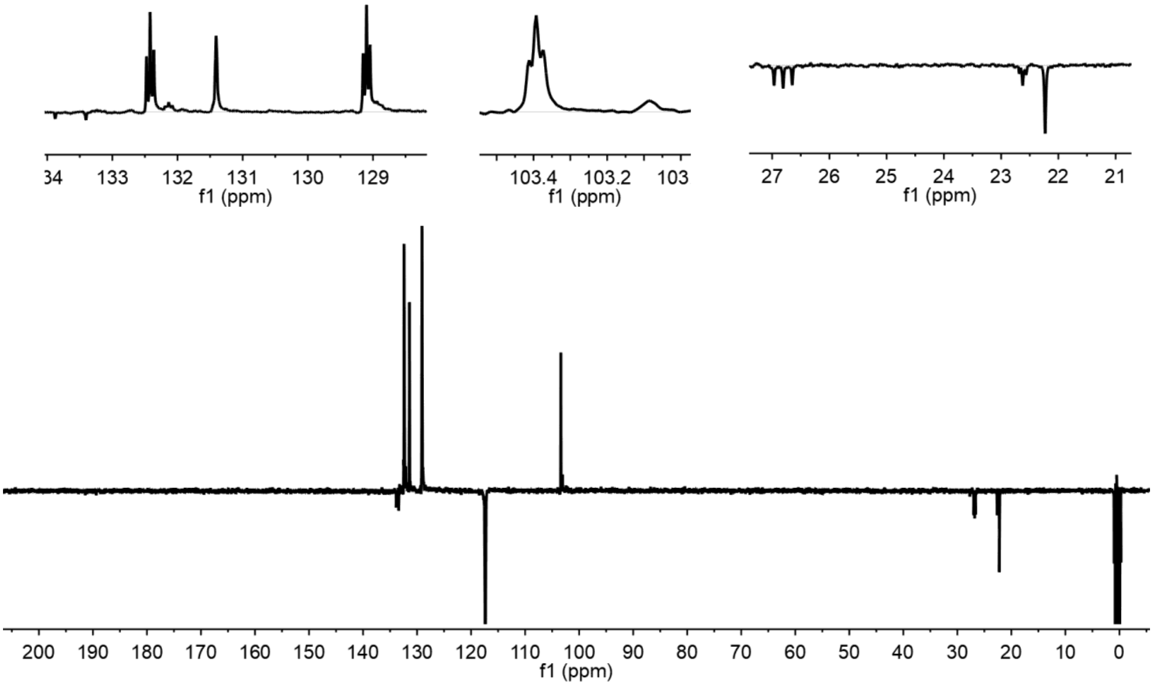

Figure S.41 – J-modulated $^{13}C\{^{1}H\}$ NMR (101 MHz, CD_3CN, 25°C) spectrum of **9**

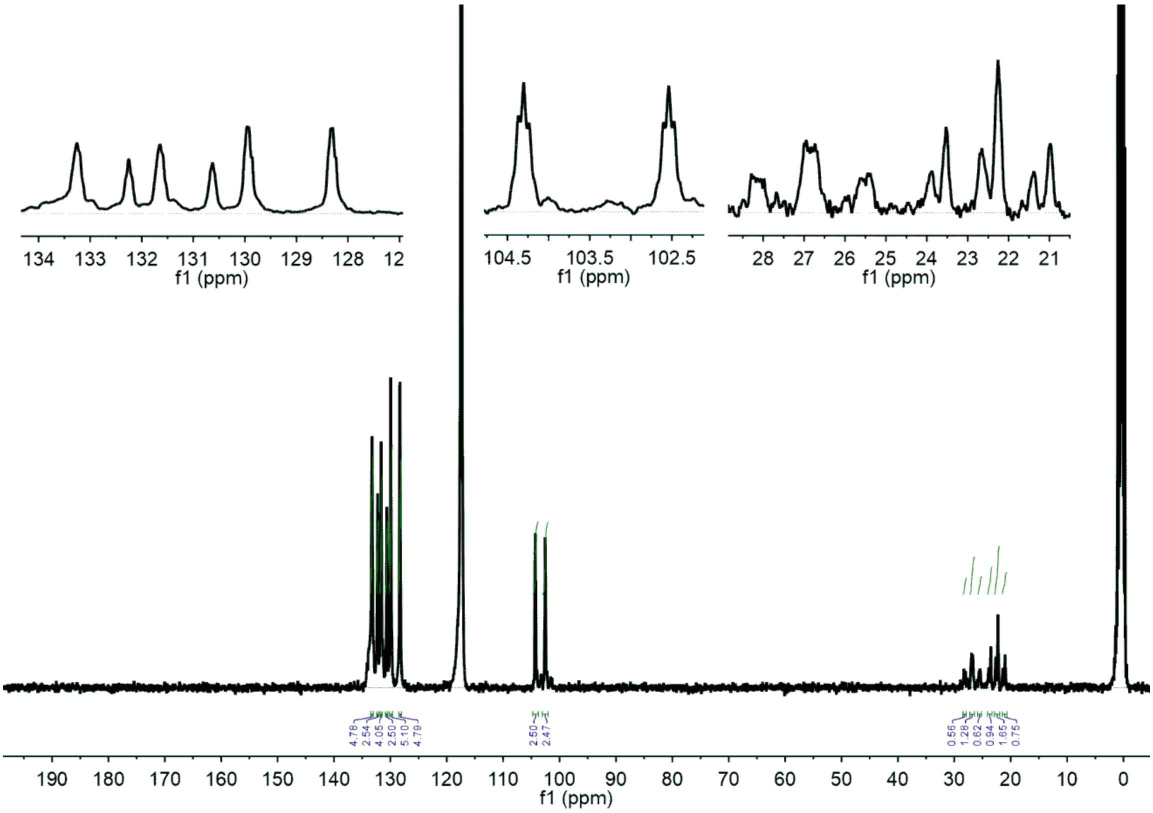

Figure S.42 – $^{13}C\{^{1}H$, gated decoupling mode$\}$ NMR (101 MHz, CD_3CN, 25°C) spectrum of **9**

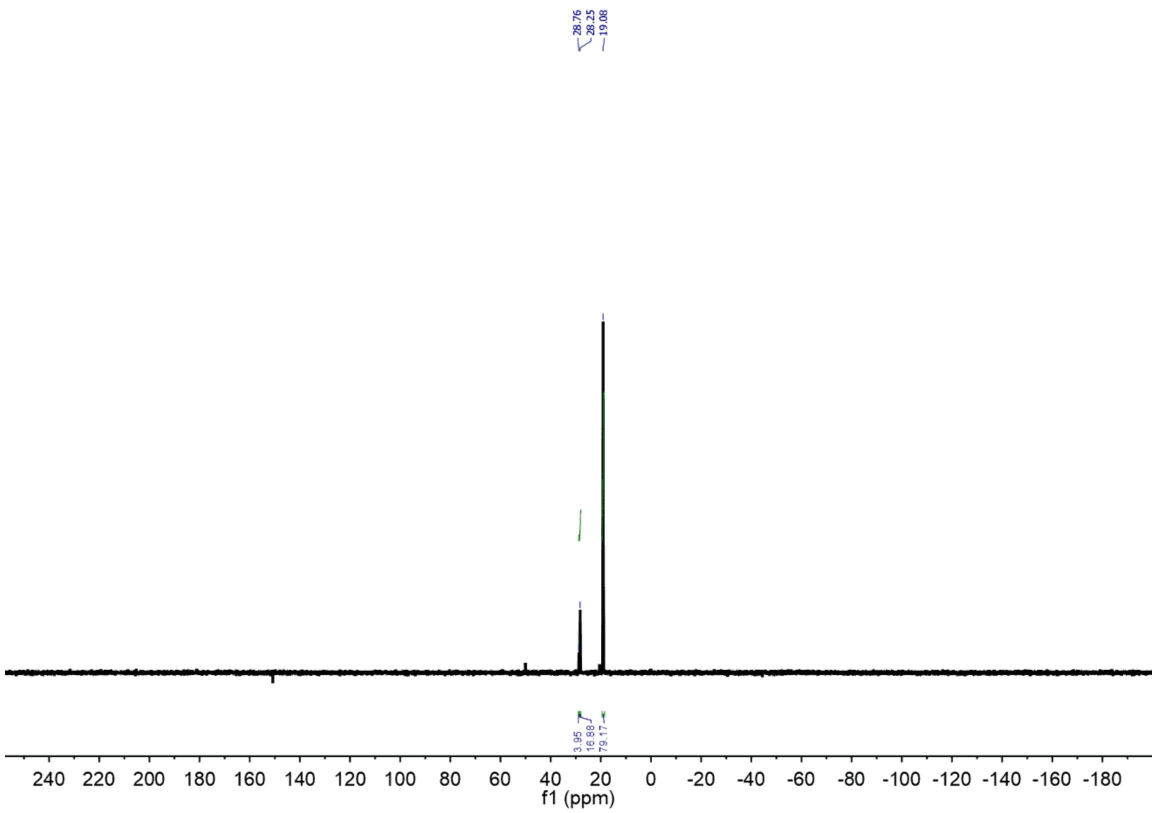

Figure S.43 – $^{31}P\{^{1}H\}$ NMR (162 MHz, CD_3CN, 25°C) spectrum of **9**

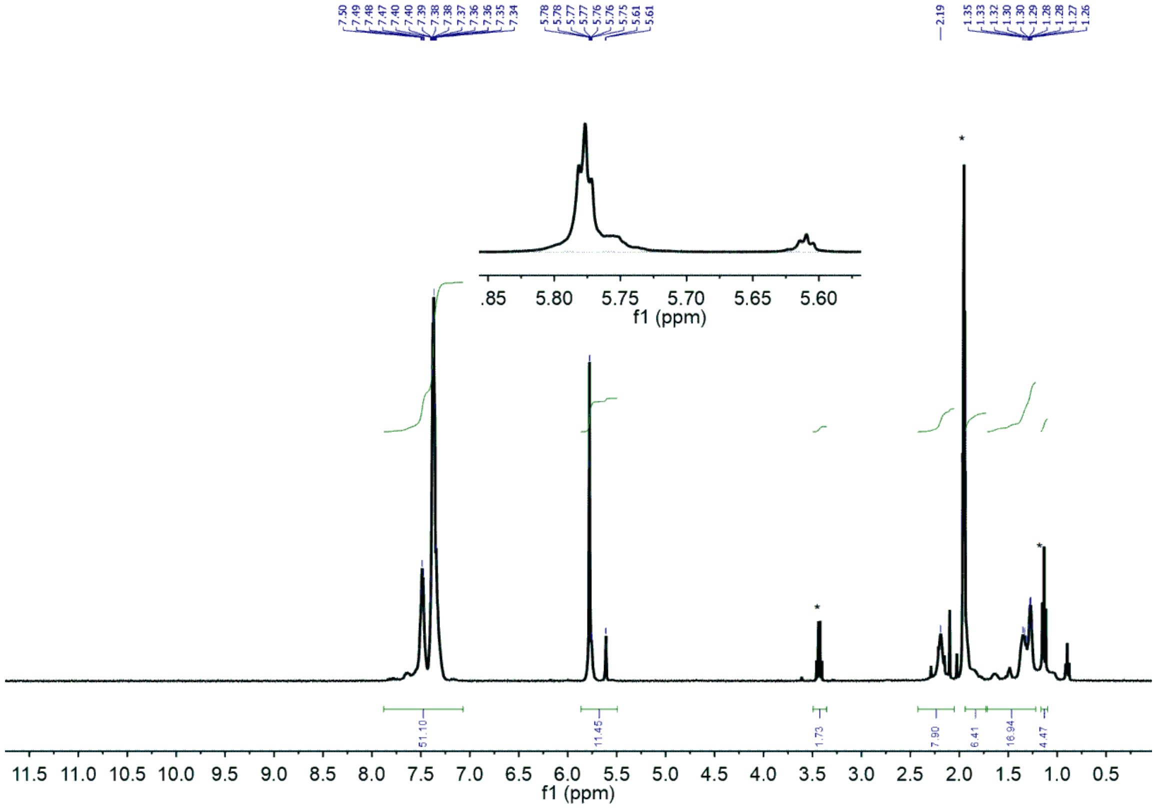

Figure S.44 – ^{1}H NMR (400 MHz, CD_3CN, 25°C) spectrum of **10**

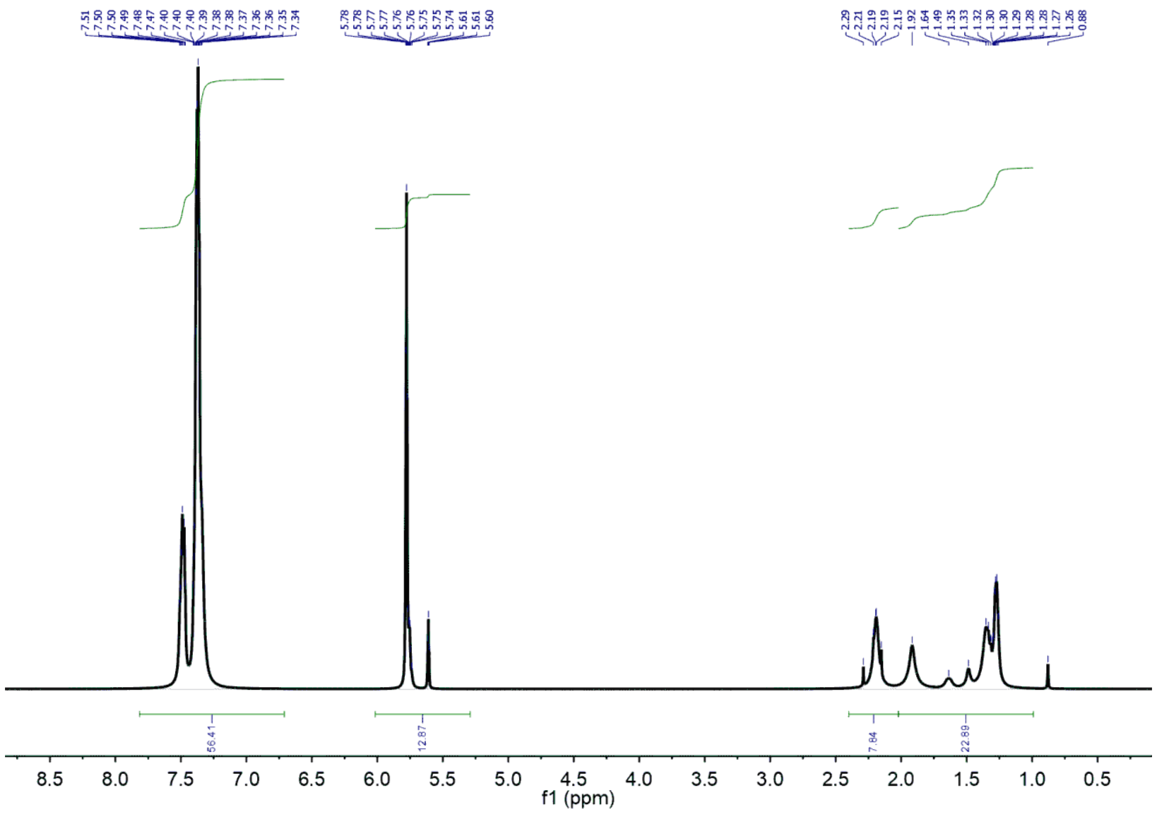

Figure S.45 – ^{1}H (deconvoluted) NMR (400 MHz, CD_3CN, 25°C) spectrum of **10**

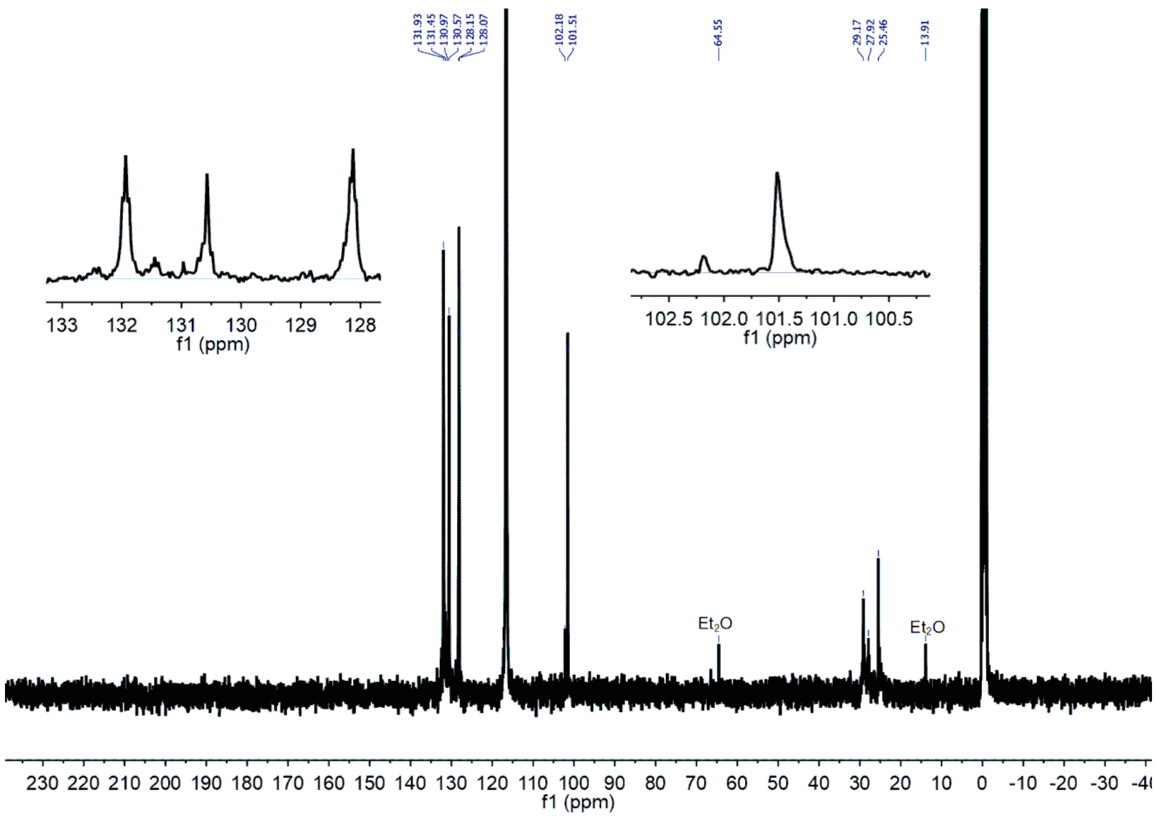

Figure S.46 – ^{13}C{^{1}H} NMR (101 MHz, CD_3CN, 25°C) spectrum of **10**

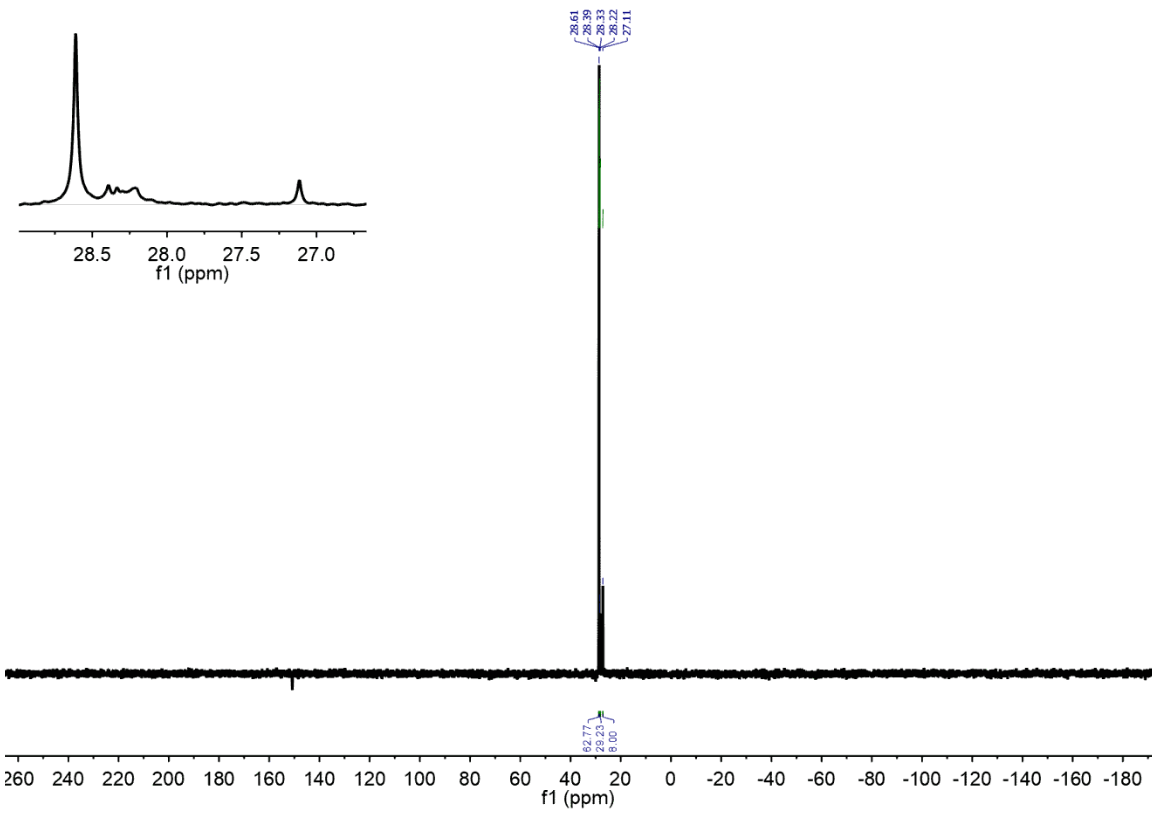

Figure S.47 – $^{31}P\{^1H\}$ NMR (162 MHz, CD_3CN, 25°C) spectrum of **10**

S.4 FTIR spectra

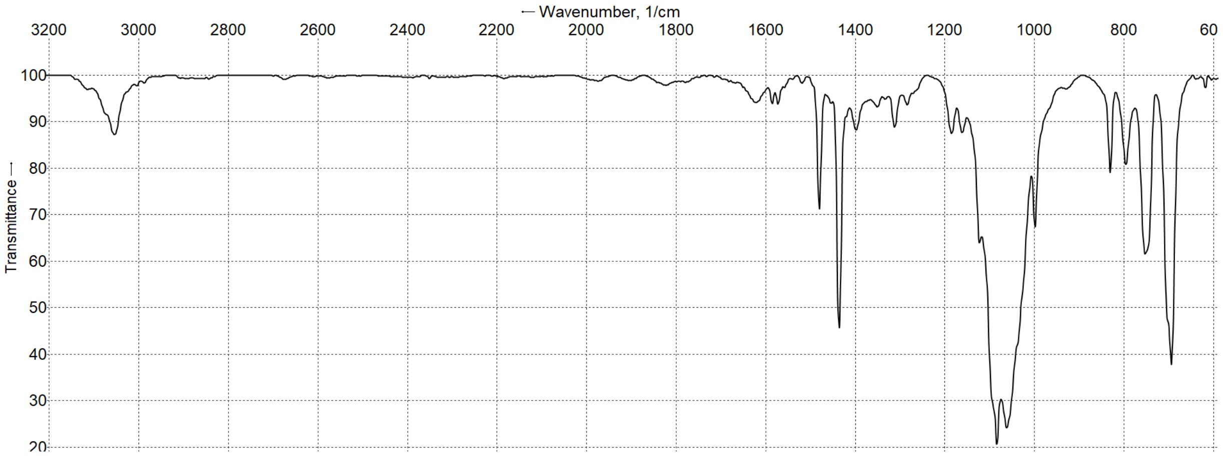

Figure S.48 – FTIR spectrum of **1** (KBr)

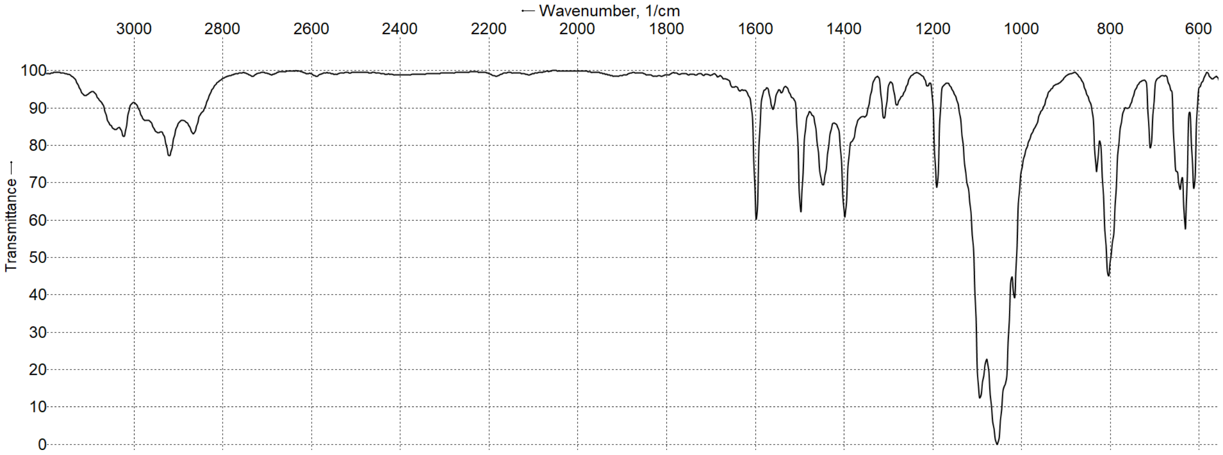

Figure S.49 – FTIR spectrum of **2** (KBr)

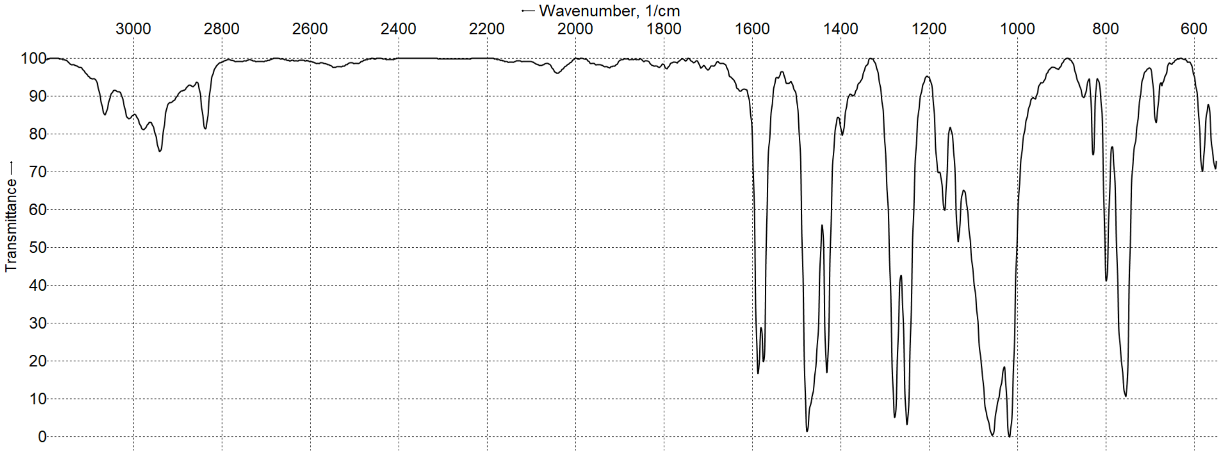

Figure S.50 – FTIR spectrum of **3** (KBr)

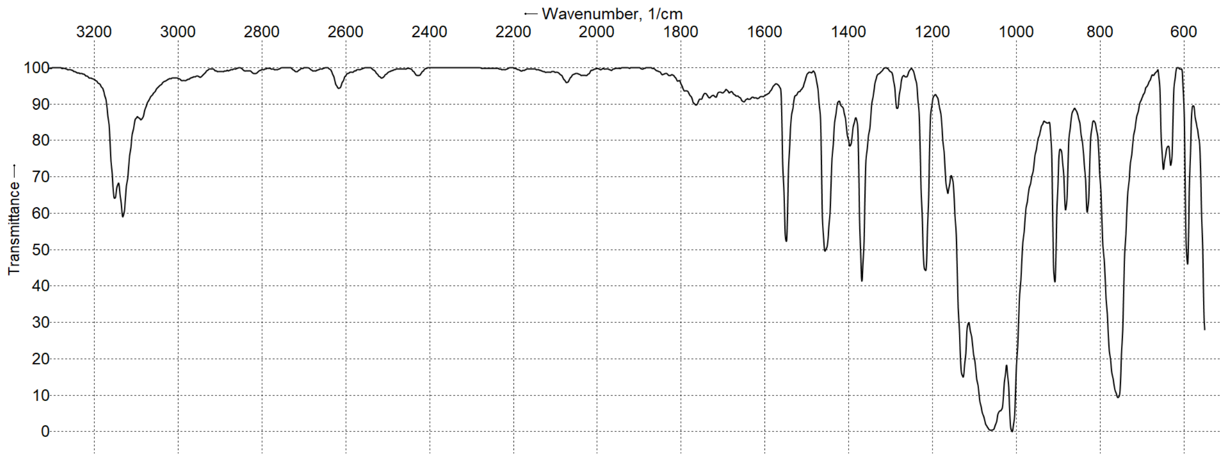

Figure S.51 – FTIR spectrum of **4** (KBr)

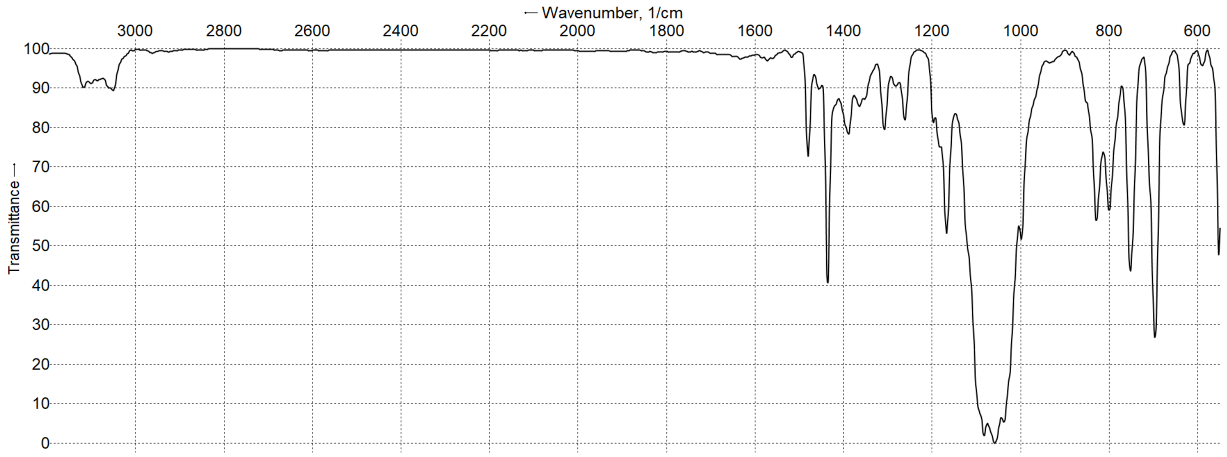

Figure S.52 – FTIR spectrum of **6** (KBr)

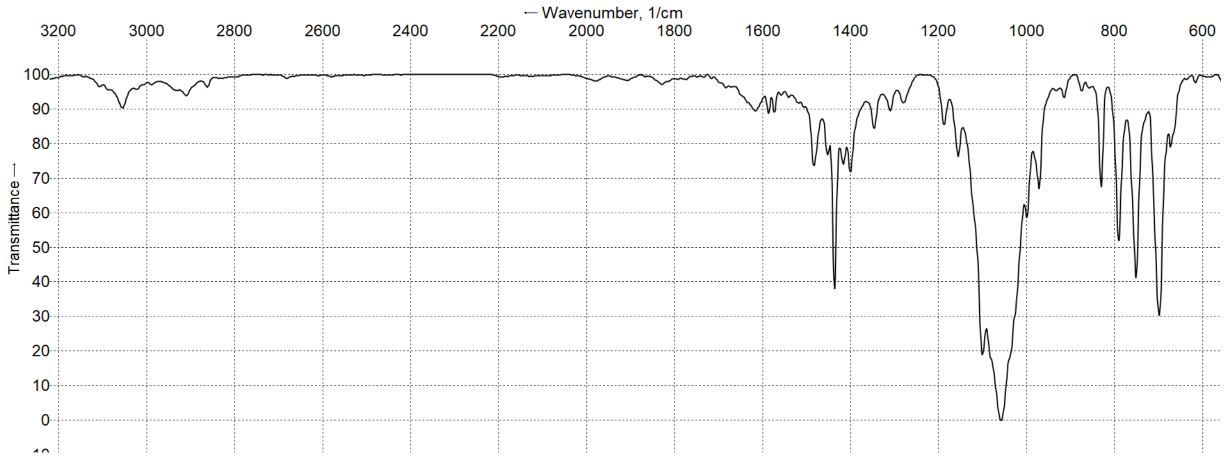

Figure S.53 – FTIR spectrum of **7** (KBr)

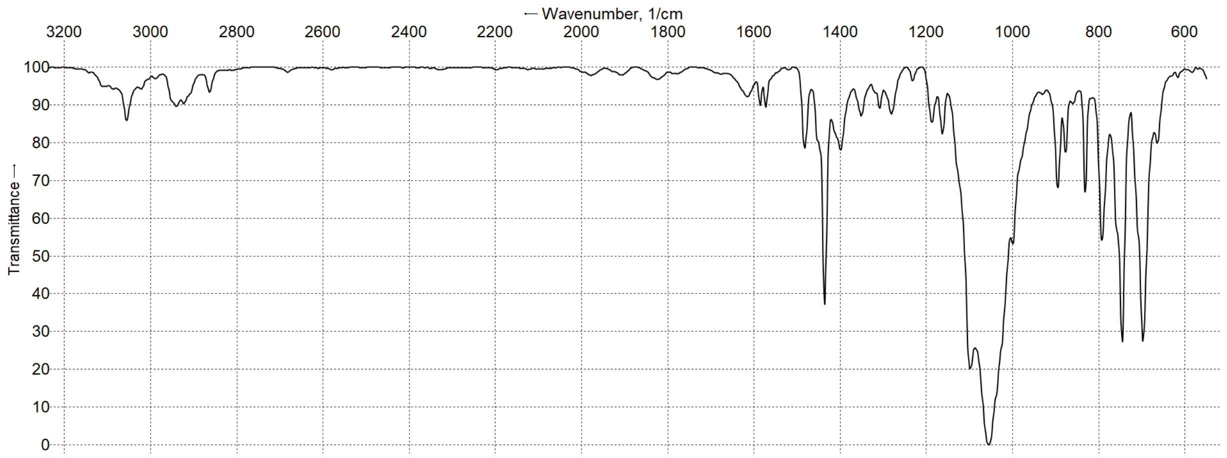

Figure S.54 – FTIR spectrum of **8** (KBr)

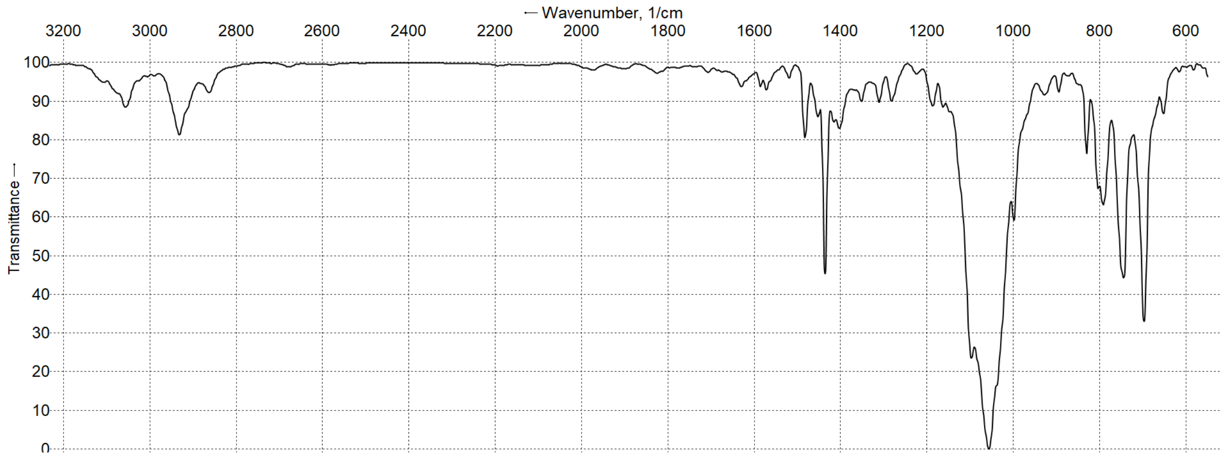

Figure S.55 – FTIR spectrum of **9** (KBr)

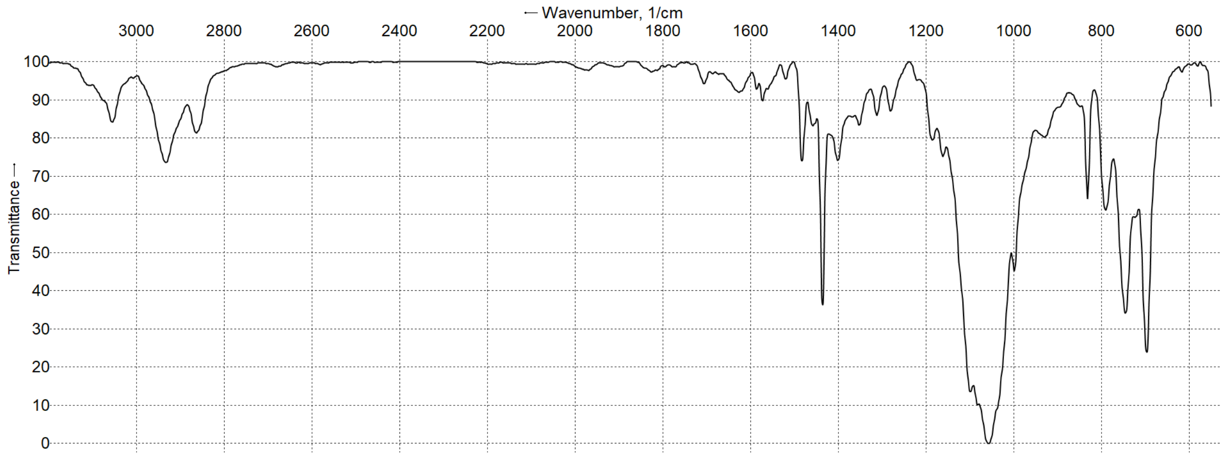

Figure S.56 – FTIR spectrum of **10** (KBr)

S.5 ESI-MS spectra

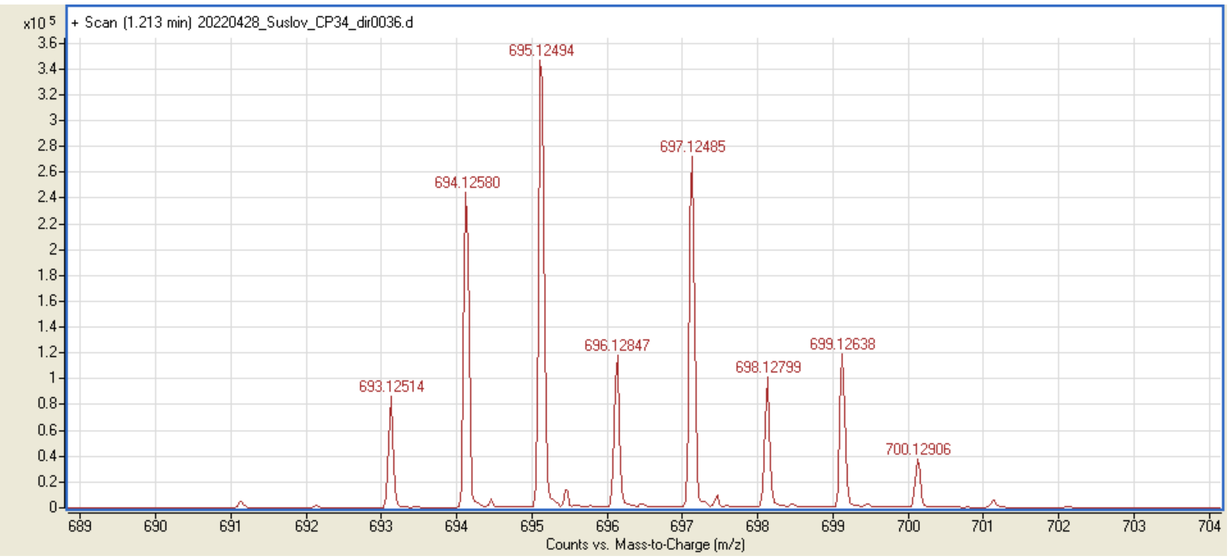

Figure S.57 – MS spectrum of **1** (MeCN, positive ion mode)

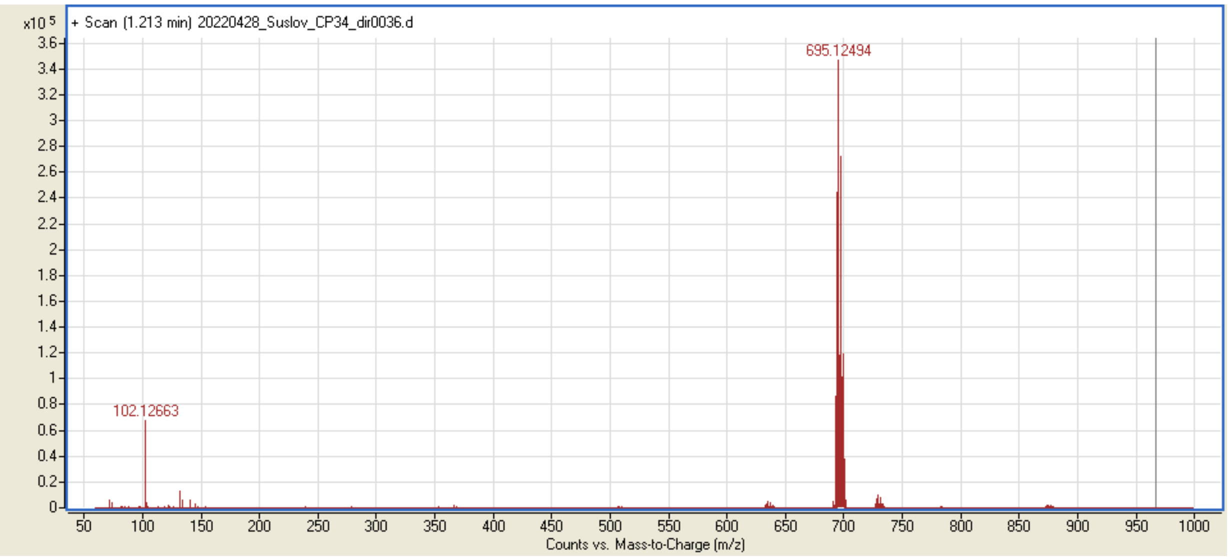

Figure S.58 – MS spectrum of **1** (MeCN, positive ion mode)

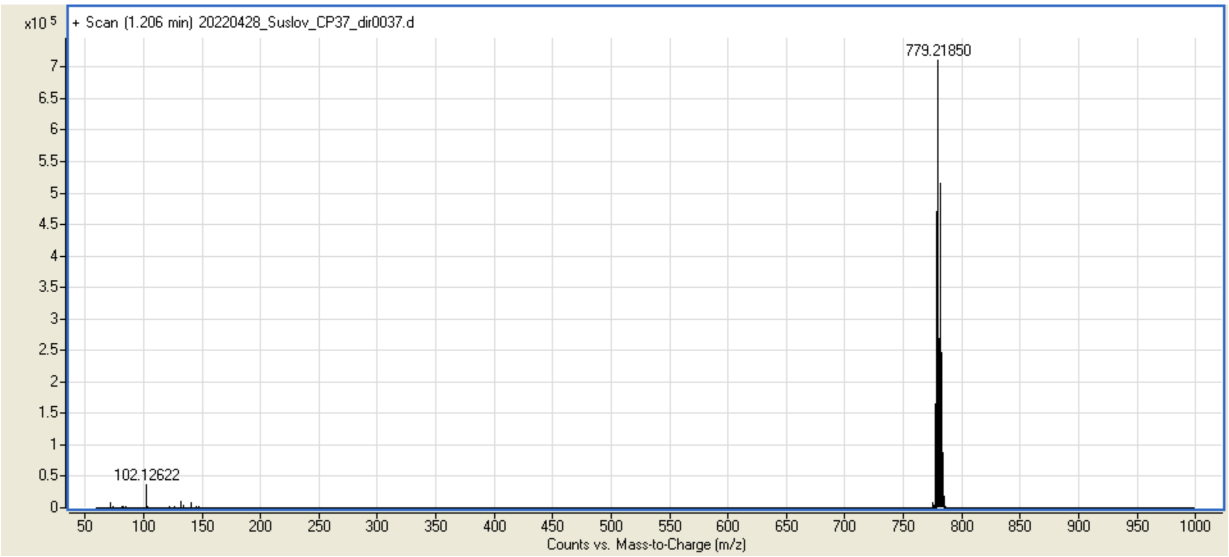

Figure S.59 – MS spectrum of **2** (MeCN, positive ion mode)

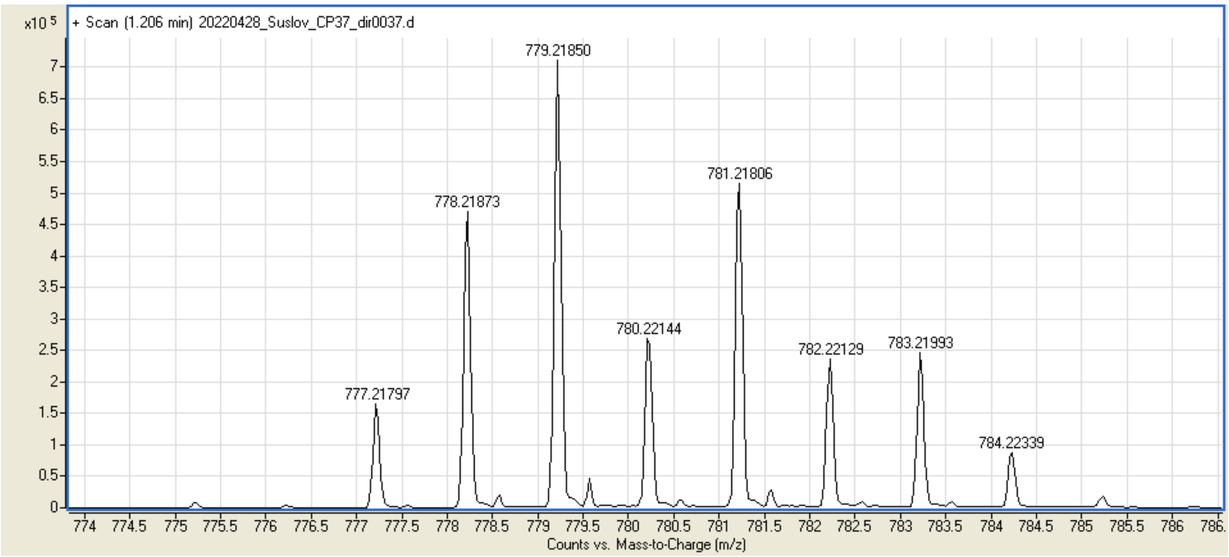

Figure S.60 – MS spectrum of **2** (MeCN, positive ion mode)

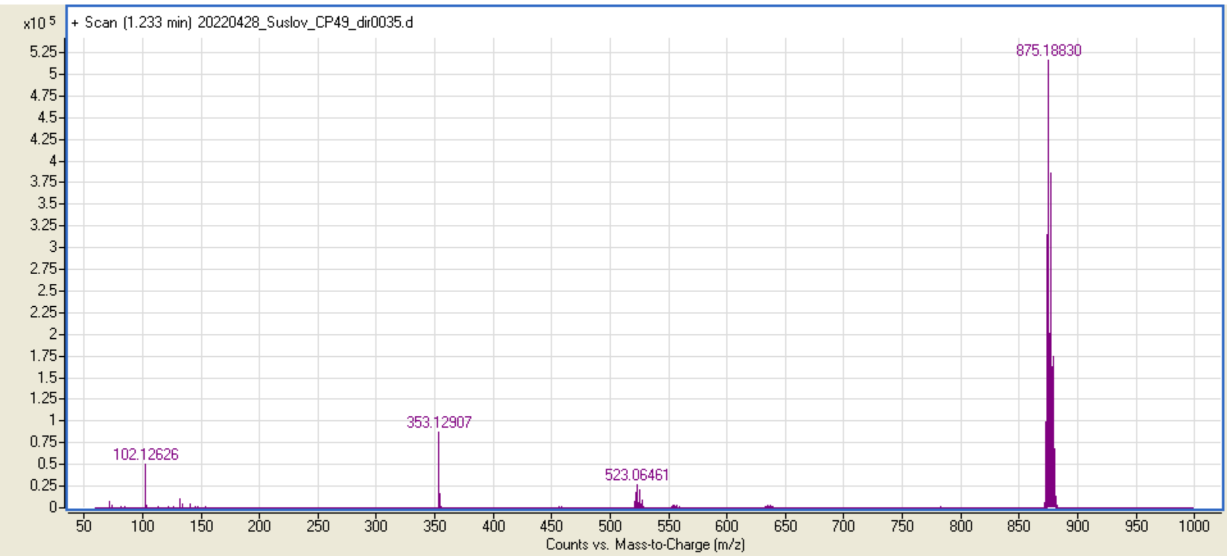

Figure S.61 – MS spectrum of **3** (MeCN, positive ion mode)

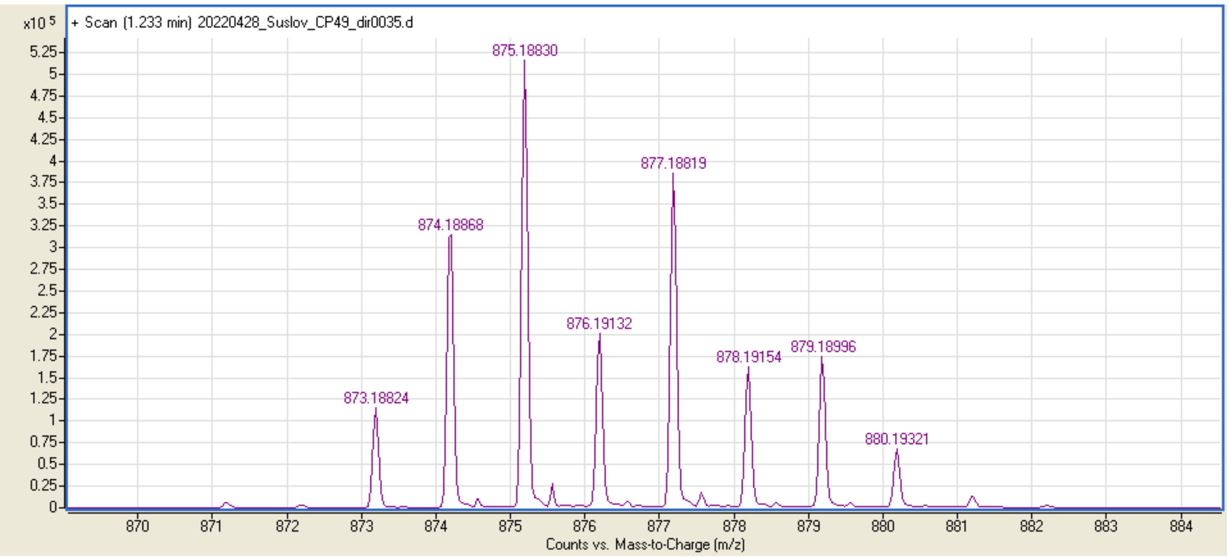

Figure S.62 – MS spectrum of **3** (MeCN, positive ion mode)

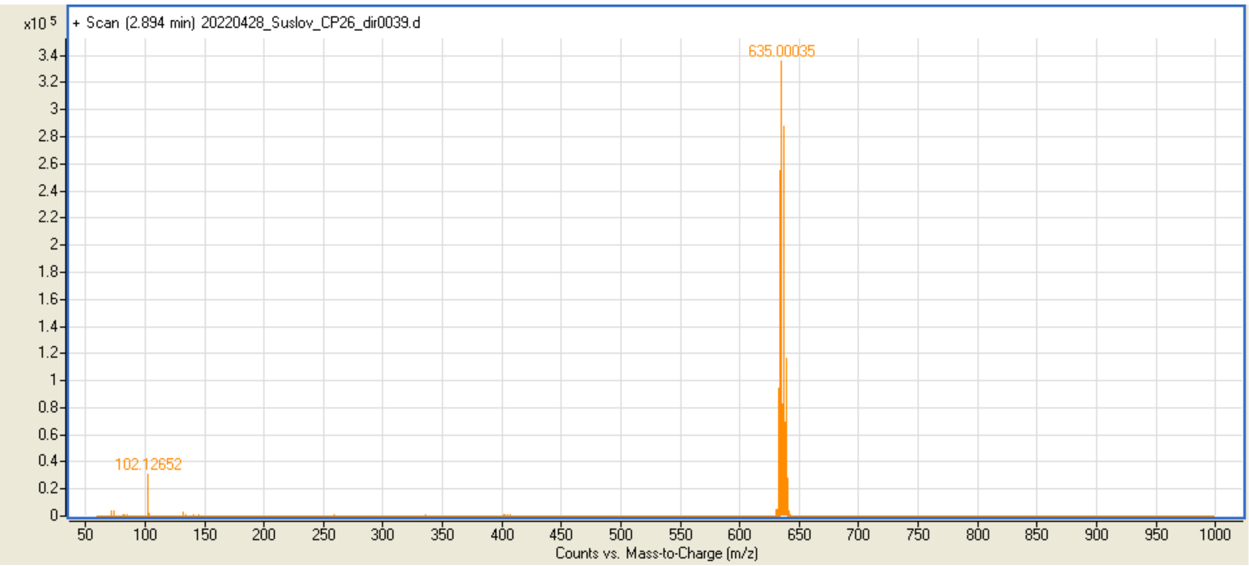

Figure S.63 – MS spectrum of **4** (MeCN, positive ion mode)

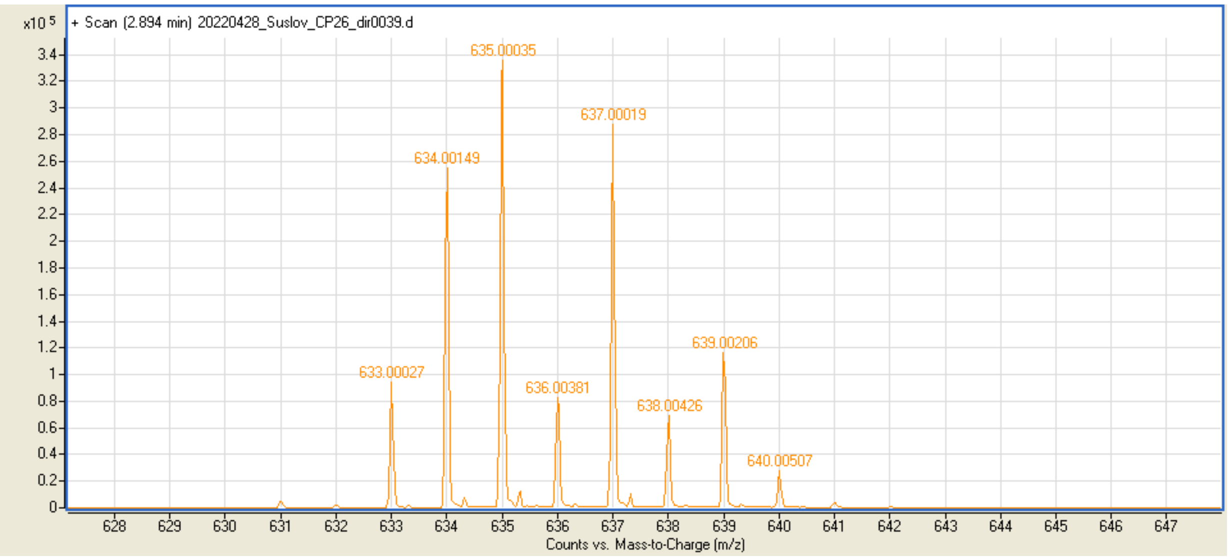

Figure S.64 – MS spectrum of **4** (MeCN, positive ion mode)

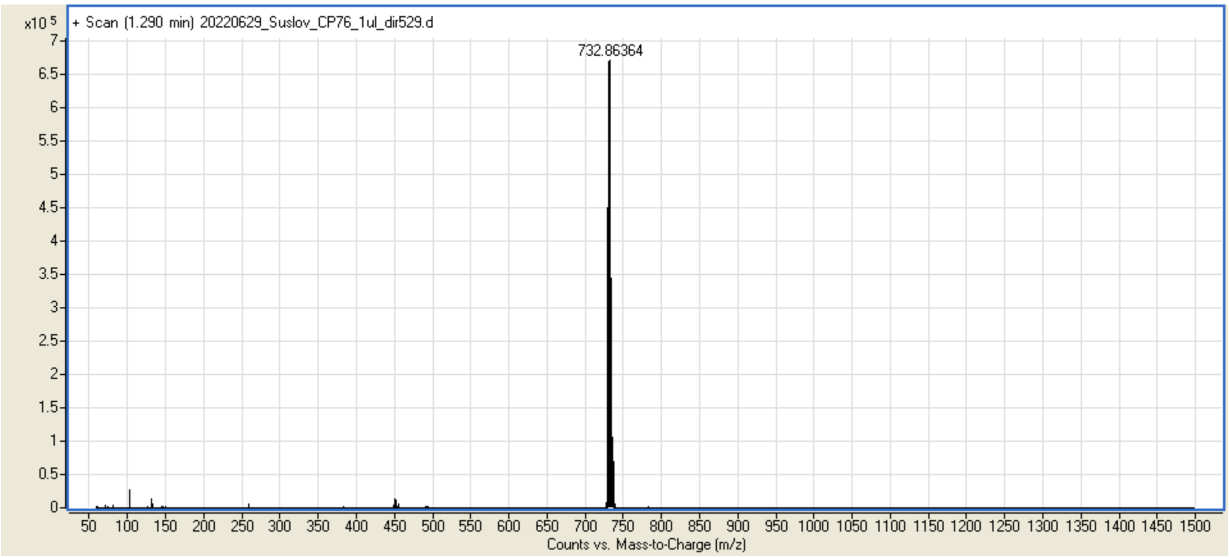

Figure S.65 – MS spectrum of **5** (MeCN, positive ion mode)

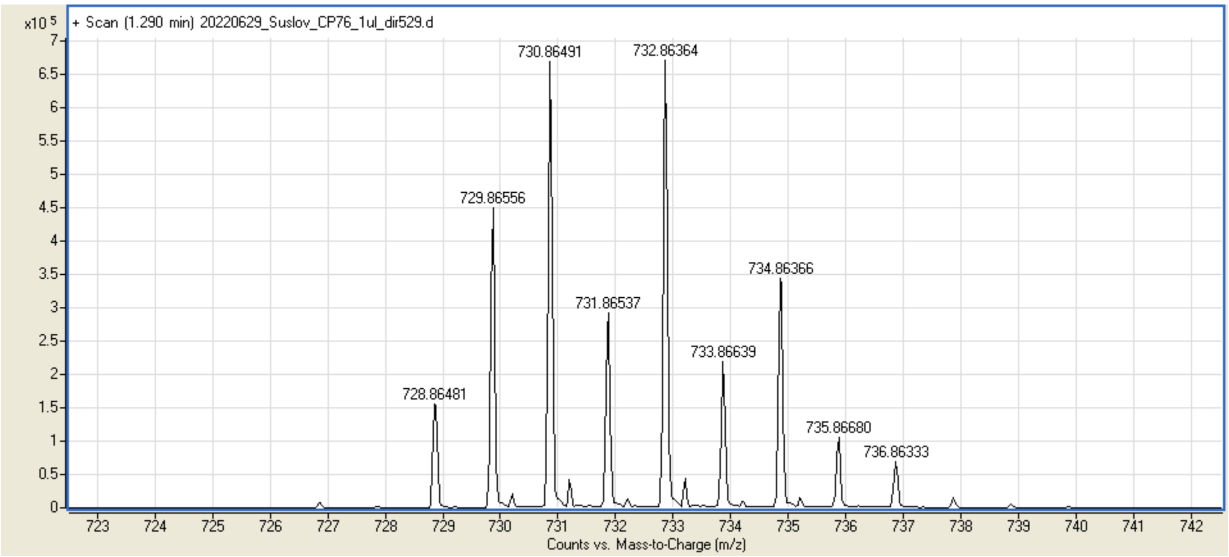

Figure S.66 – MS spectrum of **5** (MeCN, positive ion mode)

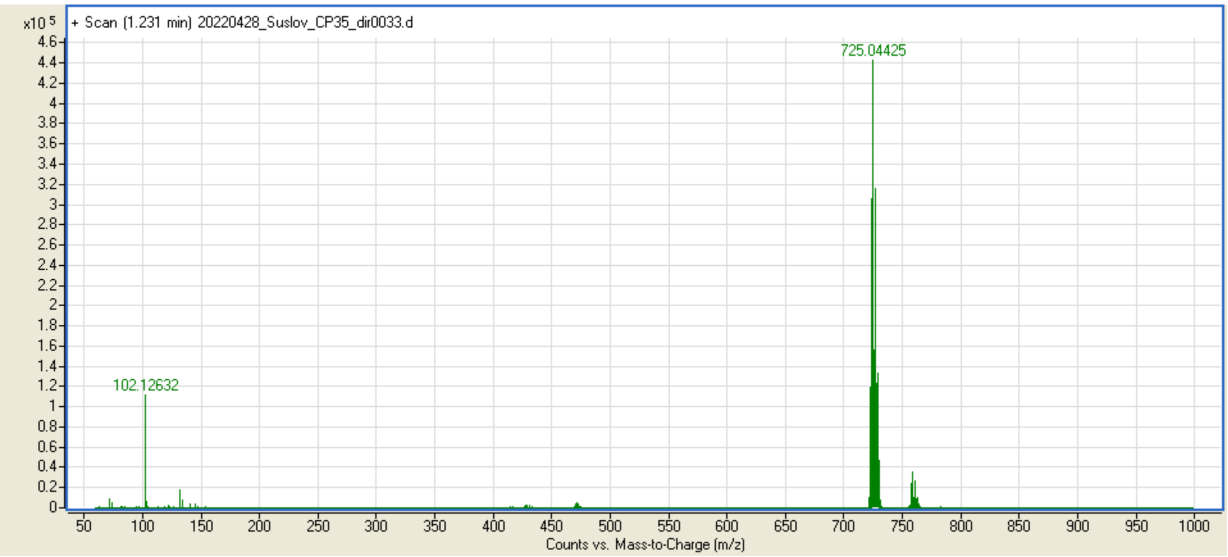

Figure S.67 – MS spectrum of **6** (MeCN, positive ion mode)

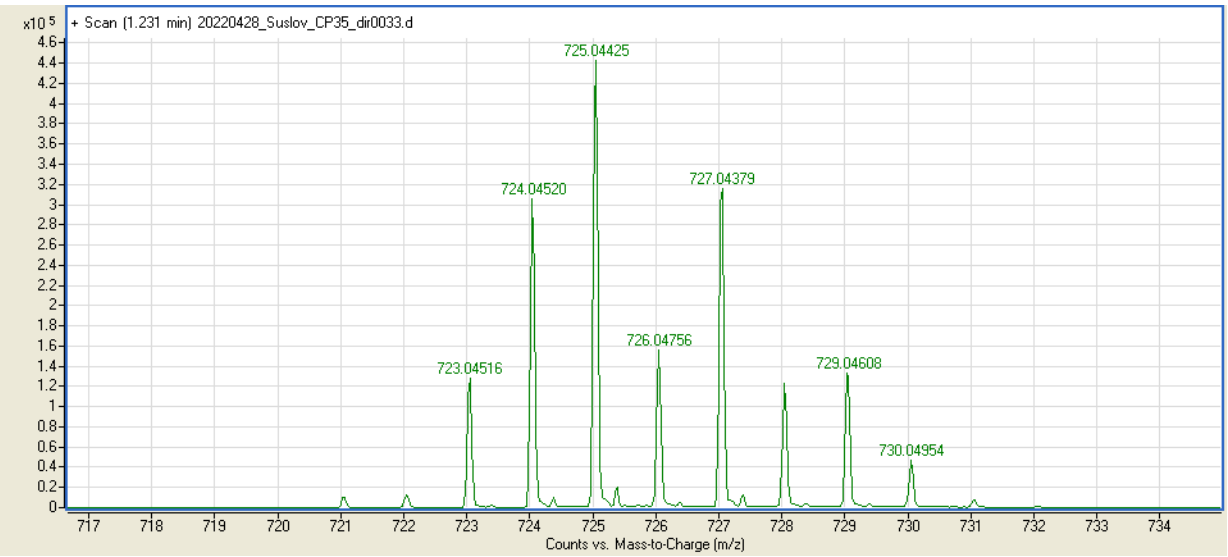

Figure S.68 – MS spectrum of **6** (MeCN, positive ion mode)

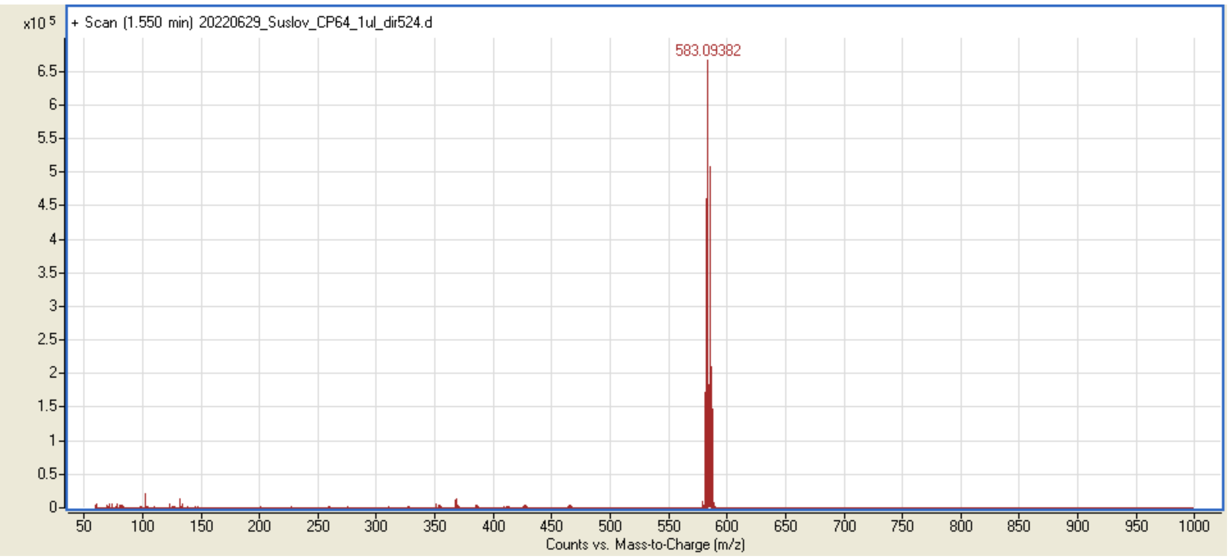

Figure S.69 – MS spectrum of **7** (MeCN, positive ion mode)

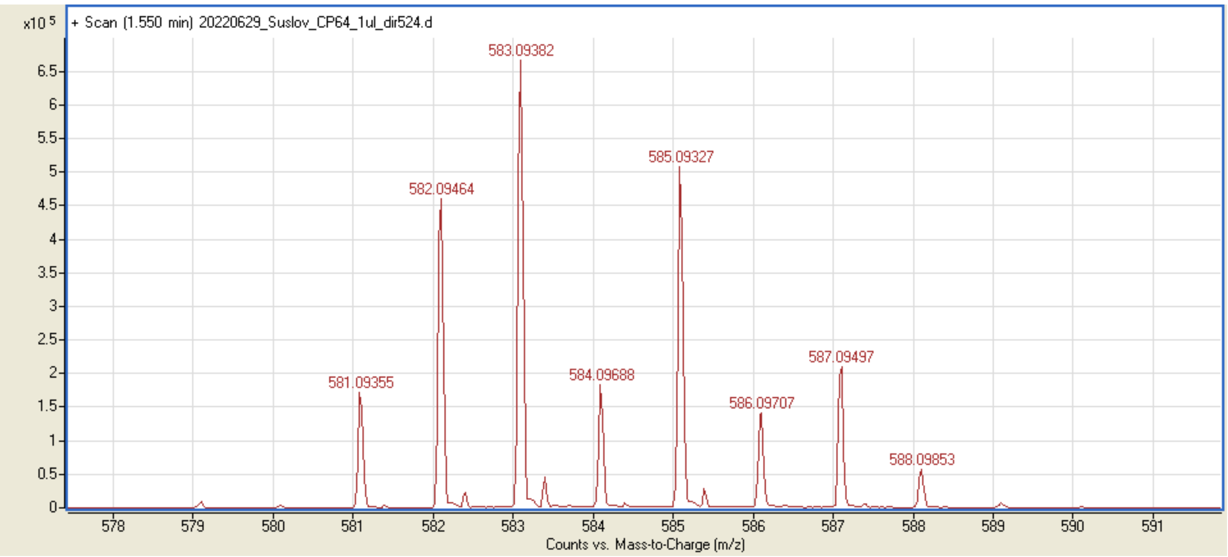

Figure S.70 – MS spectrum of **7** (MeCN, positive ion mode)

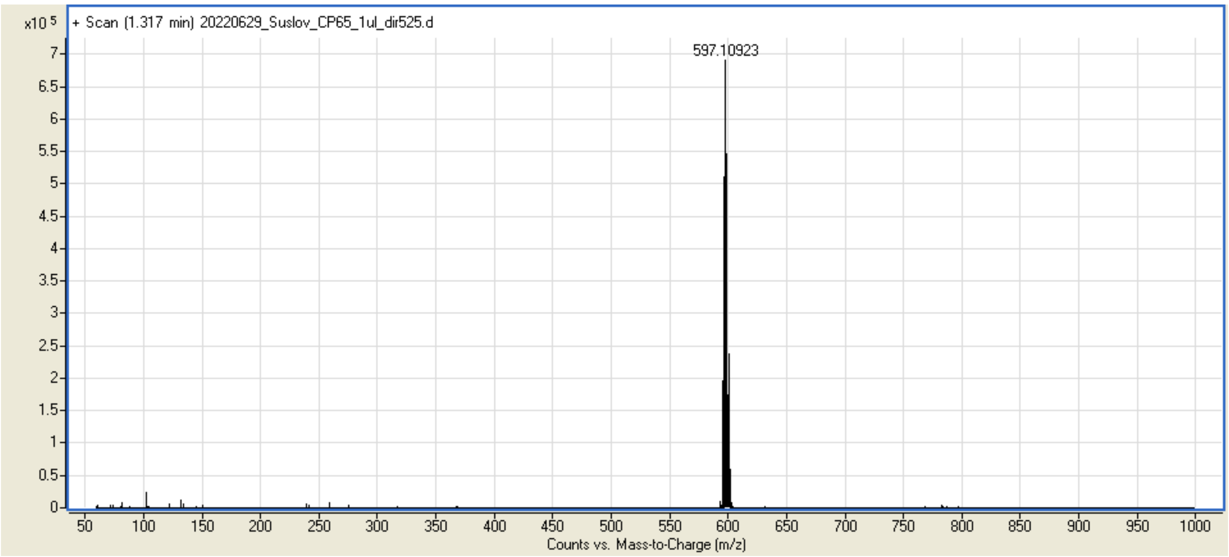

Figure S.71 – MS spectrum of **8** (MeCN, positive ion mode)

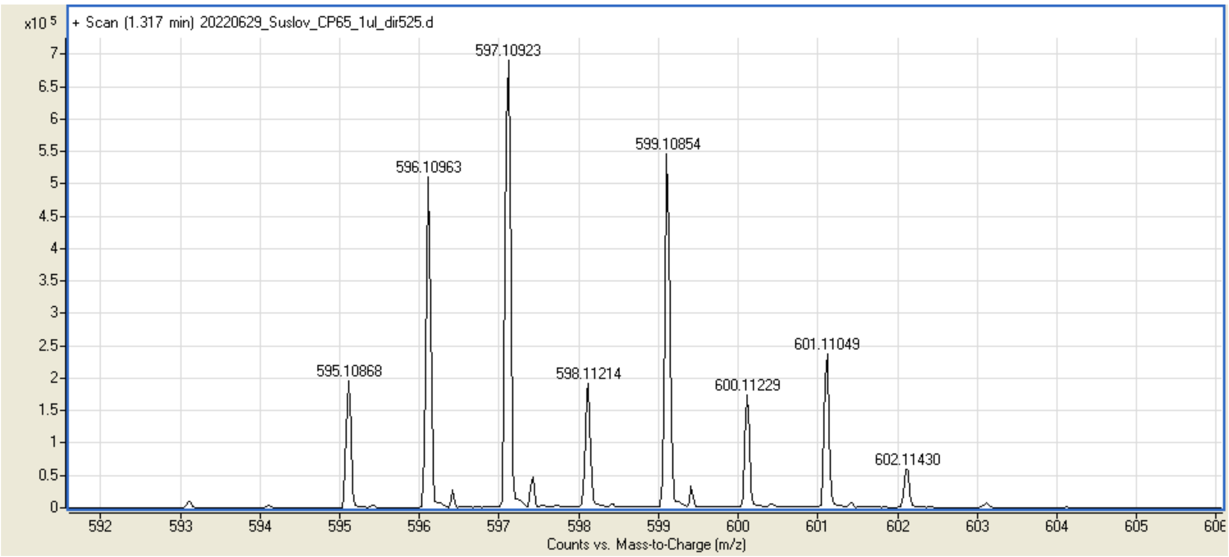

Figure S.72 – MS spectrum of **8** (MeCN, positive ion mode)

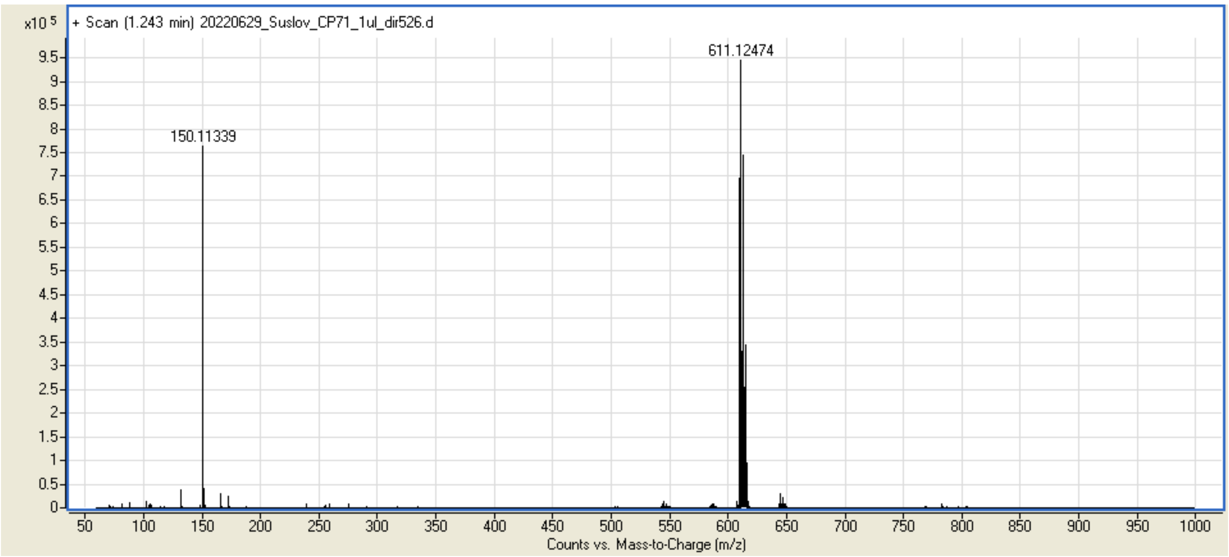

Figure S.73 – MS spectrum of **9** (MeCN, positive ion mode)

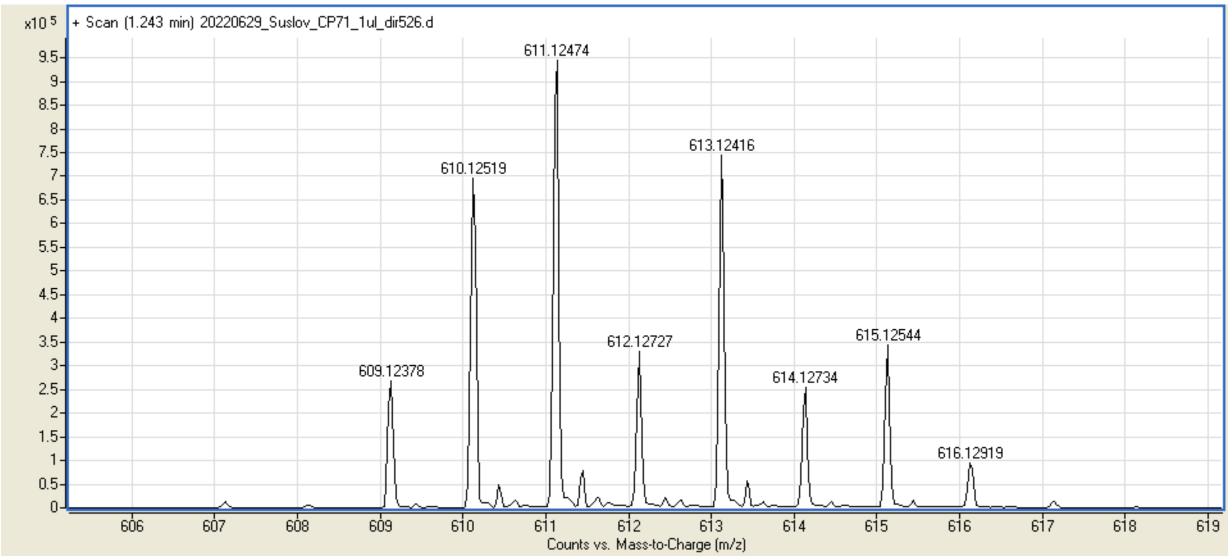

Figure S.74 – MS spectrum of **9** (MeCN, positive ion mode)

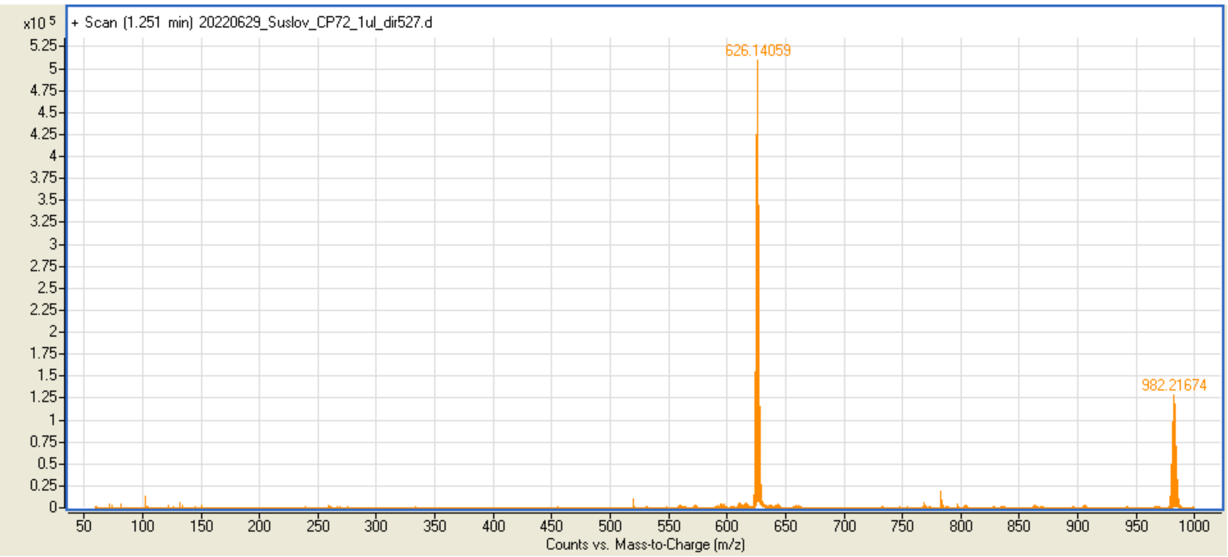

Figure S.75 – MS spectrum of **10** (MeCN, positive ion mode)

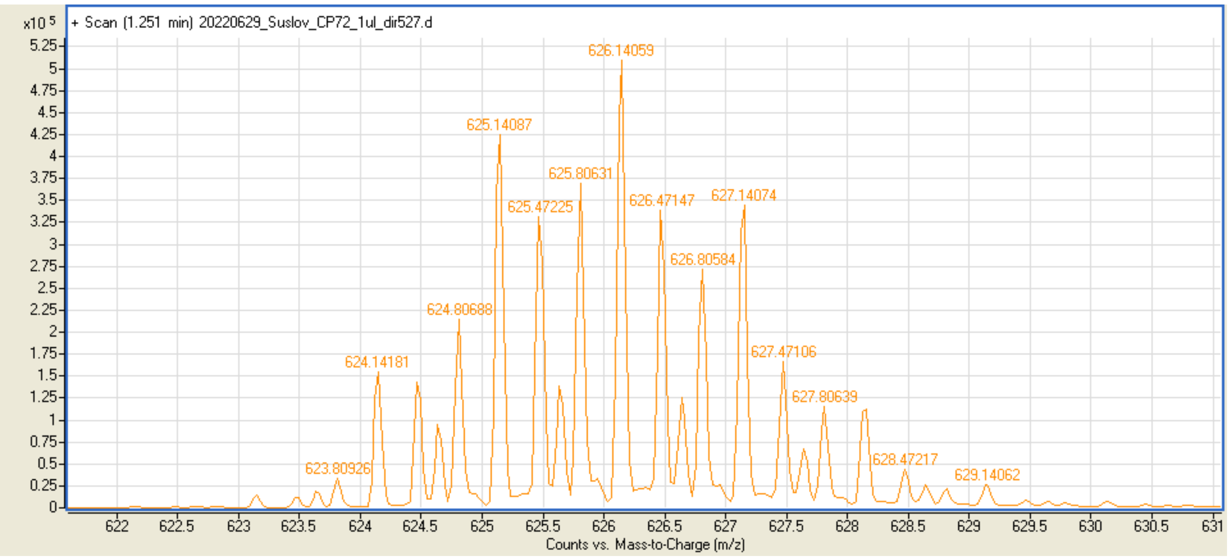

Figure S.76 – MS spectrum of **10** (MeCN, positive ion mode)

S.6 UV-vis spectra

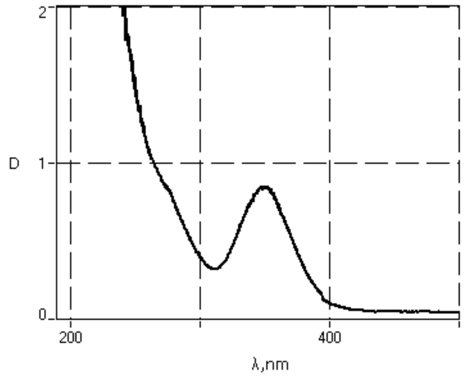

Figure S.77 – UV-vis spectrum of **1** (*C* = 0.5 mM, solvent – 1,2-$C_2H_4Cl_2$)

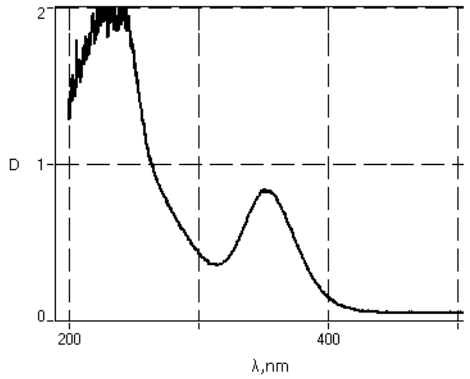

Figure S.78 – UV-vis spectrum of **2** (*C* = 0.5 mM, solvent – 1,2-$C_2H_4Cl_2$)

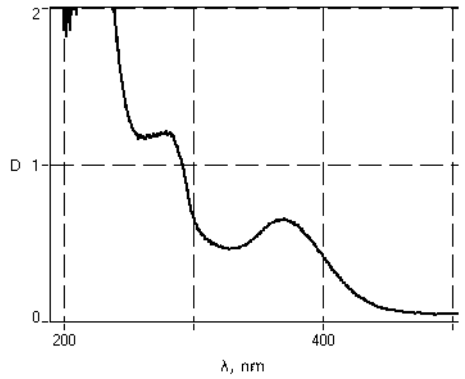

Figure S.79 – UV-vis spectrum of **3** (*C* = 0.5 mM, solvent – 1,2-$C_2H_4Cl_2$)

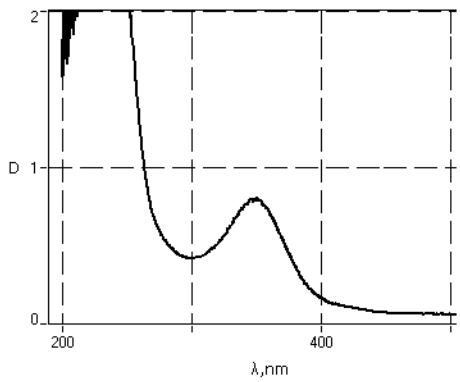

Figure S.80 – UV-vis spectrum of **4** (C = 0.5 mM, solvent – 1,2-$C_2H_4Cl_2$)

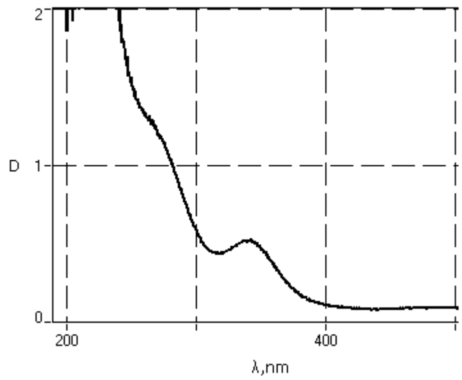

Figure S.81 – UV-vis spectrum of **6** (C = 0.5 mM, solvent – 1,2-$C_2H_4Cl_2$)

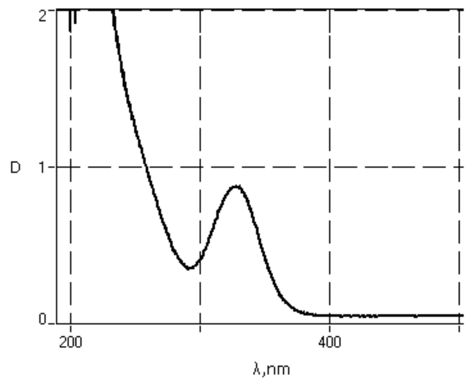

Figure S.82 – UV-vis spectrum of **7** (C = 0.5 mM, solvent – 1,2-$C_2H_4Cl_2$)

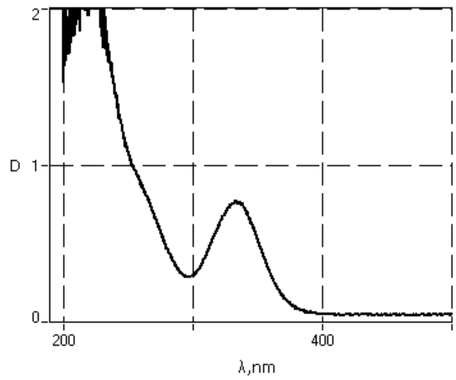

Figure S.83 – UV-vis spectrum of **8** (*C* = 0.5 mM, solvent – 1,2-$C_2H_4Cl_2$)

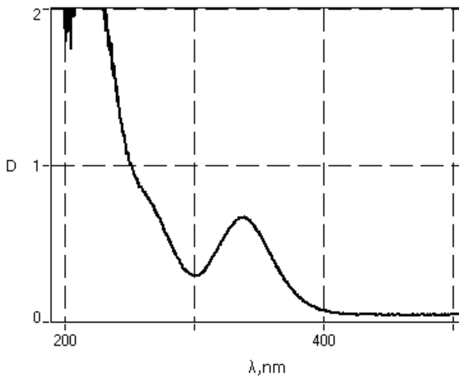

Figure S.84 – UV-vis spectrum of **9** (*C* = 0.5 mM, solvent – 1,2-$C_2H_4Cl_2$)

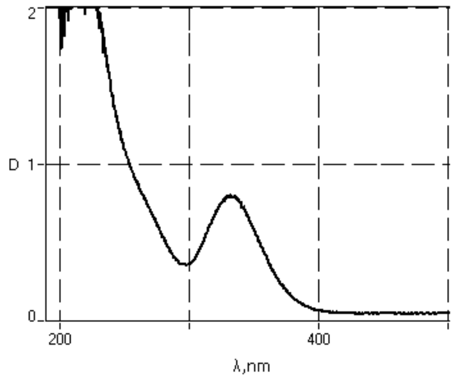

Figure S.85 – UV-vis spectrum of **10** (*C* = 0.5 mM, solvent – 1,2-$C_2H_4Cl_2$)

S.7 Reaction of [Pd(κ^2-O,O′-acac)(TOMPP)$_2$]BF_4 with BF_3·OEt_2 in the presence of MeCN

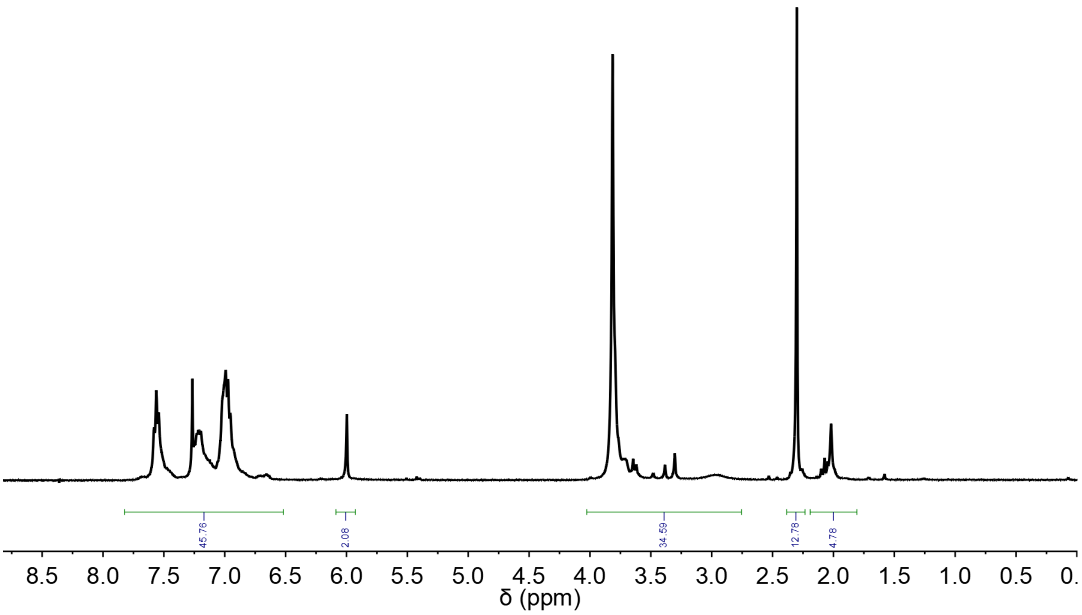

Figure S.86 – ^{1}H NMR (400 MHz, $CDCl_3$, 25°C) spectrum

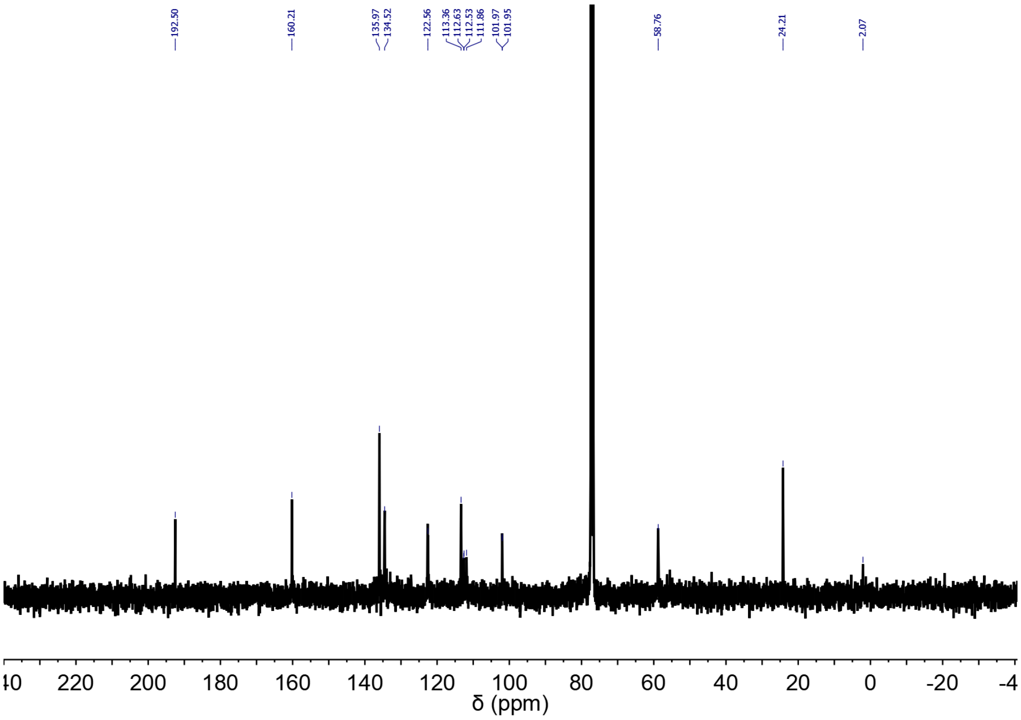

Figure S.87 – ^{13}C{^{1}H} NMR (101 MHz, $CDCl_3$, 25°C) spectrum

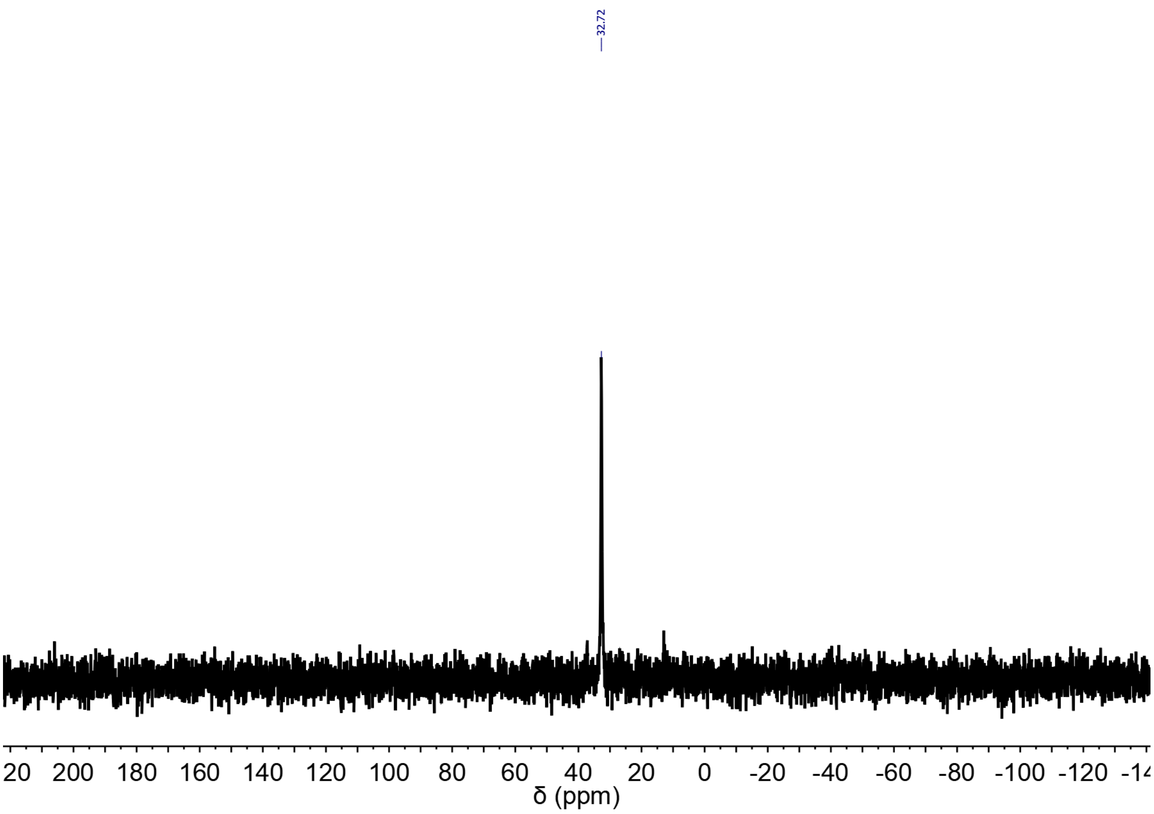

Figure S.88 – $^{31}P\{^{1}H\}$ NMR (162 MHz, $CDCl_3$, 25°C) spectrum

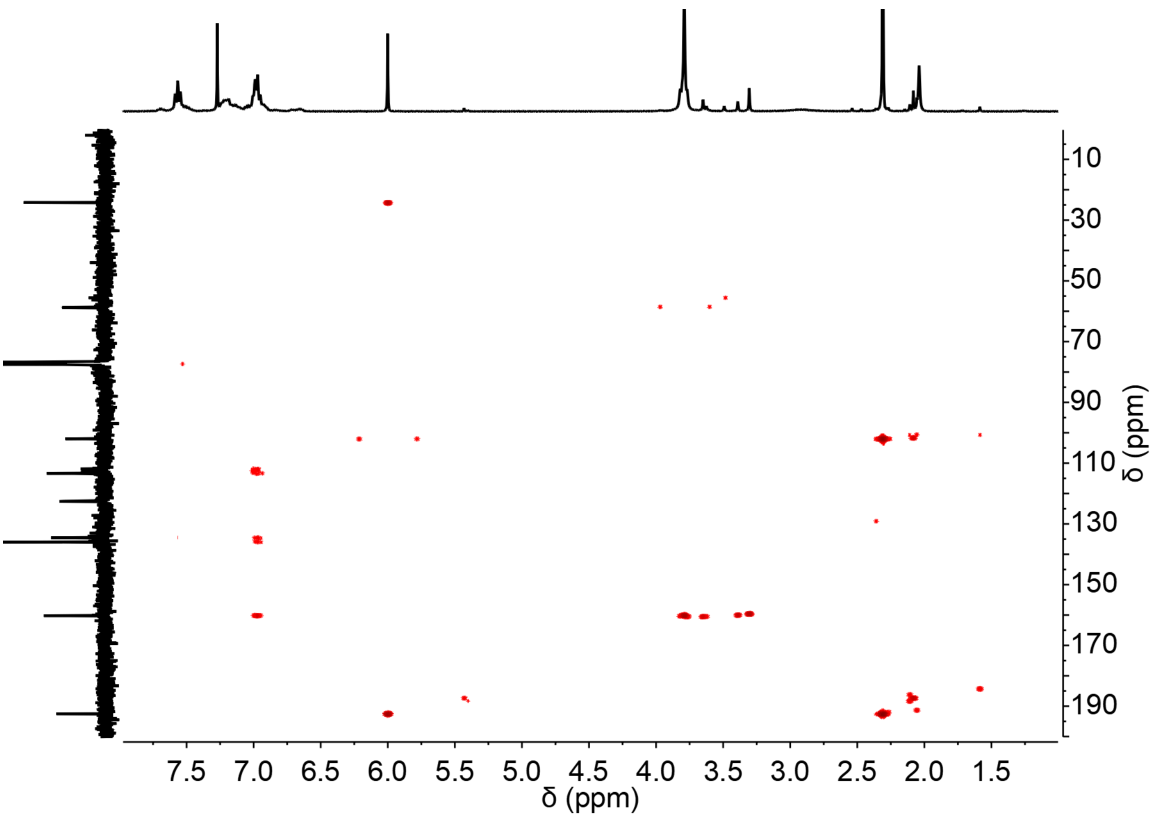

Figure S.89 – ^{1}H–^{13}C HMBC NMR (400 MHz, $CDCl_3$, 25°C) spectrum

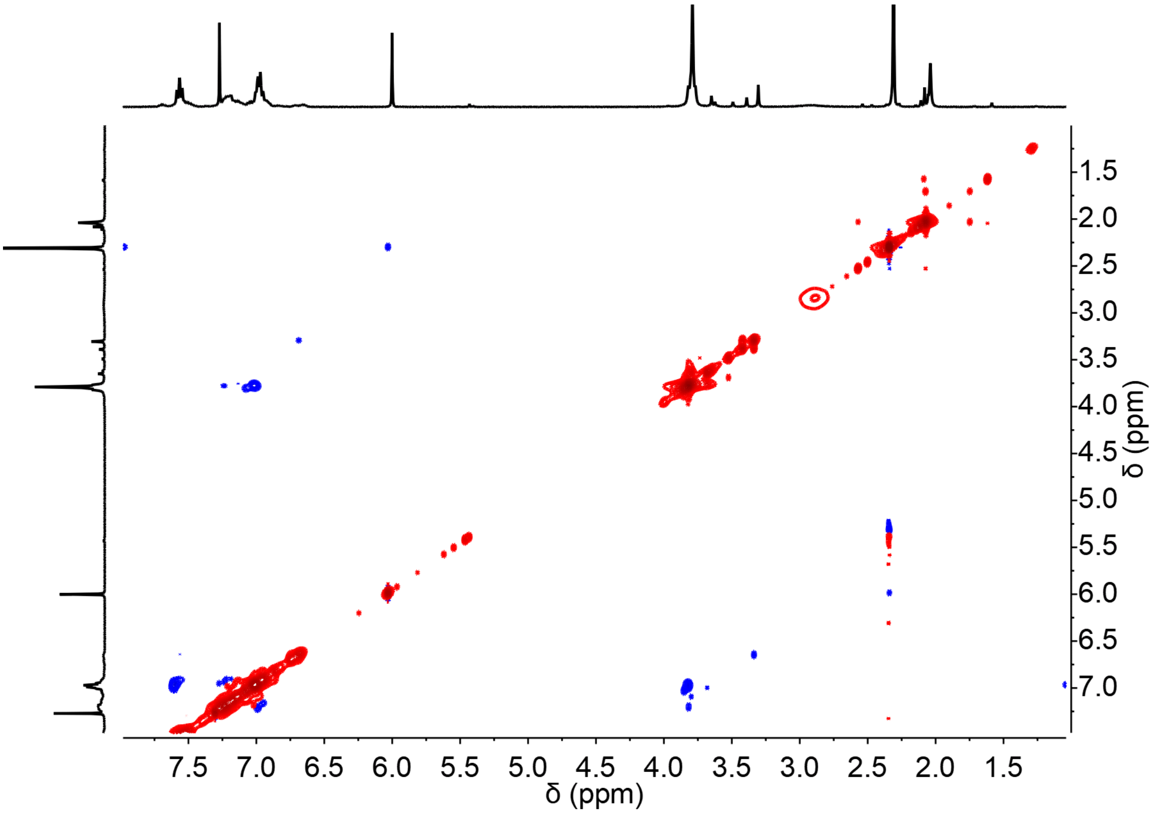

Figure S.90 – ^{1}H–^{1}H NOESY NMR (400 MHz, $CDCl_3$, 25°C) spectrum of **6**

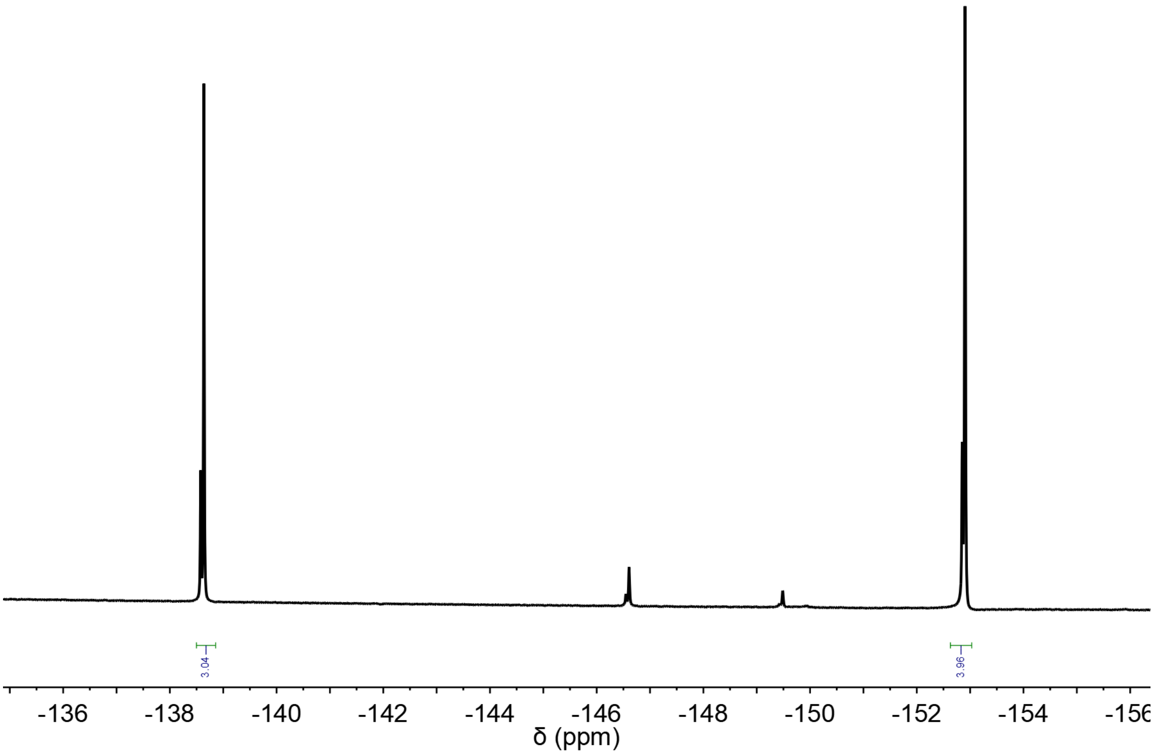

Figure S.91 – ^{19}F NMR (376 MHz, $CDCl_3$, 25°C) spectrum

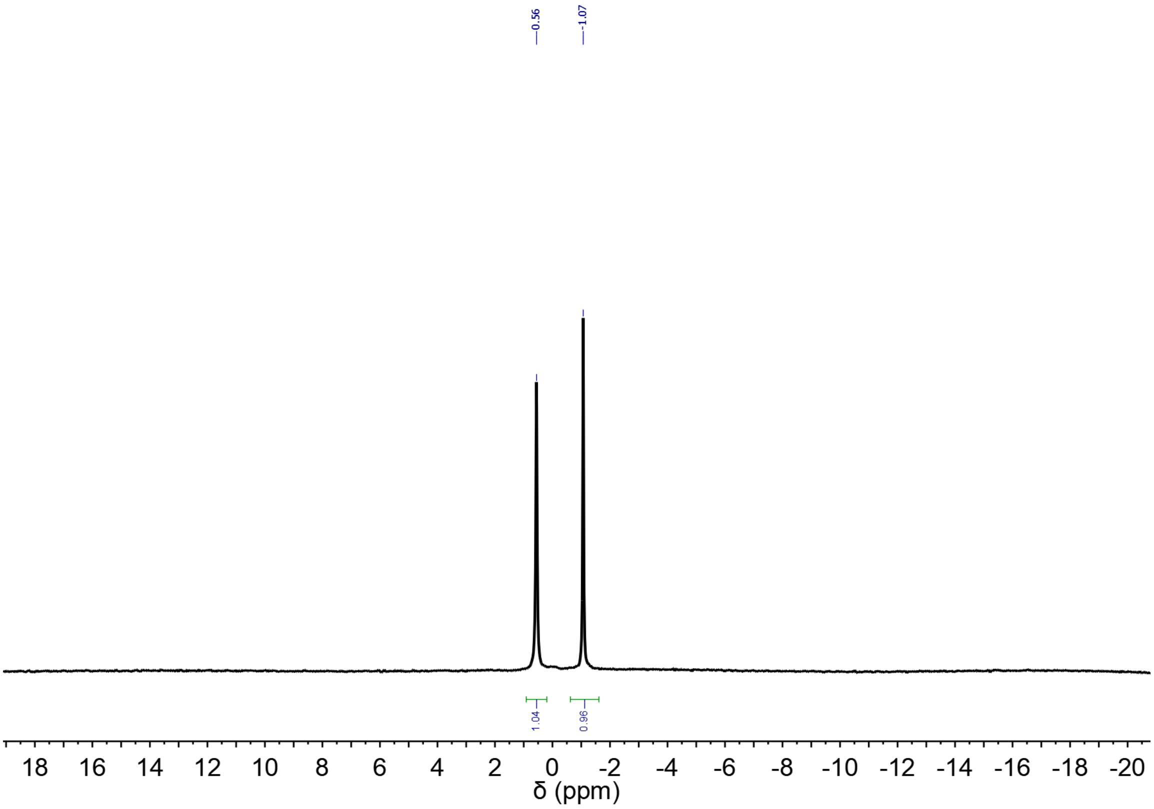

Figure S.92 – ^{11}B NMR (128 MHz, $CDCl_3$, 25°C) spectrum

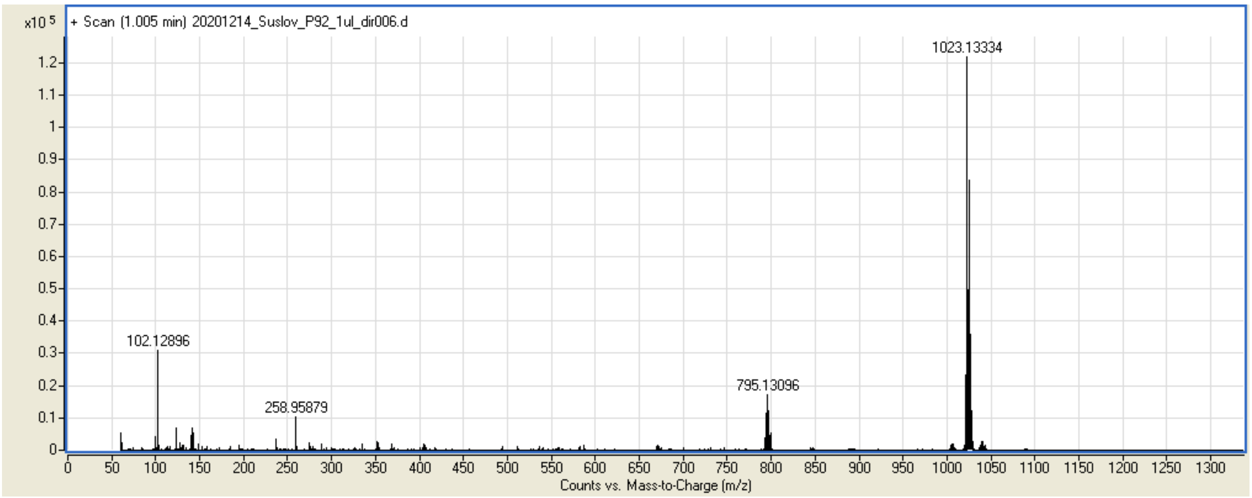

Figure S.93 – ESI-MS spectrum (MeCN, positive ion mode)

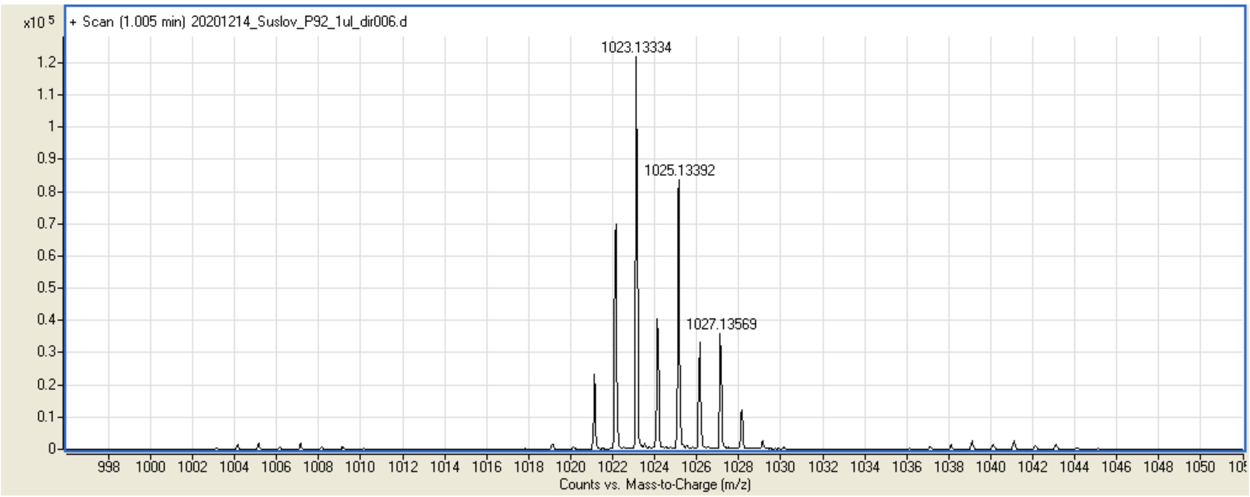

Figure S.94 – ESI-MS spectrum, main Pd cation zoomed (MeCN, positive ion mode)

S.8 PPAs characterization

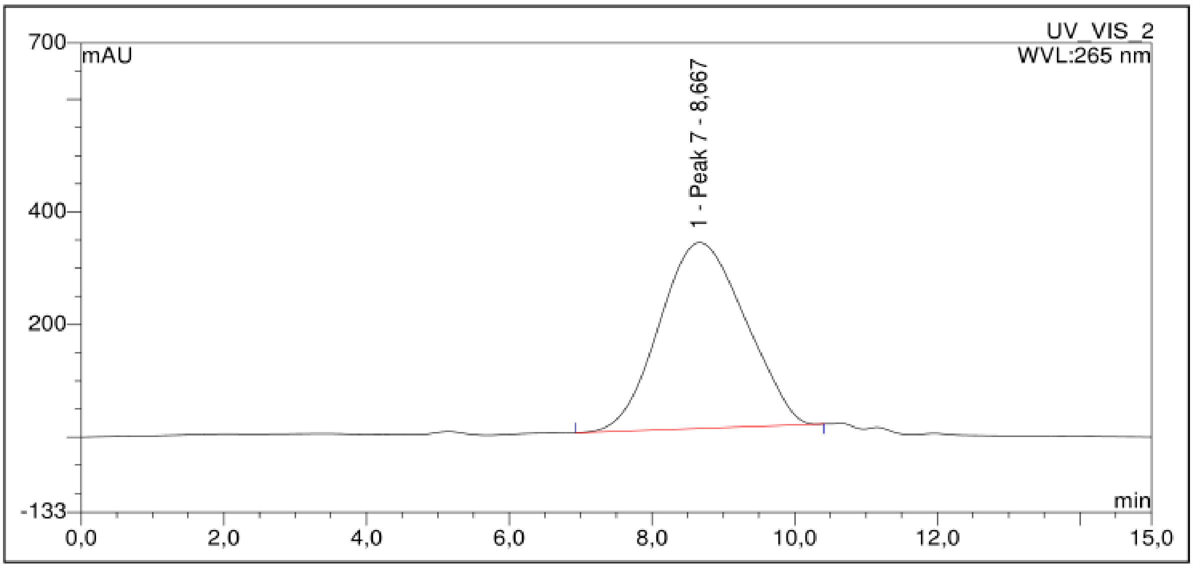

Peaks			
Peak number	1	2	3
Peak name	Peak 7	n.a.	n.a.
**Peak Parameters**			
Ret. Time Peak Start [min]	6.93	n.a.	n.a.
Mw (Peak Start) [Da]	347091	n.a.	n.a.
Ret. Time Peak Maximum [min]	8.67	n.a.	n.a.
Mw (Peak Maximum) [Da]	15482	n.a.	n.a.
Ret. Time Peak End [min]	10.41	n.a.	n.a.
Mw (Peak End) [Da]	682	n.a.	n.a.
Peak Area [rel. units]	460.21	n.a.	n.a.
% Total Peak Area	100.00	n.a.	n.a.
**Averages**			
Number Average Mn	8919	n.a.	n.a.
Weight Average Mw	21380	n.a.	n.a.
Z Average Mz	45110	n.a.	n.a.
(Z+1) Average Mz+1	77866	n.a.	n.a.
**Polydispersity**			
Polydispersity PD	2.40	n.a.	n.a.

Figure S.95 – GPC elution curves recorded from the UV-detector of PPA obtained with **3**, Table 5, Entry 11

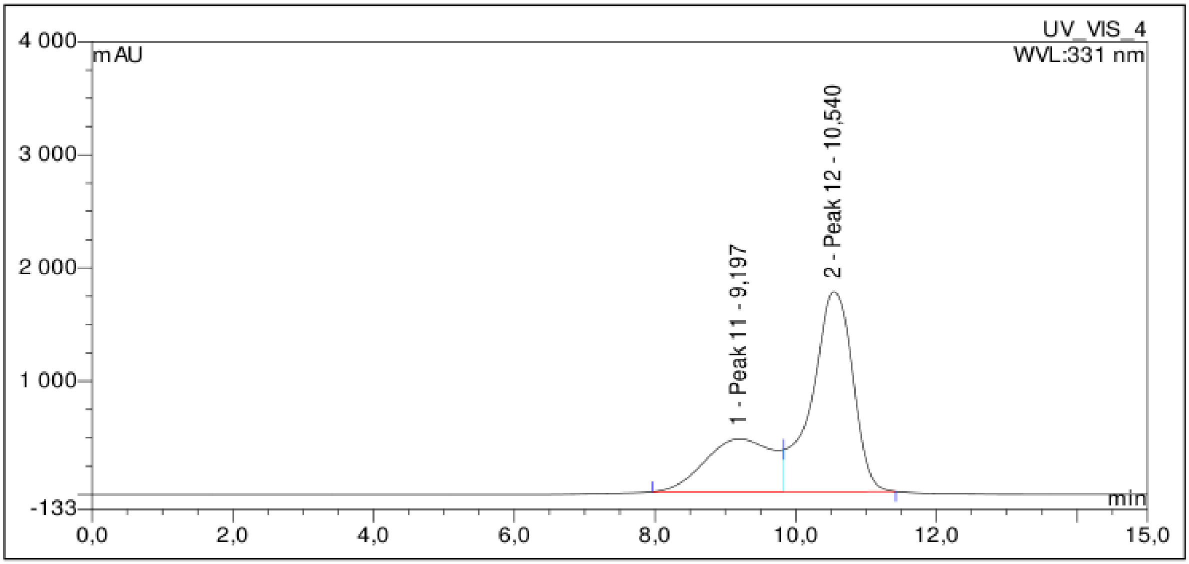

**Peaks**			
Peak number	1	2	3
Peak name	Peak 11	Peak 12	n.a.
**Peak Parameters**			
Ret. Time Peak Start [min]	7,97	9,83	n.a.
Mw (Peak Start) [Da]	53359	1910	n.a.
Ret. Time Peak Maximum [min]	9,20	10,54	n.a.
Mw (Peak Maximum) [Da]	5935	536	n.a.
Ret. Time Peak End [min]	9,83	11,42	n.a.
Mw (Peak End) [Da]	1910	111	n.a.
Peak Area [rel. units]	522,97	1188,31	n.a.
% Total Peak Area	30,56	69,44	n.a.
**Averages**			
Number Average Mn	4964	513	n.a.
Weight Average Mw	7946	655	n.a.
Z Average Mz	12921	846	n.a.
(Z+1) Average Mz+1	18959	1064	n.a.
**Polydispersity**			
Polydispersity PD	1,60	1,28	n.a.

Figure S.96 – GPC elution curves recorded from the UV-detector of PPA obtained with **3**, Table 5, Entry 13

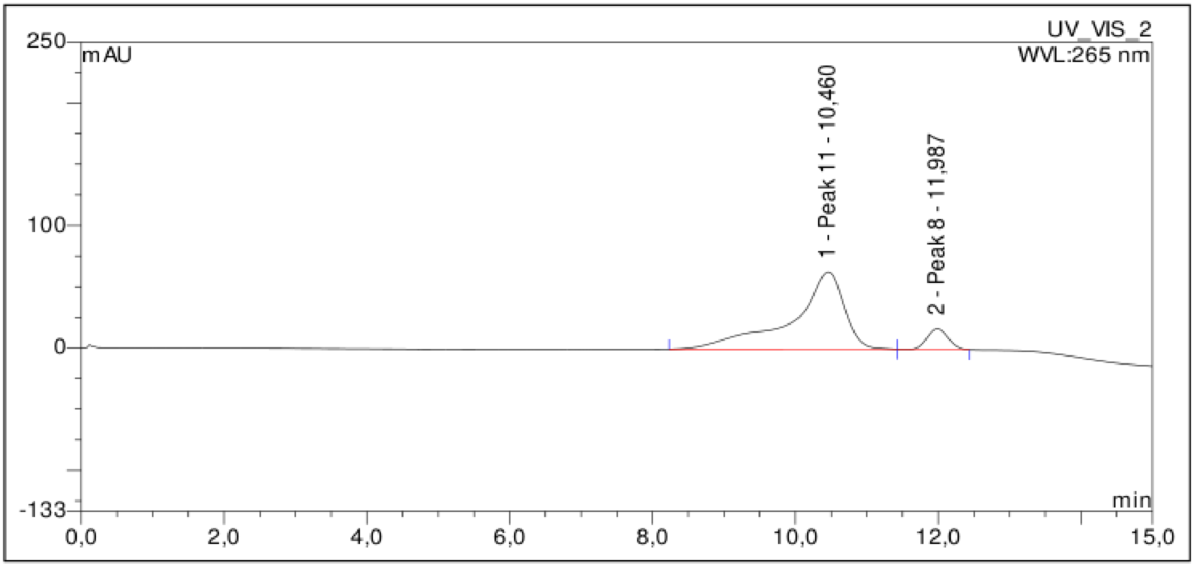

**Peaks**			
Peak number	1	2	3
Peak name	Peak 11	Peak 8	n.a.
**Peak Parameters**			
Ret. Time Peak Start [min]	8,23	11,43	n.a.
Mw (Peak Start) [Da]	33588	111	n.a.
Ret. Time Peak Maximum [min]	10,46	11,99	n.a.
Mw (Peak Maximum) [Da]	628	41	n.a.
Ret. Time Peak End [min]	11,42	12,44	n.a.
Mw (Peak End) [Da]	112	18	n.a.
Peak Area [rel. units]	53,50	5,88	n.a.
% Total Peak Area	90,09	9,91	n.a.
**Averages**			
Number Average Mn	801	39	n.a.
Weight Average Mw	1950	41	n.a.
Z Average Mz	5941	43	n.a.
(Z+1) Average Mz+1	11167	45	n.a.
**Polydispersity**			
Polydispersity PD	2,44	1,06	n.a.

Figure S.97 – GPC elution curves recorded from the UV-detector of PPA obtained with **3**, Table 5, Entry 14

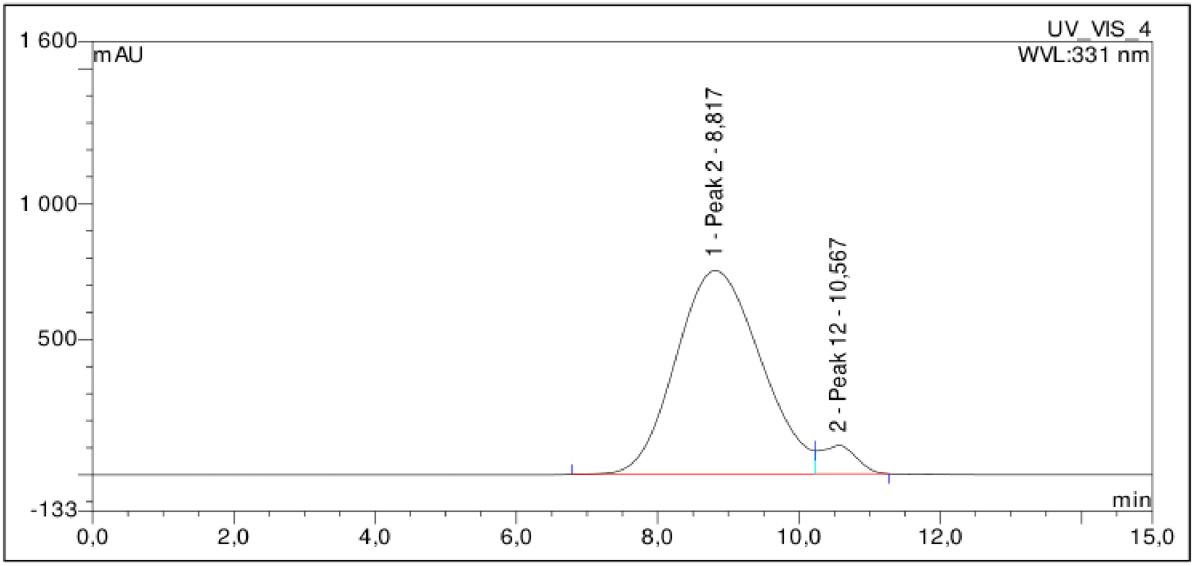

**Peaks**			
Peak number	1	2	3
Peak name	Peak 2	Peak 12	n.a.
**Peak Parameters**			
Ret. Time Peak Start [min]	6,78	10,23	n.a.
Mw (Peak Start) [Da]	446534	928	n.a.
Ret. Time Peak Maximum [min]	8,82	10,57	n.a.
Mw (Peak Maximum) [Da]	11719	511	n.a.
Ret. Time Peak End [min]	10,23	11,27	n.a.
Mw (Peak End) [Da]	939	144	n.a.
Peak Area [rel. units]	1049,04	60,89	n.a.
% Total Peak Area	94,51	5,49	n.a.
**Averages**			
Number Average Mn	6509	468	n.a.
Weight Average Mw	16229	537	n.a.
Z Average Mz	37429	602	n.a.
(Z+1) Average Mz+1	82677	658	n.a.
**Polydispersity**			
Polydispersity PD	2,49	1,15	n.a.

Figure S.98 – GPC elution curves recorded from the UV-detector of PPA obtained with **3**, Table 5, Entry 19

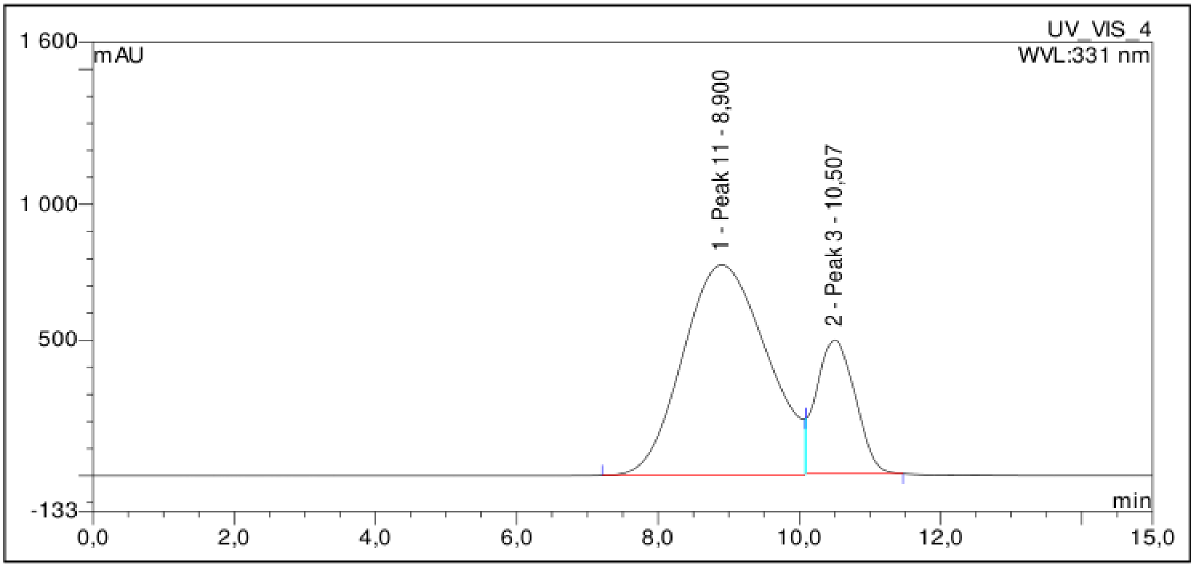

Peaks			
Peak number	1	2	3
Peak name	Peak 11	Peak 3	n.a.
**Peak Parameters**			
Ret. Time Peak Start [min]	7,21	10,10	n.a.
Mw (Peak Start) [Da]	208025	1178	n.a.
Ret. Time Peak Maximum [min]	8,90	10,51	n.a.
Mw (Peak Maximum) [Da]	10095	569	n.a.
Ret. Time Peak End [min]	10,08	11,47	n.a.
Mw (Peak End) [Da]	1214	102	n.a.
Peak Area [rel. units]	1068,27	312,64	n.a.
% Total Peak Area	77,36	22,64	n.a.
**Averages**			
Number Average Mn	5927	492	n.a.
Weight Average Mw	13442	586	n.a.
Z Average Mz	27479	680	n.a.
(Z+1) Average Mz+1	46937	764	n.a.
**Polydispersity**			
Polydispersity PD	2,27	1,19	n.a.

Figure S.99 – GPC elution curves recorded from the UV-detector of PPA obtained with **3**, Table 5, Entry 20

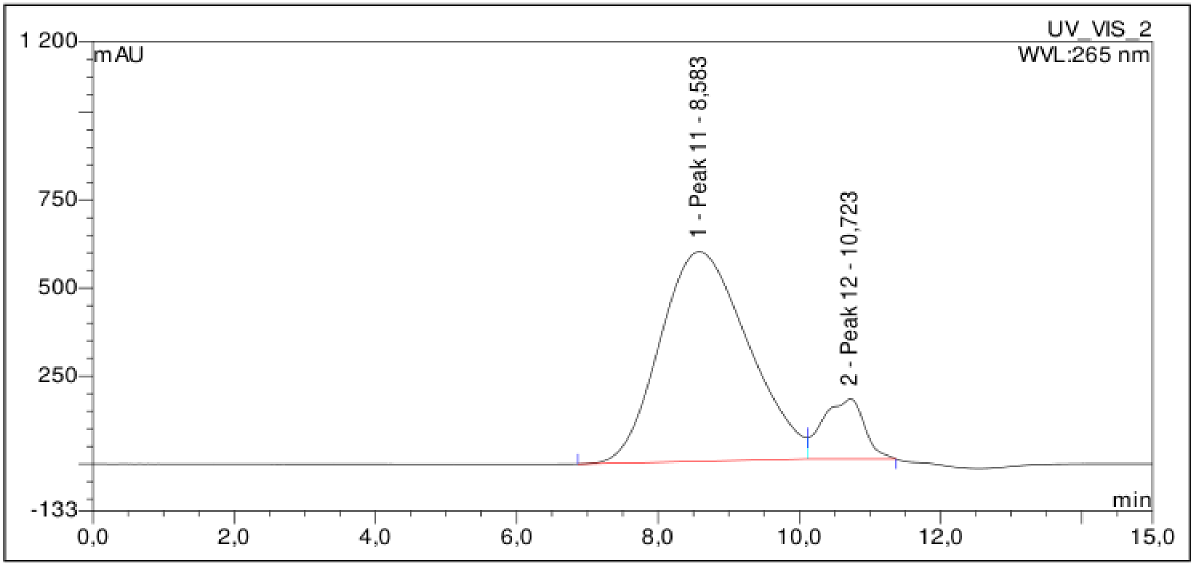

Peaks			
Peak number	1	2	3
Peak name	Peak 11	Peak 12	n.a.
**Peak Parameters**			
Ret. Time Peak Start [min]	6,86	10,12	n.a.
Mw (Peak Start) [Da]	388691	1153	n.a.
Ret. Time Peak Maximum [min]	8,58	10,72	n.a.
Mw (Peak Maximum) [Da]	17968	392	n.a.
Ret. Time Peak End [min]	10,12	11,37	n.a.
Mw (Peak End) [Da]	1153	123	n.a.
Peak Area [rel. units]	860,51	115,87	n.a.
% Total Peak Area	88,13	11,87	n.a.
**Averages**			
Number Average Mn	8843	430	n.a.
Weight Average Mw	23138	523	n.a.
Z Average Mz	48280	623	n.a.
(Z+1) Average Mz+1	80559	717	n.a.
**Polydispersity**			
Polydispersity PD	2,62	1,22	n.a.

Figure S.100 – GPC elution curves recorded from the UV-detector of PPA obtained with **3**, Table 5, Entry 24

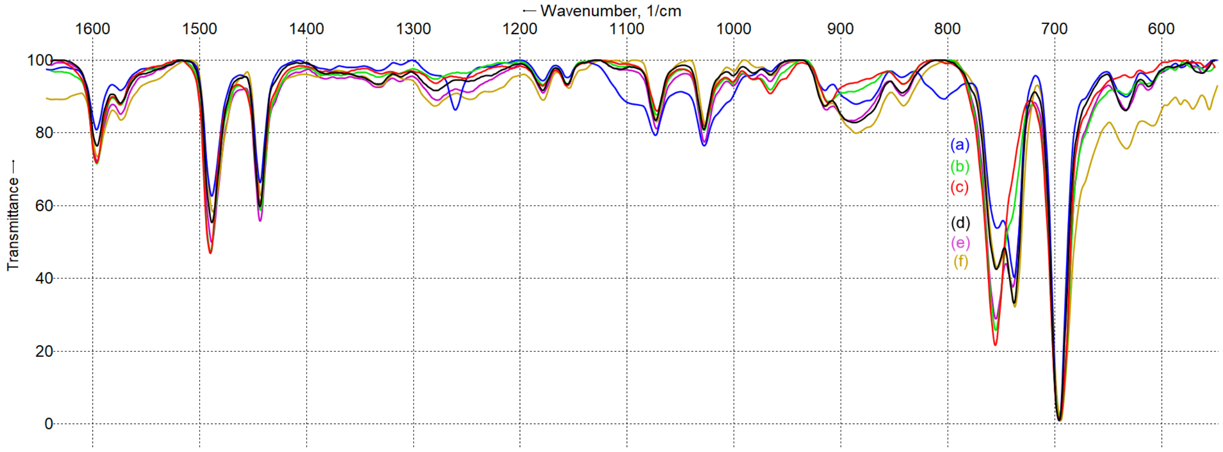

Figure S.101 – FTIR spectra (KBr) of PPAs obtained with **3**, samples reported in Table 5: Entry 11 (a), Entry 13 (b), Entry 14 (c), Entry 19 (d), Entry 20 (e), Entry 24 (f).

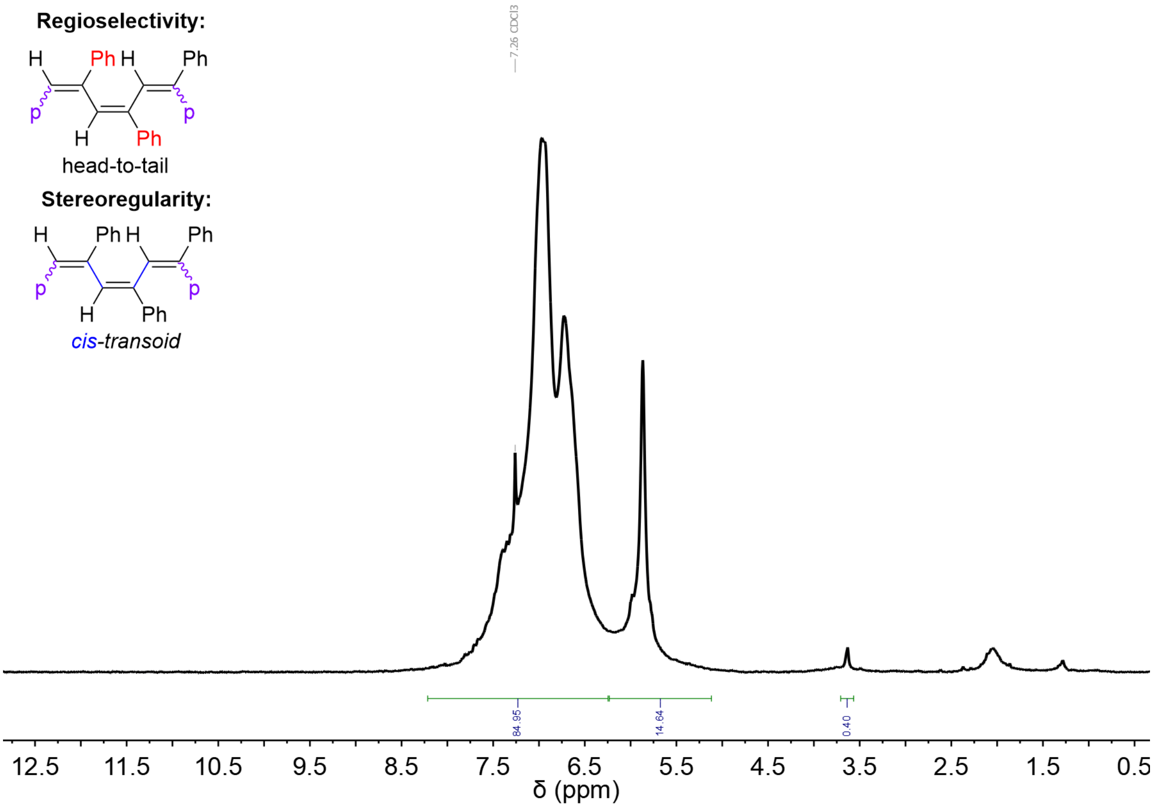

Figure S.102 – ^{1}H NMR (60 MHz, $CDCl_3$, 40°C) spectrum of PPA obtained with **3** at 25°C in THF in $CDCl_3$ (see Table 5, Entry 24)

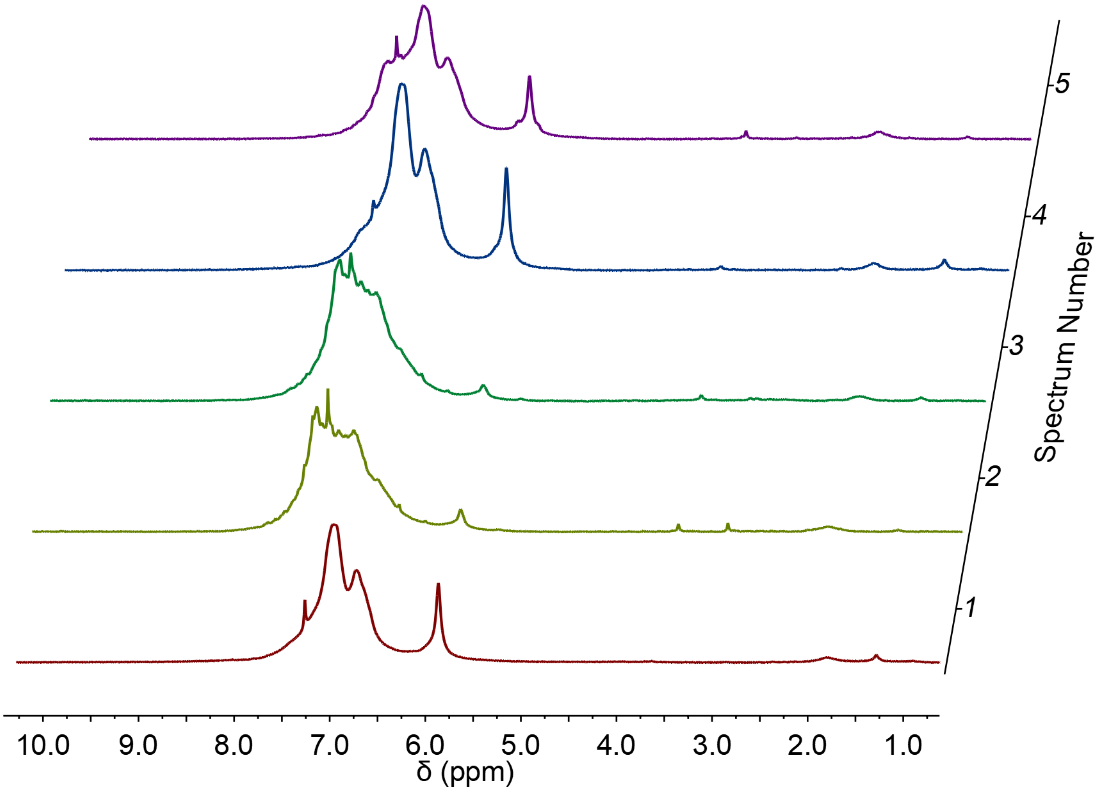

Figure S.103 – ^{1}H NMR (60 MHz, $CDCl_3$, 40°C) spectra of PPAs obtained with **3**, samples reported in Table 5: Entry 11 (1), Entry 13 (2), Entry 14 (3), Entry 19 (4), Entry 20 (5)
